# The Exercise Oncology Knowledge Mobilization Initiative: An International Modified Delphi Study

**DOI:** 10.3389/fonc.2021.713199

**Published:** 2021-07-19

**Authors:** Scott C. Adams, Jenna Smith-Turchyn, Daniel Santa Mina, Sarah Neil-Sztramko, Prue Cormie, S. Nicole Culos-Reed, Kristin L. Campbell, Gemma Pugh, David Langelier, Kathryn H. Schmitz, David J. Phipps, Michelle Nadler, Catherine M. Sabiston

**Affiliations:** ^1^ Department of Cardiology, Toronto General Hospital Research Institute, Toronto, ON, Canada; ^2^ Ted Rogers Cardiotoxicity Prevention Program, Peter Munk Cardiac Centre, Toronto, ON, Canada; ^3^ Faculty of Kinesiology & Physical Education, University of Toronto, Toronto, ON, Canada; ^4^ School of Rehabilitation Science, McMaster University, Hamilton, ON, Canada; ^5^ Faculty of Medicine, University of Toronto, Toronto, ON, Canada; ^6^ Department of Anesthesia and Pain Management, Toronto General Hospital, Toronto, ON, Canada; ^7^ Department of Health Research Methods, Evidence and Impact, McMaster University, Hamilton ON, Canada; ^8^ Peter MacCallum Cancer Centre, Melbourne, VIC, Australia; ^9^ Sir Peter MacCallum Department of Oncology, The University of Melbourne, Melbourne, VIC, Australia; ^10^ Faculty of Kinesiology, University of Calgary, Calgary, AB, Canada; ^11^ Department of Oncology, Cumming School of Medicine, University of Calgary, Calgary, AB, Canada; ^12^ Department of Physical Therapy, Faculty of Medicine, University of British Columbia, Vancouver, BC, Canada; ^13^ National Child Cancer Network, Auckland, New Zealand; ^14^ Cancer Rehabilitation and Survivorship, Princess Margaret Hospital, Toronto, ON, Canada; ^15^ College of Medicine, Penn State University, Hershey, PA, United States; ^16^ Division of Vice-President Research & Innovation, York University, Toronto, ON, Canada; ^17^ Medical Oncology, Princess Margaret Cancer Centre, Toronto, ON, Canada; ^18^ Department of Medicine, University of Toronto, Toronto, ON, Canada

**Keywords:** exercise, clinical oncology, knowledge translation, implementation science, standard of care

## Abstract

**Introduction:**

Exercise is vital to health and well-being after a cancer diagnosis yet is poorly integrated in cancer care. Knowledge mobilization (KM) is essential to enhance exercise opportunities. We aimed to (1) develop and refine a list of highly important exercise oncology research and KM themes and (2) establish the relative importance of the themes for supporting the implementation of exercise as a standard of care for people living with and beyond cancer.

**Methods:**

Informed by the Co-Produced Pathway to Impact KM framework, a modified Delphi study approach was used to develop, rate, and rank exercise oncology research and KM themes through an international stakeholder workshop and a three-round iterative online survey. Open-ended stakeholder feedback from cancer survivors, healthcare practitioners (HCPs), qualified exercise professionals (QEPs), policy makers, and researchers was used to update themes between survey rounds. Themes were ranked from highest to lowest importance and agreement was examined across all stakeholders and within stakeholder groups.

**Results:**

A total of 269 exercise oncology stakeholders from 13 countries participated in the study. Twelve final exercise oncology research and KM themes were produced. The final top ranked research themes were related to: (1) QEP integration into primary cancer care teams, (2) Exercise oncology education for HCPs, and (3) Accessibility of cancer exercise programs & support services. There was statistically significant agreement between stakeholders (*p*<0.001) and within stakeholder groups (*p*’s≤0.02) on the general rankings of themes (i.e., some themes generally ranked higher and lower compared to others). Low Kendall’s *W* statistics indicated variability related to the specific ranked order of the themes between stakeholders and within stakeholder groups. Moreover, there were key differences in the rankings for specific themes between policy makers and other stakeholder groups that highlight potentially important discordance in the research and KM priorities for policy makers that warrants further study.

**Conclusion:**

These findings can be used to guide initiatives and align stakeholders on priorities to support exercise implementation as a standard of cancer care. Additional research is needed to better understand the differences in the proposed research and KM priorities across stakeholders.

## Introduction

The past two decades have seen a substantial increase of research in exercise oncology. Exercise training is defined as regular, structured sessions of aerobic and/or resistance exercise aimed at improving health and fitness (1). Exercise is an important part of chronic disease prevention, treatment, and management, including cancer (2). Based on evidence from systematic reviews and meta-analyses in cancer survivors, exercise is associated with improvements in diverse outcomes, such as decreased fatigue (3), adverse treatment effects (4), depression (5), psychosocial distress (4) and increased muscle strength (6), cardiorespiratory fitness (7), health-related quality of life (8), cognitive function (4), and survival (4). These data provide compelling evidence that exercise should be included in cancer care to prevent and mitigate adverse treatment effects, improve diverse physical and psychosocial outcomes, and reduce mortality.

To this end, the Clinical Oncology Society of Australia released a position statement endorsing the inclusion of exercise training as a standard of care for all patients with cancer (9). In North America, regional [e.g., Cancer Care Ontario (CCO)] (10) and national [American College of Sports Medicine (ACSM)] (11) organizations recommend that cancer patients avoid inactivity and engage in regular exercise. Pathways for exercise care have been proposed to foster recommendation and support (12, 13). Despite these endorsements, notable knowledge and infrastructure gaps preclude knowledge mobilization (KM) and implementation of exercise as a standard of cancer care across the world, including countries with a range of public and private-payer healthcare systems. To date, studies have identified lack of healthcare practitioner (HCP) knowledge and training (14–16) as well as institutional- and healthcare system-level barriers (e.g. funding and infrastructure) (16, 17) as major barriers to implementation of clinical and community-based exercise support services for cancer survivors.

There is an urgent need for research and KM (also known as knowledge translation) aimed at better understanding the best practice solutions to implementing exercise support as a standard component of care for cancer survivors. Optimal engagement and collaboration across exercise oncology stakeholder groups is critical to facilitating this work. To this end, frameworks like the Co-Produced Pathway to Impact (CPPI) (18) are designed to inform the development and conduct of collaborative research and KM. As such, the purpose of the Exercise Oncology Knowledge Mobilization Initiative (ExOnc-KMI) is to define a strategic agenda for collaborative research and KM to support implementation of exercise as a standard of cancer care. The objectives of the study were to (1) develop and refine a list of highly important exercise oncology research and KM themes, and (2) prioritize these themes according to their relative importance towards implementing exercise support services as a standard of care for people living with and beyond cancer.

## Materials and Methods

The ExOnc-KMI used a modified Delphi study approach (19) that consisted of an international stakeholder workshop and three rounds of an iterative online Delphi survey. The CPPI is a logic model-based KM framework used to map/plan the progress of research, dissemination, uptake, implementation, and impact (see **Online Supplement 1**) (18). The CPPI framework was used to guide the definition and scope of the themes developed at the stakeholder workshop. Specifically, following Phipps et al. (20), themes were defined according to four elements (i.e., title, goals, stakeholders, and impacts). The themes and their constituent elements were then evaluated (i.e., rated and ranked) and refined (via open-ended feedback from stakeholders) during the modified Delphi survey. The University of Toronto Research Ethics board approved the study protocol (ID: 38311). Informed consent was completed prior to the completion of the first survey round.

### Stakeholder Workshop

**Figure 1:**
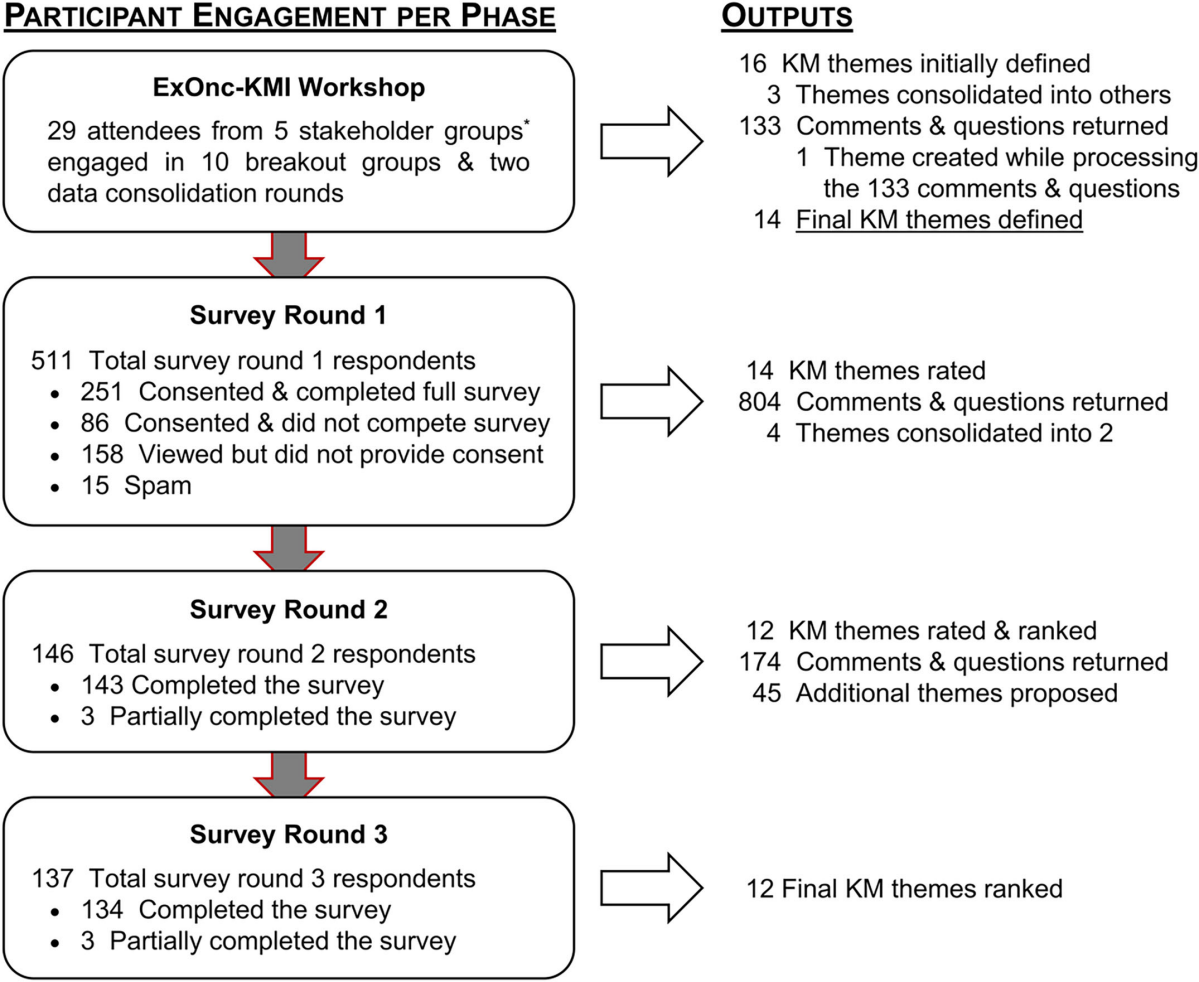
Participant flow and outputs per study phase. KM, knowledge mobilization. *Stakeholder Group Definitions: Healthcare providers [HCPs; i.e., members of any allied health profession (e.g., Dieticians, Kinesiologists, Nurses, Physicians, Social Workers)];** Policy makers** [e.g., program-, department-, & institute level administrators within primary → tertiary healthcare settings; Persons within all levels of government (municipal → federal)];** Qualified exercise professionals** (QEPs; e.g., kinesiologists, physiotherapists);** Researchers** (e.g., behavioural, medical, psychosocial, rehabilitation);** Survivors & Support persons** (i.e., any person still alive following a cancer diagnosis & any person who supports them (e.g., friends, family, colleagues).

The ExOnc-KMI stakeholder workshop was held in November 2018 (Toronto, ON). Exercise oncology stakeholders from five primary stakeholder groups [(1) policy makers and healthcare/health organization administrators (hereafter policy makers), (2) HCPs, (3) cancer survivors and support persons, (4) qualified exercise professionals (QEPs), and (5) researchers; **Figure 1**] were invited to attend the workshop. The objective of the workshop was to identify, develop, refine, and discuss an initial list of high priority themes for exercise oncology research and KM to support the implementation of exercise as a standard of cancer care in clinical and community settings. After introducing the study, breakout sessions divided participants into groups of five to six participants (balanced across stakeholder groups), in two sequential rounds. During each round, groups were asked to brainstorm, discuss, and define themes for exercise oncology research and KM according to the four CPPI-defined elements: title, goals, stakeholders, impacts (18, 20). Following both breakout rounds, a data consolidation process was facilitated by a KM expert (DP). The elements of the individual themes were discussed by attendees and clarified as needed, and similar themes were combined *via* group consensus. Individual stakeholders were then asked to independently review and submit questions, comments, and concerns for each of the consolidated themes. Following the workshop, study team members (SA, JST) reviewed and consolidated all workshop data into a list of 14 themes for exercise oncology research and KM defined by the CPPI elements. The themes were defined through consensus and discussion with study team leads CS and DSM. To increase the relevance of the findings, this list was distributed internationally to a broader group of international exercise oncology stakeholders *via* a modified Delphi survey.

### Modified Delphi Survey

Purposeful and snowball sampling (21) methods were used to identify potential survey respondents. First, workshop attendees were asked to personally invite three to four individuals from each of the five stakeholder groups *via* a standardized recruitment email. Second, participants were encouraged to share a link to the study with exercise oncology stakeholders within their network. The link directed participants to information about the study, including the informed consent form. If the respondent provided informed consent, the survey was initiated (Survey Monkey. Palo Alto, CA). The first round of surveys was completed between December 2019 and March 2020, with rounds 2 and 3 completed in October – December 2020 and January 2021 – March 2021, respectively. Respondents to each survey round were invited to participate in the subsequent survey round(s). Reminders to complete each survey round were provided at 30 days and at 14 days prior to the close of each survey.

#### Round 1

Demographic data were collected to describe the study sample and verify that respondents were associated with at least one of the primary stakeholder groups. If respondents could not be verified as an eligible stakeholder, their data were excluded from the study (see Spam; **Figure 1**). In round 1, participants were asked to review and rate each of the 14 themes for exercise oncology research and KM defined at the workshop by perceived importance using a 5-point Likert scale (1=not important to 5=very important). For each theme, participants were asked “How important is the following research theme to supporting the implementation and permanent adoption of exercise as a standard of care for cancer patients and survivors?”. Participants were also invited to critique, question, and suggest improvements for each theme using open textboxes. Once the round 1 survey closed, investigators (SA, JST) analyzed the ratings, consolidated the open-ended responses according to the four CPPI elements, and updated the themes using this feedback for re-evaluation in round 2. Only themes with mean ratings ≥4/5 were included in the subsequent survey (22, 23).

#### Round 2

In round 2, participants were presented with the average theme importance ratings from round 1 and the updated list of themes. To help respondents differentiate between themes that generally improve outcomes for cancer survivors and those that specifically support exercise implementation (i.e., the purpose of this study), participants were asked to rate the importance of each updated theme in terms of (1) how important the theme’s impacts may be to improving outcomes for cancer survivors and (2) how important the theme’s impacts may be to supporting the implementation of exercise as a standard part of cancer care. To increase the resolution of the ratings, themes were rated on a 7-point Likert scale (ranging from 1=not at all important to 7=extremely important). Participants were also asked to: (1) critique and provide feedback for each theme; (2) rank the importance of the remaining themes from most to least important; and, (3) suggest additional themes for exercise oncology research and KM. Once the round 2 survey closed, investigators (SA, JST) analyzed the ratings, consolidated the feedback, and updated the themes for distribution in the round 3 survey. Only themes with mean ratings ≥5 out of 7 were included in the final survey.

#### Round 3

In round 3, participants were presented with the findings from round 2. Participants were asked for a final ranking of the 12 updated themes, with consideration of two key questions: (1) “How likely would achieving the impacts (outcomes) for each Theme influence whether exercise is adopted by the healthcare system as a standard part of cancer care?”; and (2) “How potentially impactful is each Theme compared to others?”.

These questions were prompts for participants to consider the broader context of exercise, oncology, and KM.

### Data Processing and Analysis

#### Importance Ratings and Feedback Integration

Following survey rounds 1 and 2, mean importance ratings were calculated by adding the ratings for each theme and dividing by the total number of respondents per round. All open-ended feedback was independently categorized by investigators (SA, JST) as being (1) related to one of the four CPPI elements (i.e., title, goals, stakeholders, impacts), (2) a generally relevant response [e.g., “It is important to clarify how you want to develop this strategy (web-based, mobile app, text message…)”], (3) an unactionable response (e.g., “Always room to improve”), or (4) related to a different theme. Investigators then met to review and discuss responses and update the scope (e.g., goals and impacts) and content (e.g., wording and definitions) for all themes. Conflicting feedback (e.g., two participant responses suggesting expanding *vs.* narrowing the scope of a theme) was adjudicated (SA, JST) and suggestions best aligned with the objectives of the study were incorporated into the updated themes.

#### Priority Rankings

First, the priority rankings for each theme were reverse scored (i.e., one point for the lowest ranked item and full points to the highest ranked item) for each individual respondent. Mean priority rankings for each theme were calculated. Kendall’s coefficient of concordance (*W*) was used to evaluate the level of agreement (0 = no agreement to 1 = complete agreement) regarding the round 3 rankings for all themes in the full sample and within individual stakeholder groups. Acknowledging the multiple roles that participants may have hold, respondents were included in the self-identified stakeholder groups they represented (e.g., one participant’s rankings could be included in both the ‘survivor and support persons’ group and/or the ‘policy makers’ group and/or the ‘researchers’ group, for analyses).

## Results

Participant characteristics across study phases are presented in **Table 1**. Briefly, exercise oncology stakeholders (*n*=269) from 13 countries participated in the study. Across individual survey rounds, the mean age of participants ranged from 39.3 to 40.3 years; the percentage of participants self-identifying as female ranged from 69-77%; and, the percentage of participants self-identifying as belonging to individual stakeholder groups ranged from 16% to 31% for HCPs, 5% to 18% for policy makers, 31% to 50% for QEPs, 32% to 52% for researchers, and 14% to 38% for survivors and support persons. Stakeholder totals exceed 100% as participants could identify as belonging to more than one stakeholder group [*n* = 91 (34%) of participants indicated belonging to more than one stakeholder groups]. See **Figure 1** for an overview of study phases, participant flow, and outcomes.

**Table 1:** Participant Characteristics.

Characteristics	Workshop		Round 1		Round 2		Round 3	
	No.	%	No.	%	No.	%	No.	%
Total participants	29		251		146		137*	
**Stakeholders**								
Healthcare providers	9	31	60	24	26	18	22	16
Policy makers	5	17	13	5	12	8	25	18
Qualified exercise professionals	9	31	125	50	70	48	53	39
Researchers	15	52	94	37	54	37	44	32
Survivors & Support persons	4	14	78	31	55	38	48	35
**Demographics**								
Age [mean (SD)]	–	–	39.9	(10.5)	39.3	(10.3)	40.3	(10.7)
Sex								
Female	20	69	191	76	112	77	98	72
Male	9	31	60	24	34	23	22	16
Not Reported	0	0	0	0	0	0	17^†^	12
Country								
Australia	1	3	13	5	4	3	3	2
Canada	25	86	102	41	71	49	62	45
Germany & Austria	0	0	3	1	3	2	3	2
Other European (Denmark, Sweden, Netherlands)	0	0	8	3	4	3	3	2
United Kingdom (England, Ireland, Scotland)	1	3	56	22	38	26	31	23
United States	2	7	67	27	24	16	18	13
Other (Brazil, Turkey)	0	0	2	1	2	1	0	0
Not Reported	0	0	0	0	0	0	17^†^	12

*116 original respondents + 17 supplemental policy maker respondents.

^†^Demographic data was not collected from supplemental policy maker respondents.

### Stakeholder Workshop

Twenty-nine stakeholders defined 16 research and KM themes during a total of ten breakout groups during the workshop (**Figure 1**). Three themes were incorporated into the 13 others and one additional theme was created to capture unaddressed aspects of the 133 individual responses from workshop participants (see Individual Response Categories, **Online Supplement 2**). The workshop-derived themes and individual responses were processed and consolidated into the 14 themes presented in round 1 of the modified Delphi survey.

### Delphi Survey

#### Round 1

Of respondents who provided consent (*N*=337), 251 (75%) participants completed the round 1 survey. All 14 of the themes had mean importance rating scores 4 or more out of 5 (range: 4.0 to 4.7; **Table 2**). Respondents returned 804 comments and questions (**Online Supplement 3**) that were processed and used to update items for round 2, including suggestions to merge four themes into two [i.e., Themes D and E (Survey 1) merged into Theme D (Survey 2); Themes H and K (Survey 1) merged into Theme G (Survey 2); see **Table 2** for details]. See **Online Supplement 4** for changes to all theme elements across all three survey rounds.

**Table 2:** Exercise oncology KM research ratings & rankings from Delphi survey rounds 1-3.

Survey Round (R) 1: Original Theme Labels & Titles	R1 Rating		R2 Q1. Rating		R2 Q2. Rating		R2 Ranking*		R3 Ranking**	
	Mean (/5)	%	Mean (/7)	%	Mean (/7)	%	Mean	Order	Mean	Order
	(95% CI)		(95% CI)		(95% CI)					
Theme A (R1): Messaging strategies to support cancer survivors’ exercise engagement	4.4	89	6.1	87	6.1	87	7.1	9	7.3	11
	(4.4-4.5)		(5.9-6.3)		(5.9-6.3)					
Theme B (R1): Exercise oncology education models for oncologists & primary care teams	4.7	93	6.2	89	6.4	92	5.4	3	5.4	2
	(4.6-4.8)		(6.0-6.4)		(6.3-6.6)					
Theme C (R1): Standardized exercise oncology training for diverse exercise professionals across various training environments	4.5	89	5.9	84	6.0	85	6.2	5	6.1	5
	(4.4-4.5)		(5.7-6.1)		(5.8-6.2)					
Theme F (R1): Qualified exercise professional integration into primary cancer care teams	4.5	89	6.0	85	6.1	88	4.7	1	5.2	1
	(4.4-4.6)		(5.8-6.1)		(6.0-6.3)					
Theme G (R1): Referral mechanisms to clinical- & community-based cancer exercise programs	4.6	91	6.0	86	6.2	88	5.3	2	6.1	4
	(4.5-4.6)		(5.8-6.2)		(6.0-6.3)					
Theme I (R1): Exercise oncology resource sharing across academic & community partners	4.2	83	5.3	75	5.5	79	8.4	12	7.0	9
	(4.1-4.3)		(5.1-5.5)		(5.3-5.7)					
Theme J (R1): Cancer survivor transitions from clinical to community exercise settings	4.5	89	5.6	80	5.4	77	7.2	10	6.9	8
	(4.4-4.5)		(5.4-5.8)		(5.2-5.6)					
Theme L (R1): Safety & efficacy of community-based cancer exercise support services	4.2	83	5.8	82	5.8	82	6.3	6	7.8	12
	(4.1-4.3)		(5.6-6.0)		(5.6-6.0)					
Theme M (R1): Diverse approaches to facilitate exercise engagement in cancer survivors	4.1	83	5.6	80	5.3	75	6.8	8	7.0	10
	(4.0-4.2)		(5.4-5.8)		(5.0-5.5)					
Theme N (R1): High-priority ‘patient-level’ & ‘economic’ outcomes for community health administrators (e.g., federal, institutional)	4.5	89	5.2	74	5.9	85	6.7	7	6.9	7
	(4.4-4.6)		(4.9-5.4)		(5.7-6.1)					
*Theme D* (R1)*: Novel approaches for supporting hard to reach & under-studied cancer populations with exercise*	4.3	86	5.8	83	5.4	77	8.1	11	6.8	6
	(4.2-4.4)									
*Theme E* (R1)*: Technology-based exercise support strategies for diverse demographic & geographic communities of survivors*	4.2	85	(5.7-6.0)		(5.2-5.6)					
	(4.1-4.3)									
*Theme H* (R1)*: Cancer exercise program availability & accessibility*	4.6	92	6.2	88	6.0	85	5.9	4	5.5	3
	(4.5-4.7)									
*Theme K* (R1)*: Standardized community cancer exercise programming*	4.0	80	(6.0-6.3)		(5.8-6.2)					
	(3.9-4.1)									

CI, confidence interval; Q1, Question 1: How important is this research priority for helping cancer survivors benefit from exercise support?; Q2, Question 2: How important is this research priority for helping exercise support becoming a standard part of care for cancer survivors?; R1, round 1; R2, round 2; Italicized (R1) theme titles denotes the two pairs of themes that were merged into the single themes opposite the themes in R2.

*Kendall’s W=0.091, χ2(11)=143.66; p<0.001); **Kendall’s W=0.050, χ2(11)=71.90; p<0.001).

#### Round 2

One hundred and forty-six participants returned surveys during round 2 (response rate 58%). All 12 themes had mean importance rating scores 5 or more out of 7 for Question 1 (range: 5.2 to 6.2; i.e., theme’s importance towards improving outcomes for survivors) and Question 2 (range: 5.3 to 6.4; i.e., theme’s importance towards supporting the adoption of exercise as a standard of cancer care). The top five ranked themes (in order; highest to lowest) were: (1) QEP integration into primary cancer care teams; (2) Referral pathways & mechanisms for cancer survivors into medically supervised & community-based cancer exercise programs; (3) Evidence-based exercise oncology education models for HCPs working with cancer survivors; (4) Accessibility of medically supervised & community-based cancer exercise programs & support services to meet the needs of diverse groups of cancer survivors; and, (5) Standards for exercise oncology training for QEPs across training (i.e., educational) environments (**Table 2**). Respondents provided 174 comments and questions related to the 12 themes in the round 2 survey that were processed and used to update the themes. An additional 45 complete research and KM suggestions were submitted for consideration (**Online Supplement 5**). All 45 themes were carefully adjudicated and determined to be captured within the broader scope of the 12 existing themes.

#### Round 3

**Figure 2:**
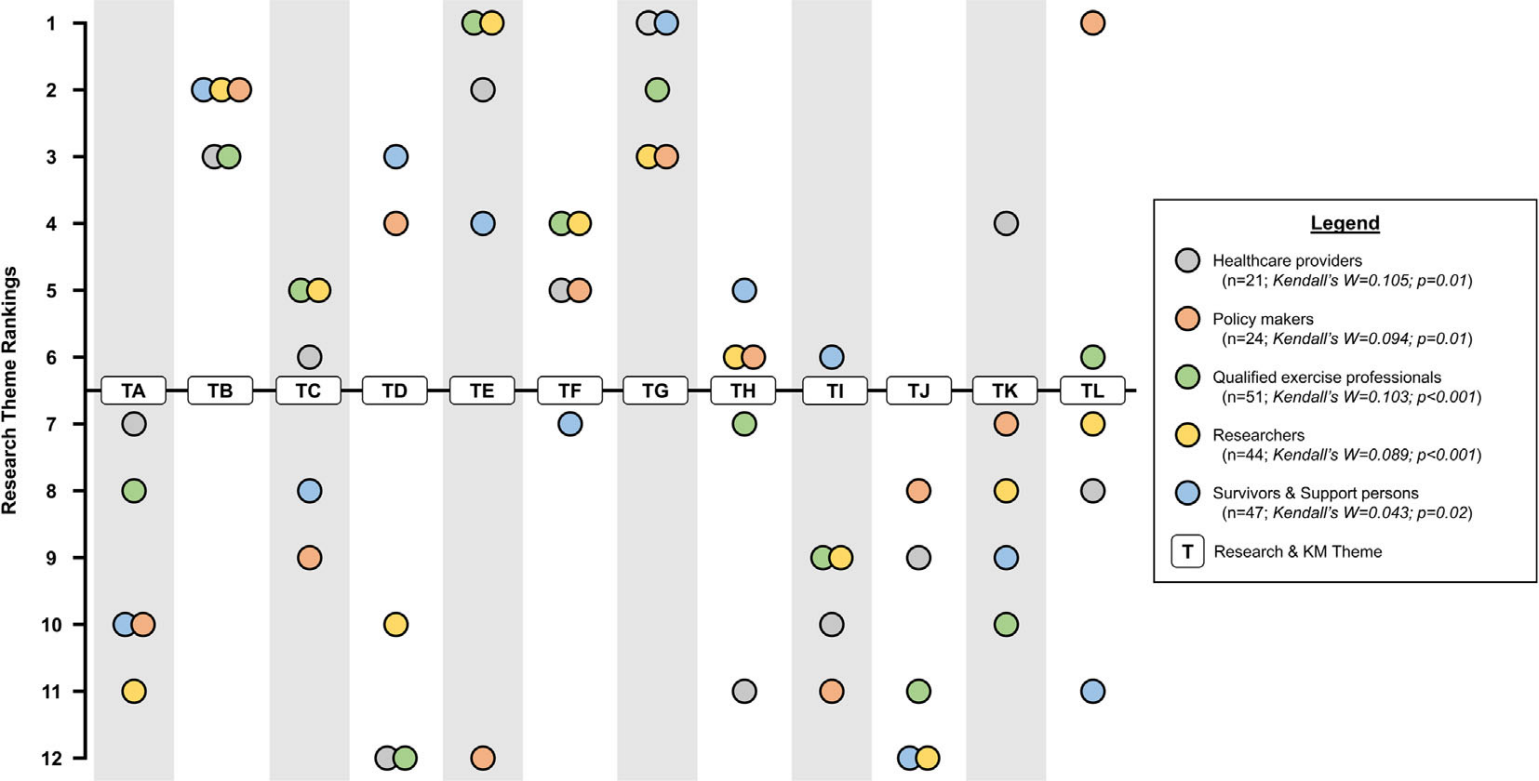
Research theme ranking per stakeholder group. Kendal’s W values reflect degree of agreement within individual stakeholder groups. **Survey Round 3 Titles: TA** = Enhancing communication strategies to increase cancer survivors’ exercise engagement throughout the survivorship trajectory; **TB** = Developing & promoting evidence-based exercise oncology education models for HCPs working with cancer survivors; **TC** = Establishing exercise oncology training standards for QEPs across training environments; **TD** = Enhancing technology-based strategies to improve the delivery of exercise support to demographically-, culturally-, & geographically diverse communities of cancer survivors; **TE** = Integrating QEPs into primary cancer care teams; **TF** = Establishing resources for referring cancer survivors between medical- & community-based cancer exercise services; **TG** = Improving accessibility of medically supervised & community-based cancer exercise support services for diverse groups of cancer survivors; **TH** = Developing & sharing of evidence-based resources to support academic & community partners in providing exercise services for cancer survivors; **TI** = Improving cancer survivor transitions across medically supervised, community-based, & self-directed exercise settings; **TJ** = Establishing the appropriateness & benefits of community-based cancer exercise support services; **TK** = Optimizing approaches & resources to facilitate sustained exercise behaviour change in cancer survivors; **TL** = Understanding the high-priority ‘patient-level’ & ‘economic’ outcomes for healthcare funders & decision-makers.

One hundred thirty-seven of 167 invited participants (146 round 2 respondents, 2 additional stakeholders, and 19 administrators) returned surveys in round 3 (response rate 82%). Of the 12 themes ranked, the top five (in order from highest to lowest) were: (1) QEP integration into primary cancer care teams; (2) Evidence-based exercise oncology education models for HCPs working with cancer survivors; (3) Accessibility of medically supervised & community-based cancer exercise programs & support services to meet the needs of diverse groups of cancer survivors; (4) Referral pathways & mechanisms for cancer survivors into medically supervised & community-based cancer exercise programs; and, (5) Standards for exercise oncology training for QEPs across training environments (**Table 2**). There was statistically significant agreement between all stakeholders’ (*n*=132) general rankings of the themes (i.e., generally higher *vs.* lower ranking themes; *W*=0.050, χ^2^(11)=71.90, *p*<0.001). Low Kendall’s *W* statistic indicated variability related to the specific ranked order of the themes between stakeholders. For example, the theme related to ‘Qualified exercise professional integration into primary cancer care teams’ was consistently one of the highest-ranking priorities between stakeholders with variability regarding whether it specifically ranked first through sixth. Whereas the theme related to ‘Exercise oncology resource sharing across academic and community partners’ was consistently one of the lowest-ranked priorities between stakeholders with ranking position falling from seventh through twelfth position for different stakeholder groups. See **Table 3** for complete final definitions of all themes and constituent elements ranked from highest to lowest. Relative theme rankings between and within stakeholder groups were more consistent for select themes (**Figure 2**). For instance, the ‘Qualified Exercise Professional integration into primary cancer care teams’, ‘Evidence-based exercise oncology education models for HCPs working with cancer survivors’ and ‘Accessibility of medically supervised & community-based cancer exercise programs & support services to meet the needs of diverse groups of cancer survivors’ themes were consistently ranked higher between and within stakeholder groups. Similarly, two themes were consistently ranked lower between and within stakeholder groups, including ‘Communication strategies to support cancer survivors’ exercise engagement throughout the survivorship trajectory’ and ‘Feasibility, safety, efficacy, & effectiveness of appropriate community-based cancer exercise support services’. There was statistically significant agreement in the general rankings of the themes within the stakeholder groups [HCPs (*n*=21), Kendall’s *W*=0.105, χ^2^(11)=24.26, *p*=0.01; Policy makers (*n*=24), Kendall’s *W*=0.094, χ^2^(11)=24.71, *p*=0.01; QEPs (*n*=51), Kendall’s *W*=0.103, χ^2^(11)=57.78, *p*<0.001; Researchers (*n*=44), Kendall’s *W*=0.089, χ^2^(11)=42.97, *p*<0.001; Survivors & Support persons (*n*=47), Kendall’s *W*=0.043, χ^2^(11)=22.16, *p*=0.02]. Again, the low Kendall’s *W* statistics indicated variability related to the specific ranked order of the themes within stakeholder groups (**Figure 2**). Of note, policy makers ranked two themes notably different from all other stakeholder groups – ‘High-priority ‘patient-level’ & ‘economic’ outcomes for healthcare stakeholders’ (highest ranking theme) and ‘QEP integration into primary cancer care teams’ (lowest ranking theme).

**Table 3:** Final rankings & definitions of CPPI-defined research & KM themes.

Ranking	CPPI-Defined Research & KM Themes
1	**Title (*Theme E; R3*):** Integrating QEPs into primary cancer care teams
	**Goals:**
	**Research:** Establish the needs & define the role/scope of practice for QEPs within primary cancer care teams across geographical regions and healthcare settings **Research:** Evaluate the system-, team- & patient-level benefits, & cost-effectiveness of QEP inclusion within primary cancer care teams **Dissemination/Uptake/Implementation:** Promote the implementation of effective integration strategies for QEPs within primary cancer care teams
	**Stakeholders:** Administrators (healthcare institutions), community partners & practitioners, HCPs, policy makers, professional organizations & societies, QEPs, researchers, survivors and support persons, third-party healthcare insurers
	**Impacts:**
	Increased awareness & use of QEP expertise within primary cancer care teams Alleviated burden of exercise counselling from other primary cancer care team members Increased accessibility & quality of exercise-related patient education throughout the cancer trajectory Improved exercise screening & assessment leading to more appropriate patient triage & referrals & efficiency of use of medically supervised & community-based resources & support Improved short- & long-term patient outcomes leading to reduced healthcare costs & resource utilization
2	**Title (*Theme B; R3*):** Developing & promoting evidence-based exercise oncology education models for HCPs working with cancer survivors
	**Goals:**
	**Research/Dissemination:** Increase awareness & knowledge of HCPs on the benefits of exercise for cancer survivors through varying educational approaches **Research/Dissemination/Implementation:** Increase exercise-related communication between HCPs & survivors by developing, promoting, & embedding exercise communication resources & tools within medical & community care settings
	**Stakeholders:** Administrators (healthcare institutions), community partners & practitioners, educators, HCPs, policy makers, professional associations & societies, QEPs, researchers, survivors & support persons, unions
	**Impacts:**
	Increased HCPs’ motivation & proficiency to discuss the benefits, risk, & guidelines for exercise with survivors Increased rates of appropriate exercise endorsement (patient-level) & exercise program referrals (medically supervised- & community-based levels) by HCPs
3	**Title (*Theme G; R3*):** Improving accessibility of medically supervised & community-based cancer exercise support services for diverse groups of cancer survivors
	**Goals:**
	**Research:** Identify the exercise support needs, barriers, & preferred engagement strategies for (a) hard to reach & (b) understudied cancer populations globally **Research/Dissemination:** Leverage existing & establish novel infrastructure (physical & virtual) to create, evaluate, & promote accessible & equitable opportunities for survivors to engage with evidence-based cancer exercise support services **Uptake/Implementation:** Establish new & expand existing funding models to support survivors, QEPs, & community partners to increase accessibility of & equitable access to evidence-based cancer exercise support services
	**Stakeholders:** Administrators (healthcare institutions), community partners & practitioners, HCPs, policy makers, QEPs, researchers, survivors & support persons, third-party healthcare insurers
	**Impacts:**
	Increased awareness & understanding of the unique exercise-related support needs, barriers, & preferred engagement strategies of hard to reach & understudied cancer populations globally Improved behavioural & clinical outcomes *via* increased accessibility of appropriate evidence-based exercise support services for cancer survivors across the survivorship trajectory independent of geography, demographics & medical status Greater sustainability of accessible evidence-based exercise support services for all cancer survivors
4	**Title (*Theme F; R3*):** Establishing resources for referring cancer survivors between medical- & community-based cancer exercise services
	**Goals:**
	**Research:** Evaluate needs- & risk-based assessment & communication tools, with corresponding referral processes, to improve the appropriateness & efficiency of self- & practitioner-referrals between clinical & community cancer exercise services **Research/Dissemination:** Identify, describe, organize, & promote cancer exercise services within a region for self- & HCP referral **Dissemination/Uptake/Implementation:** Implement & support the use of appropriate resources (i.e. tools & systems) by HCPs & QEPs to improve communication between clinical & community-based cancer exercise services
	**Stakeholders:** Community partners & practitioners, industry, HCPs, QEPs, researchers, survivors and support persons
	**Impacts:**
	Increased awareness & appropriateness of self- & HCP-referrals of cancer survivors to cancer exercise services Established processes for efficient & appropriate referrals between clinical- & community-based cancer exercise services Improved communication between HCPs & QEPs in clinical- & community-based cancer exercise services leading to more effective case management
5	**Title (*Theme C; R3*):** Establishing exercise oncology training standards for QEPs across training environments
	**Goals:**
	**Research:** Develop education standards for (1) foundational & (2) continuing professional & community-based training opportunities in exercise oncology that are accessible to diverse QEPs (e.g. geographically, demographically, academically) **Dissemination:** Define & increase awareness of the boundaries & overlap of competencies/scope of practice for QEPs (i.e. allied health professionals ⟶ community practitioners) working in exercise oncology **Dissemination/Uptake/Implementation:** Promote & support the adoption of standards for evidence-based exercise oncology curriculums across training settings (e.g. community, professional association, college, undergraduate & graduate programs)
	**Stakeholders:** Administrators (healthcare institutions), community partners & practitioners, educators, HCPs, professional associations & societies, QEPs, researchers, survivors & support persons
	**Impacts:**
	Increased number of QEPs providing evidence-based exercise & rehabilitation support to survivors across medical, clinical & community settings Increased opportunities for QEPs to acquire appropriate knowledge & skills to support coordinated multidisciplinary exercise services (e.g., exercise counselling, screening, testing, & prescription) for cancer survivors Improved interprofessional communication & collaboration between QEPs to optimize care
6	**Title (*Theme D; R3*):** Enhancing technology-based strategies to improve the delivery of exercise support to demographically-, culturally-, & geographically-diverse communities of cancer survivors
	**Goals:**
	**Research:** Identify the technology-based needs of diverse cancer survivor populations to enable & improve all aspects of exercise support **Research:** Develop & evaluate the feasibility, safety, efficacy, effectiveness, & sustainability of technology-based exercise support strategies to meet the needs of diverse cancer survivors across the survivorship trajectory **Dissemination/Uptake/Implementation:** Promote & support increased opportunities for the integration of technologies within self-directed & supervised exercise support settings for diverse cancer survivor populations
	**Stakeholders:** Administrators (healthcare institutions), community partners & practitioners, HCPs, industry (e.g. technology companies), policy makers, QEPs, researchers, survivors & support persons, third-party healthcare insurers
	**Impacts:**
	Increased accessibility & awareness of feasible, safe, efficacious, effective, & sustainable technology-based exercise support systems & services for cancer survivors Decreased barriers to exercise engagement (e.g. time, cost, program proximity) for cancer survivors Increased reach of evidence-based, high-quality interventions for all cancer survivors in order to promote equity in exercise support across the survivorship trajectory Increased awareness & capacity of QEPs & community exercise programs to meet the needs of diverse cancer populations using technology
7	**Title (*Theme L; R3*):** Understanding the high-priority ‘patient-level’ & ‘economic’ outcomes for healthcare funders & decision-makers
	**Goals:**
	**Research/Dissemination:** Identify & promote the high-priority (1) patient outcomes & (2) health economic outcomes of healthcare funders & decision-makers (e.g. administrators & policy makers from healthcare institutes, third-party insurers, & government agencies) **Research:** Evaluate whether existing & emerging cancer exercise services can improve the identified high-priority outcomes of healthcare funders & decision-makers **Uptake/Implementation:** Optimize communication between healthcare stakeholders & (1) survivors & supporters, (2) QEPs, (3) HCPs, & (4) researchers to secure permanent policy & financial support for exercise as a standard of care in oncology
	**Stakeholders:** Administrators (healthcare institutions), community partners & practitioners, HCPs, policy makers, QEPs, researchers, survivors & support persons, third-party healthcare insurers
	**Impacts:**
	Increased awareness of, & research targeting, the high-priority (1) patient outcomes & (2) health economic outcomes of healthcare funders & decision-makers to inform public health policy & financial resource allocation towards supporting exercise as a standard of care in oncology High-quality evidence supporting the efficacy of medically-supervised & community-based exercise oncology support services to improve the identified high-priority patient & health economic outcomes Regular communication & collaboration between stakeholders to optimize the development & conduct of exercise oncology research that directly supports the establishment of permanent funding & infrastructure support for exercise as a standard of care in oncology
8	**Title (*Theme I; R3*):** Improving cancer survivor transitions across medically supervised, community-based, & self-directed exercise settings
	**Goals:**
	**Research:** Evaluate the feasibility & effectiveness of existing & new strategies to transition cancer survivors between exercise services within different settings (e.g. hospital-, community-, home-based) and with different formats (e.g. supervision levels, populations) **Research/Uptake/Implementation:** Evaluate, establish & implement a framework for improving risk & needs assessments, exercise education, behavioural support, & exercise testing & prescription across support settings for survivors in transition
	**Stakeholders:** Administrators (healthcare institutions), community partners & practitioners, HCPs, QEPs, researchers, survivors & support persons, third-party healthcare insurers
	**Impacts:**
	Improved survivor self-efficacy & engagement in sustained exercise behaviour while transitioning across exercise support settings & survivorship phases Increased self-efficacy & support for HCPs, QEPs, & community partners & practitioners in managing survivor transitions between various exercise support settings
9	**Title (*Theme H; R3*):** Developing & sharing of evidence-based resources to support academic & community partners in providing exercise services for cancer survivors
	**Goals:**
	**Research:** Identification of existing & development of new evidence-based resources to increase exercise engagement & exercise support for cancer survivors across geographic regions and support settings **Research/Uptake/Implementation:** Develop & sustain systems to share & recognize the developers of exercise resources that can be used directly by, or adapted to meet the needs of, academic & community partners to increase exercise engagement & exercise support for cancer survivors **Dissemination/Uptake:** Promote awareness & uptake of information sharing systems & use of appropriate resources by academic & community partners to increase exercise engagement & exercise support for cancer survivors
	**Stakeholders:** Administrators (healthcare institutions), community partners & practitioners, HCPs, QEPs, researchers, survivors & support persons
	**Impacts:**
	Improved content sharing of evidence-based resources across stakeholders to support survivor education, program development, & intervention delivery Increased research dissemination, impact & collaboration between academic & community partners Reduced redundancy, time & costs related to resource development across research, medically-supervised & community-based cancer exercise programs
10	**Title (*Theme K; R3*):** Optimizing approaches & resources to facilitate sustained exercise behaviour change in cancer survivors
	**Goals:**
	**Research/Dissemination:** Identify & promote existing & novel approaches to support sustained exercise behaviour change to meet the unique needs of individual, & groups of, cancer survivors **Research/Uptake/Implementation:** Explore & evaluate implementation strategies for existing & novel approaches to optimize sustained exercise behaviour change across different settings & populations of cancer survivors
	**Stakeholders:** Administrators (healthcare institutions), community partners & practitioners, HCPs, policy makers, QEPs, researchers, survivors & support persons, third-party healthcare insurers
	**Impacts:**
	Increased exercise self-efficacy & sustained exercise participation in cancer survivors *via* existing & novel exercise support approaches & resources Establishment of numerous effective exercise support strategies to meet the needs of diverse cancer survivor groups (e.g. demographic, cultural, geographic, behavioural) & promote survivors’ independence to self-manage their condition with exercise
11	**Title (*Theme A; R3*):** Enhancing communication strategies to increase cancer survivors’ exercise engagement throughout the survivorship trajectory
	**Goals:**
	1. **Research/Dissemination:** Establish & promote demographic-, cultural-, language- & region-specific survivor-identified communication content & approaches to: i. increase survivors’ & supporters’ awareness of exercise benefits, risks, and support services ii. motivate & support cancer survivors to change their exercise behaviour throughout the survivorship trajectory 2. **Research/Implementation:** Improve the quality of exercise communication for specific cancer survivor & supporter groups by developing & implementing recommendations for effective, evidence-based exercise communication content & approaches
	**Stakeholders:** Community partners, educators, HCPs, industry, QEPs, researchers, survivors & support persons
	**Impacts:**
	Improved survivor- & supporter-awareness of exercise benefits, risks & support services Improved HCP understanding of survivor & supporter-preferred exercise communication content & approaches to optimize exercise engagement & benefits for cancer survivors across the survivorship trajectory
12	**Title (*Theme J; R3*):** Establishing the appropriateness & benefits of community-based cancer exercise support services
	**Goals:**
	**Research:** Evaluate the appropriateness (feasibility, safety, tolerability) & benefits (efficacy & effectiveness (including cost)) of community-based exercise screening, testing, & intervention practices to optimize exercise-related risk management & benefits for diverse groups of cancer survivors **Research/Dissemination/Implementation:** Establish & promote minimum standards for community-level data collection & outcome reporting to meet the broad needs of exercise oncology stakeholders (i.e. survivors ⟶ policy makers) **Dissemination/Uptake/Implementation:** Promote the uptake & adoption of exercise support services that are appropriate & beneficial for diverse groups of cancer survivors with accompanying implementation strategies
	**Stakeholders:** Administrators (healthcare institutions), community partners & practitioners, HCPs, policy makers, QEPs, researchers, survivors & support persons, third-party healthcare insurers
	**Impacts:**
	Establishment of a robust evidence base supporting the appropriateness & benefits of community-based exercise interventions for cancer survivors to support the permanent adoption of exercise as a standard of cancer care Improved rigor of short- & long-term outcome assessments across various community settings to support the permanent adoption of exercise as a standard of cancer care Improved stakeholder knowledge surrounding the elements of cancer exercise program practice, design & delivery, & outcomes shown to be unsuccessful (e.g. not appropriate and/or beneficial) & successful (e.g. appropriate and/or beneficial) across diverse cancer survivor groups & community settings

## Discussion

This study leveraged the CPPI KM framework by engaging an international group of exercise oncology stakeholders to develop and refine a list of high-priority research and KM themes to support the implementation of exercise as a standard of cancer care. The results of the modified Delphi survey indicate there are 12 broad research and KM themes that are important to address to implement exercise support services for cancer survivors. The three most consistently high-ranking themes across stakeholder groups were: (1) QEP integration into primary cancer care teams; (2) Evidence-based exercise oncology education models for HCPs working with cancer survivors; and, (3) Accessibility of medically supervised & community-based cancer exercise programs & support services to meet the needs of diverse groups of cancer survivors. However, to our knowledge, this is the first study to demonstrate that the priorities for research and KM activities to support the implementation of exercise in cancer care are highly variable between and within stakeholder groups. This lack of consensus suggests there may not be an optimal rank or sequence for implementation-focused exercise oncology research and KM. However, the general agreement regarding the top- and bottom-ranked themes across stakeholder groups provides important guidance for future work.

The first objective was to identify a list of highly important exercise oncology research and KM themes. Themes related to QEP and HCP education, exercise support infrastructure, and survivor referrals were consistently rated among the most important across survey rounds. Of these, the theme ‘Developing and promoting evidence-based exercise oncology education models for HCPs working with cancer survivors’ had the highest importance ratings. Previous studies have suggested various approaches to educating HCPs on the benefits of exercise in oncology (e.g., education sessions, information sheets) (13, 24, 25). Nonetheless, the effects of these approaches have not been thoroughly examined and therefore their impact on the targeted outcomes (e.g., survivor referral rates, HCPs’ confidence to endorse and discuss exercise with survivors) are unknown. To address this theme, there is a need for research and KM activities aimed at developing effective approaches for exercise-related education for HCPs in foundational [e.g., undergraduate and professional degrees (15)] and continuing education settings. Recently, working groups like the ‘Moving Through Cancer Task Force’ (26) have launched research related to the ‘Establishing exercise oncology training standards for QEPs across training environments’ theme. There have also been success stories related to other highly rated themes (e.g., Integrating QEPs into primary cancer care teams) on local [e.g., ELLICSR, Princess Margaret Cancer Centre, Toronto (CA) (27); ActivOnco, Segal Cancer Centre, Montreal (CA) (28)], regional [e.g., ACE, Calgary (CA)] (29) and national (e.g., Australia (9)] levels. However, further research is needed to help understand which QEP integration strategies are most effective within specific settings and contexts. Overall, the high ratings of importance across all 12 of the final themes indicates there are multiple complementary and collaborative research and KM activities needed to support the implementation of exercise as a standard of cancer care.

The second priority for the study was to establish a ranked list of priorities for actionable research and KM to support the implementation of exercise as a standard component of cancer care. The low agreement regarding the specific theme rankings suggests there are elements of ‘importance’ to be addressed within all of the 12 themes *via* parallel lines of research and KM. However, the five top themes were consistently ranked among the most important. These findings provide strong preliminary support for the relative importance of these themes compared to the others. Comparing the theme rankings between stakeholder groups revealed the rankings for two themes were markedly different according to policy makers compared to the other stakeholder groups. Specifically, ‘Integrating QEPs into primary cancer care teams’ was among the highest ranked themes for all stakeholder groups except policy makers who ranked it the very lowest. Similarly, ‘Understanding the high-priority ‘patient-level’ & ‘economic’ outcomes for healthcare funders & decision-makers’ was the highest ranked theme for policy makers but was ranked moderate-to-low priority by all other stakeholder groups. These findings highlight potentially important differences in the perspectives and research priorities between policy makers and other stakeholder groups that may partially explain current challenges related to securing the funding and infrastructure needed to develop and sustain exercise support services for cancer survivors. Qualitative research aimed at understanding the differences in research priorities within and between stakeholder groups may help to understand the unique factors that impact research and KM priorities across stakeholders, sites, and healthcare systems. Findings from this project can be used to guide qualitative research aligned with advancing implementation of exercise into standard cancer care. However, in order to do this most effectively, KM initiatives aimed at improving communication and promoting a mutual understanding of the differences in priorities between and within stakeholder groups may ultimately be needed to facilitate the advancement of collaborative exercise implementation research in oncology and make more efficient use of the limited human, physical, and financial resources available to support it.

The findings of this study should be interpreted within the context of its limitations and implications. First, the study was focused on better defining and ranking priorities in exercise and cancer care and did not offer participants the opportunity to indicate they did not support the implementation of exercise as a standard of cancer care. As such, the findings may be biased to groups of stakeholders who hold positive beliefs on the integration of exercise; and, future research efforts are needed to balance null or negative attitudes towards exercise as standard of care. The noted inter-relatedness of the themes may have made it difficult for stakeholders to consider each theme in isolation. There is also likely some degree of personal and professional bias towards ranking certain themes above others between stakeholder groups that is even more difficult to account for given that 34% of the sample identified as belonging to more than one stakeholder group (e.g., QEPs advocating for integration). A better understanding of the differences in the priorities of stakeholders defined by stakeholder type(s) as well as other factors like age, gender, and country is needed and should be a focus of future research. We also acknowledge that there was an imbalance in stakeholder representation across the survey rounds that may impact the representativeness of our findings. Proportionally, the number of policy makers in the study may reflect their relative number within the community. However, it is likely that the opinions of policy makers may disproportionately influence whether exercise is implemented as a standard of cancer care. Therefore, at minimum, research and KM initiatives aimed at understanding the perspectives of policy makers will likely be important for achieving the ultimate goal of implementing exercise in oncology care. Finally, demographic data was not collected for the 17 policy makers who participated in Round 3 to address the aforementioned imbalance in stakeholder groups – resulting in their demographic data not being included in the descriptions of our sample. Notwithstanding these limitations, our finding that five themes consistently ranked higher than others has clear implications towards informing the scope of future work. Specifically, stakeholders can leverage our findings to develop and refine the scope of implementation-focused research activities and, when possible, immediately initiate KM activities towards supporting the implementation of exercise as a standard of cancer care. In particular, two of the top five ranked themes relating to HCP and QEP education (i.e., ‘Exercise oncology education models for oncologists & primary care teams’ and ‘Standardized exercise oncology training for diverse exercise professionals across various training environments’) are readily actionable for both KM and related implementation research.

## Conclusion

This study leveraged the CPPI KM framework and represents an important step towards establishing research and KM priorities to support the implementation of exercise as a standard of cancer care. Our findings can be used as a guide to inform the scope of related research and KM initiatives, as well as the funding opportunities available to support this work. Additional research is needed to better understand the differences in the proposed research priorities between and within stakeholder groups and, ultimately, facilitate the co-development of collaborative research and KM to support the implementation of exercise as a standard of cancer care.

## Data Availability Statement

The original contributions presented in the study are included in the article/**Supplementary Material**. Further inquiries can be directed to the corresponding author.

## Ethics Statement

The studies involving human participants were reviewed and approved by University of Toronto. The patients/participants provided their written informed consent to participate in this study.

## Author Contributions

Conception and design: SA, CS, and DSM. Study coordination and data processing: SA, and JST. Data acquisition, analysis, or interpretation: All authors. Drafting the article: SA. Reviewing the article: All authors. All authors contributed to the article and approved the submitted version.

## Funding

This study was funded by the Canadian Cancer Society (BC-RG-15-2 #316288).

## Conflict of Interest

The authors declare that the research was conducted in the absence of any commercial or financial relationships that could be construed as a potential conflict of interest.
