# Supplementary material for: The Exercise Oncology Knowledge Mobilization Initiative: An International Modified Delphi Study
Source: Front Oncol. 2021 Jul 19;11:713199. doi: 10.3389/fonc.2021.713199 (PMC8327176; doi:10.3389/fonc.2021.713199)
Supplement: Supplementary file 1 [file DataSheet_1.docx]

**The Co-Produced Pathway to Impact**


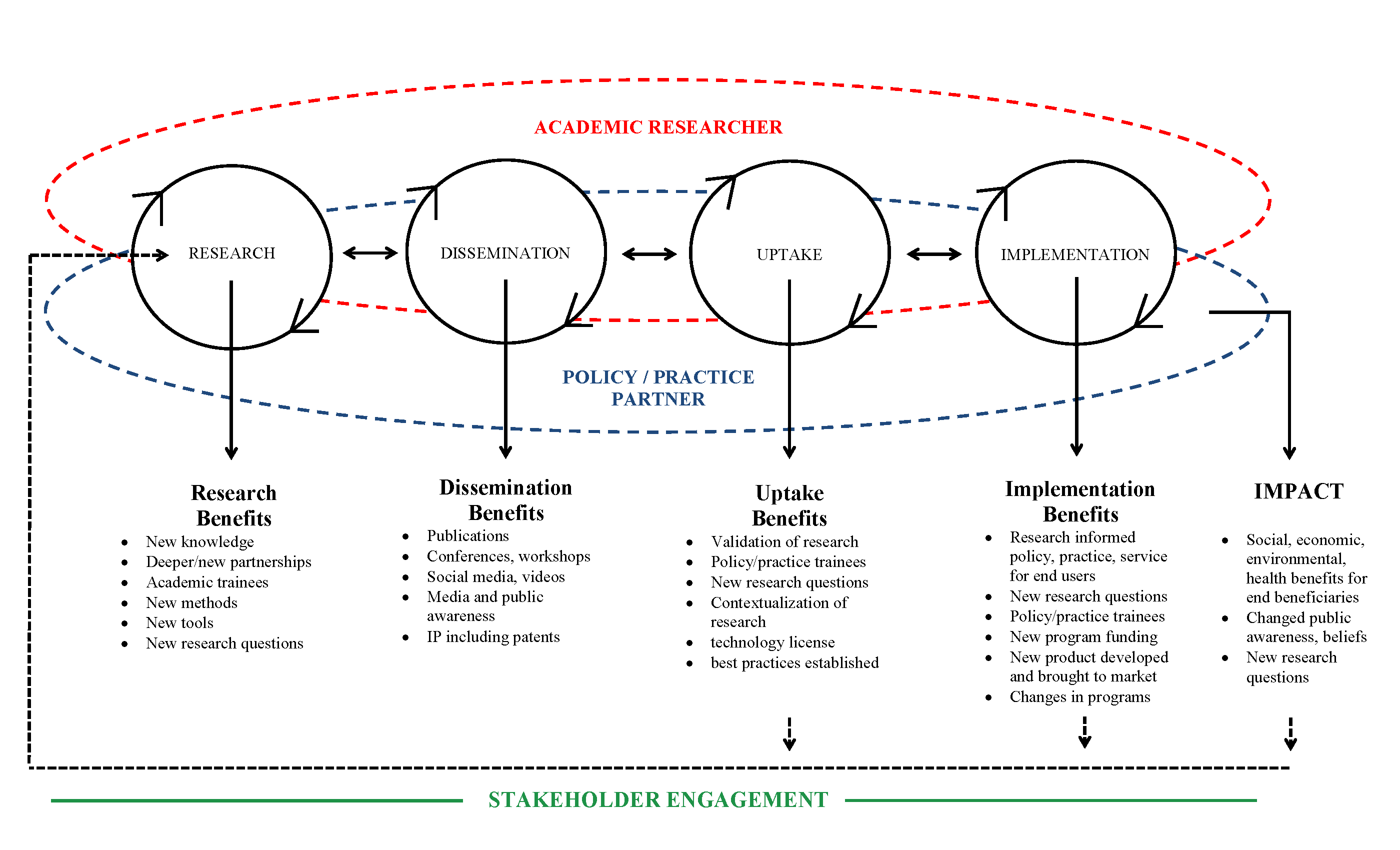


Adapted from Phipps et al., *JCES*, 2016

**Stakeholder Workshop: Theme Development and Responses**

Workshop themes are numbered to align with the themes presented in the round 1 survey. Un-numbered themes and individual responses were combined with numbered themes.

**Theme A (*from survey round 1*)**

| **CPPI Element** | **Responses** |
| --- | --- |
| Title | - Patient identified key messaging "stats" to create exercise adoption |
| Goal | - Determine patient-centred messaging for HCP's to convey to their patients to increase exercise engagement / behaviour change (do patient and health care provider priorities match?) |
| Stakeholders | - Patients/survivors - Clinicians - Social media marketing / advertising / communications professionals |
| Impact | - Patient will have clarity on goals / outcomes / feasibility of exercise participation and referral |
| **Additional Feedback** | |
| What worked | -- |
| Improvements | - In pathway, how to facilitate physical activity & work within logistics of the environment - Can you add something to trigger / prompt / remind oncologists to do this? |
| Questions | - How to measure effectiveness? - Would physicians be more flexible about messages delivered in material versus directly from the physician? |
| Ideas | - Use other stakeholders including conventional media / newspapers / radio / tv - Differentiate messaging for stage of treatment - Directing messaging to teachable moments - Differentiate messaging to patients through posters in office versus physician referral |

**Theme B (*from survey round 1*)**

| **CPPI Element** | **Responses** |
| --- | --- |
| Title | - Does an exercise intervention for oncology care providers improve attitudes and behaviours towards exercise referrals for related services |
| Goal | - Increase referrals/recommendations/engagement - Improve climate/culture/attitude about exercise for people with cancer - Improve oncologists, HCP |
| Stakeholders | - Oncology care providers - Admin - Wellness team - Unions and associations |
| Impact | - Increase physical activity/exercise in cancer patients - Improve symptom management, survival, QOL - Increase health of our clinical program |
| **Additional Feedback** | |
| What worked | - Culture of wellness/health will translate to better adherence by patients |
| Improvements | -- |
| Questions | - How much support needs to be provided to health care providers to do this? |
| Ideas | - Similar to "key messages" one for HCP and other education initiatives - Change environment - Make them par of the message - Mandate it - Could you also just study 'existing practices' (i.e., do HCP who exercise themselves deliver the message better? - Profiles of clinicians posted on website, hallways, marketing materials nonmedical and public - Have photos of clinicians exercise with what they think about exercise and benefits |

**Theme C (*from survey round 1*)**

| **CPPI Element** | **Responses** |
| --- | --- |
| Title | - Advancing availability and expertise of quality of exercise oncology specialists in the community |
| Goal | - Increase number and expertise of community-based exercise practitioners / specialists / fitness professionals / trainers - Barriers (cost for education, number of existing work force, reach and capacity of programs, certification issues, awareness of existing education programs) |
| Stakeholders | - Exercise specialists / fitness professionals / kin / physio - Oncology team (specialists, nurses, etc.) - Ex Onc Educators (researchers, senior EP's) - Community based fitness centres - Patients & family (carers) - Professional organizations |
| Impact | - Increase number of patients accessing high quality exercise in the community |
| **Additional Feedback** | |
| What worked | -- |
| Improvements | - (Consider) level of training and scope of practice - Go even further back to entry level university programs (PT schools) - Change improved? Financial plan, who pays? |
| Questions | - Acceptance for different certification bodies? - Who is providing exercise training programs in oncology? Are those programs recognized by education / professional bodies? |
| Ideas | - Create a standard of practice / education needed for "cancer exercise rehab specialist" (professional organizations be on board) - Put together the continued education programs at universities that have PT, Kin, OT and develop an exercise oncology training certificate program - Increase education and increase certification - Develop the program / certification for fitness professionals |

**Theme D (*from survey round 1*)**

| **CPPI Element** | **Responses** |
| --- | --- |
| Title | - Developing new approaches for reaching the hard to reach and understudied |
| Goal | - To increase knowledge of stakeholders in participatory approaches to intervention development for exercise oncology |
| Stakeholders | - Researchers - Patient partners - Policy organization - Community partners (CCS) / not for profits |
| Impact | - Better reach for hard to reach and understudied - Manual for stakeholders to do this work |
| **Additional Feedback** | |
| What worked | -- |
| Improvements | - Also from a patient perspective, what type of recruitment strategies would work to engage them in these studies? |
| Questions | - Why are the patients "hard to reach"? Geography? Likelihood to exercise? Health condition? |
| Ideas | - Introduce online programming and exercise modules - Focus on children and young people |

**Theme E (*from survey round 1*)**

| **CPPI Element** | **Responses** |
| --- | --- |
| Title | - Can exercise / physical activity engagement be increased with technology in rural / remote locations - Can technology be used to deliver an individually tailored exercise program in a group setting across geographic locations? |
| Goal | - Increase reach to high-quality programs - Decrease barriers -- financial - Increase education - Increase capacity (fitness professionals, programs) |
| Stakeholders | - Community partners - Clinical leaders - Existing exercise programs - Industry - Patients - Tech company - Feedback, usability - Fitness centres, community leaders |
| Impact | - Social connectedness - Decrease inequities (by geography) - Increase physical activity levels and health outcomes - Snowball effect -- health providers |
| **Additional Feedback** | |
| What worked | -- |
| Improvements | - Varied engagement due to: decreased social engagement, decreased accountability, decreased time management of members, decreased tech savvy |
| Questions | - What currently exists for tech in other health populations (e.g. "ECHO" for chronic pain)? - How will you create technology to be accessible for all (i.e. those who don't use it or have internet access, etc.? |
| Ideas | - Include sport as an option - Offering group classes using technology could increase adherence |

**Theme F (*from survey round 1*)**

| **CPPI Element** | **Responses** |
| --- | --- |
| Title | - Determining increased participation as an outcome of co-assessment and treatment |
| Goal | - Integrate exercise specialists (? Physician / nurse practitioners / physiatrist / ??) as part of the initial assessment team |
| Stakeholders | - Clinicians - Administration - Patient / survivors |
| Impact | - Every patient / survivor receives a meaningful referral to exercise / movement programming |
| **Additional Feedback** | |
| What worked | -- |
| Improvements | -- |
| Questions | -- |
| Ideas | -- |

**Theme G (*from survey round 1*)**

| **CPPI Element** | **Responses** |
| --- | --- |
| Title | - Develop a simplified tool for oncology providers (MD, Nurse, PT, OT, GP) for referral (who, where, when) |
| Goal | - Clinician team don't know about existing programs in hospital or community - Are clinicians really convinced of necessity/benefits of exercise? - As much as we know exercise is good, the research doesn't actually prove that its safe for practitioners to say, "go forth into the community" (i.e., our data is in clinically supervised settings (RCT vs pragmatic) |
| Stakeholders | - QEP - PT - GP - Oncologists - Patients - Physiatrist - Cardiologist - Nurses |
| Impact | - Increase the numbers of people exercising and the number of patients referred |
| **Additional Feedback** | |
| What worked | - Do it - use the Pathways model (Santa Mina et al) |
| Improvements | - Look to literature on changing HCP behaviour from other fields |
| Questions | - Which HCP - MD, RN, PT, QEP |
| Ideas | - Add patients as stakeholders - patients are teachers of program at UofT - Might need incentive such as CECs because they are very busy and lack of time to counsel on everything |

**Theme H (*from survey round 1*)**

| **CPPI Element** | **Responses** |
| --- | --- |
| Title | - Accessible movement for all |
| Goal | - Increase accessibility to movement programming for all cancer patients and survivors |
| Stakeholders | - Patients/survivors - Clinicians - Administrators (institution) - Knowledge brokers - Community partners |
| Impact | - Opportunity to access these programs across the lifespan and cancer trajectory |
| **Additional Feedback** | |
| What worked | -- |
| Improvements | - Tailor to underserved populations |
| Questions | - Geographic access or access to most appropriate program for patients? |
| Ideas | - Start exercise where patient is at 🡪 quick win and move them up - Government engagement and lobbying - what health outcomes will help government engage? |

**Theme I (*from survey round 1*)**

| **CPPI Element** | **Responses** |
| --- | --- |
| Title | - Repository of Oncology Exercise Resources (ROER) |
| Goal | - Improve content sharing, proven effective interventions & resources, - Increase collaboration - Increase reach and impact |
| Stakeholders | - Researchers - Clinicians - Academics - Patients - Community partners |
| Impact | - Accelerated impact - Reduced time and costs - Improve education - Implementation - Acknowledgement |
| **Additional Feedback** | |
| What worked | -- |
| Improvements | - Have a conference or workshop to disseminate shared resources so there is dedicated time - Too many things to follow - can we do a group or flag on Research Gate? |
| Questions | -- |
| Ideas | - NIH - look at funding - RTIPS repository - Not specific to oncology - Use a social media strategy to raise awareness of resources - International repository |

**Theme J (*from survey round 1*)**

| **CPPI Element** | **Responses** |
| --- | --- |
| Title | - Transition, uptake and maintenance of patients moving to exercise in the community |
| Goal | - Successfully transition from hospital-based program to community programs - How do you transition? - How do you incentivize? - Fear of being on their own cost? - Loss of support of other survivors |
| Stakeholders | - Patients - Hospital-based exercise facilitators - Community partners |
| Impact | - Reduced risk of recurrence, Reduced side effects - Decreased cost to healthcare system |
| **Additional Feedback** | |
| What worked | - Decreased barriers to exercise |
| Improvements | - Government engagement |
| Questions | - When to transition? - How to know who/when? - How to evaluate? - What are other relevant outcome measures? |
| Ideas | - Programs need to emphasize transition to patient independence - Are there standards required in order to recommend transition programs? - Provide education for patients about the benefits as an incentive - Direct referral process to community partners - Partner with gyms, health clubs that can offer discounted memberships for cancer survivors - Community also has problems moving participants from "community programs" to be more independent and more self-sufficient |

**Theme K (*from survey round 1*)**

| **CPPI Element** | **Responses** |
| --- | --- |
| Title | - Building community capacity |
| Goal | - Database of evidence-based models - Standardized reporting and collection (effectiveness / intervention) - Cost-effectiveness - Standardized training |
| Stakeholders | - Community partners - Academics - Practitioners (exercise professionals / physicians) |
| Impact | - Increased capacity for exercise referral --> community |
| **Additional Feedback** | |
| What worked | - Ability to look at long term outcomes |
| Improvements | - Develop certification program to enhance skills |
| Questions | - Who operates this or is the best to organize / manage? - Consider efficiency versus effectiveness |
| Ideas | -- |

**Theme L (*from survey round 1*)**

Developed to address a multitude of unaddressed individual items from workshop participants (see below – Individual Response Categories).

**Theme M (*from survey round 1*)**

| **CPPI Element** | **Responses** |
| --- | --- |
| Title | - Translating different models of care to different populations & settings |
| Goal | - Can other models of care (e.g. Livestrong / cardiac rehab) be integrated into cancer care systems? - How can we best support cancer patients to be independent and self-manage? (role of digital technology / peer support and volunteer programs); use chronic disease self-management program |
| Stakeholders | - Hospital administration - Re-imbursement systems - Patient groups - Community groups |
| Impact | - Sustainable system of care 🡪 better patient outcomes |
| **Additional Feedback** | |
| What worked | - Integrated current models - Using existing model of care will have wider reach since they have capacity to be successful |
| Improvements | - Lay terms impact of patient outcomes - Emphasize cancer patients self-managing - Independence rather than dependence |
| Questions | -- |
| Ideas | - E.g. British Columbia online health link |

**Theme N (*from survey round 1*)**

| **CPPI Element** | **Responses** |
| --- | --- |
| Title | - Understanding the economic argument for exercise programming as part of cancer care: A multi-sector scoping review |
| Goal | - Understand economic priorities of stakeholders and undertake costing project |
| Stakeholders | - Provincial health authorities - Extended health care providers (e.g., insurance) - RTW/LTD - Individuals with cancer (out of pocket) - Health care administrators - Not-for-profit community partners |
| Impact | - Economic case studies / economist formatted language - Whitepapers/briefing notes for advocacy |
| **Additional Feedback** | |
| What worked | -- |
| Improvements | - Health economist role as a key player |
| Questions | -- |
| Ideas | - Consider including mortality data - Include patients as stakeholders, they can tell you the impact on QOL and health of not using exercise covered - Consider determining/focusing on what health outcome data matters for patients, administrators, policy makers |

**Additional Themes (*integrated into other themes*)**

| **CPPI Element** | **Responses** |
| --- | --- |
| Title | - Commit to care |
| Goal | - Engage physicians and health policy administrators in determining path to standard of care (list / guidelines) in practice - What will it take to do this? |
| Stakeholders | - Physicians - Health Policy Admin - Institutional - Government (provincial / federal) |
| Impact | - Research agendas identified - Securing Funding |
| **Additional Feedback** | |
| What worked | -- |
| Improvements | - Need to define which physicians |
| Questions | - Barriers? What do you need to run the program? - What do you need to commit (engage with policy makers) |
| Ideas | - Tailor messages and material to the needs of HCPs so that they will use it |

| **CPPI Element** | **Responses** |
| --- | --- |
| Title | - Maximizing education within stakeholders |
| Goal | - Patient awareness of benefits of exercise - Motivation / confidence to refer by physician / HCP's - Exercise professionals well trained to work with people with cancer - Quality framework in education (curriculum and evaluation; knowledge, skills) |
| Stakeholders | - Patients - HCP's - Exercise professionals |
| Impact | - Increased trained professionals - Better system to accept people with cancer |
| **Additional Feedback** | |
| What worked | -- |
| Improvements | -- |
| Questions | -- |
| Ideas | - (Involve?) CCO funder of cancer - (Use?) education to maximize stakeholder roles (system) - Educate HCP / fitness / pts |

| **CPPI Element** | **Responses** |
| --- | --- |
| Title | - Addressing health-care providers knowledge and skill gap |
| Goal | - Translating exercise oncology evidence base to health professionals - Develop tools to assist HCP's to advocate, promote, and monitor patient physical activity |
| Stakeholders | - Health care provider - Regulatory bodies - Higher education establishments (medical school / university) |
| Impact | - Translation of the clinical guidelines (every health professional providing exercise advice to every patient) |
| **Additional Feedback** | |
| What worked | -- |
| Improvements | -- |
| Questions | -- |
| Ideas | -- |

**Individual Response Categories (*133 additional responses*)**

**Education:**

- Does the introduction of an institution specific PR campaign on PA result in increased PA knowledge & behaviour?
- What type of Pt campaigns work to build awareness for the benefits of PA?
- Increase knowledge/awareness of importance/benefits/safety of exercise in cancer recovery for physicians and patients
- Knowledge gap - standardized professional training
- Increase referrals if have trained
- Educate HCPs to talk about exercise as a real therapy amplifier not alternative therapy
- How do we increase the number of qualifications of community-based exercise specialists?
- Standardized training for exercise oncology practitioners
- What is needed to include exercise in oncology as a topic in the curriculum of medical, physio, KIN…programs?
- Train more QEPs
- Normalize or de-normalize exercise? Like smoking messaging with photos
- Knowledge translation of guidelines
- Oncologist training, are they best to refer, do they know?
- How to motivate oncologist and other doctors to start prescribing exercise as part of cancer treatment?
- Mobile RV disseminating exercise guidance? Quality of exercise (not just guidelines) - the do's and don'ts
- Exercise oncology resources & standards of certification
- Physician training on exercise in medical school, residency, represented in exams (aid in physician comfort)
- Increase KHCPs PA levels that might influence their own prescribing habits - more active HCPs may lead to increase PA discussion with patients - worksite interventions
- Increase knowledge of HCPs both within and outside rehab of CA related education/information for them to implement into practice or be able to transfer knowledge to patient re: recommendations
- Education to community partners
- Develop a staged approach to intervention in the office, teachable moments, dx to during Tx to after Tx
- Increase provider knowledge in non-breast clinics about risk of lymphedema and benefits of exercise
- Regular education forum for community leaders - latest advances, stepwise options
- HCPs & providers gap between knowledge, enquiry, and provision
- Focus on HCP counselling habits. What will convince them to prescribe exercise? Increase knowledge; survival outcome evidence needed; referral to exercise programming
- Take knowledge that we have from research & generate simple discussion cards for oncologists to counsel if there is a lack of knowledge
- Widespread education models in healthcare

**Evidence**

- Standardize reporting of exercise interventions in clinical trials
- Intervention for sedentary surgical patients to encourage small behaviour changes to increase activity
- FITT prescription & behaviour change
- Metastatic exercise inclusion and exclusion criteria
- Great need for pragmatic trials
- Great need to document safety profile of exercise

**Resources**

- Diversity of resources in a changing landscape
- Pool resources to help everyone involved (pt., students, community, medical) - passive vs active dissemination
- Education of effective / successful models of exercise in different settings
- Resource sharing repository

**Care Models & Clinical Support**

- Engage patients in a one-hour assessment with referrals & recommendations and determine impact
- Applying different models of care in different countries & healthcare systems
- Adding exercise to survivorship care plans
- Incorporate cancer exercise specialist as a part of supportive care / treatment team to assess change in patient trajectory & provide exercise recommendations
- Implement prehab at various stages of cancer continuum
- Gap: risk management; goal: identify high vs low risk patients from cancer treatment or exercise perspective
- Help clinicians screen and triage - conversation reminder - embed expertise in teams

**Funding**

- Exercise programs for cancer patients - who pays for them?
- Funding based on cancer centre evaluation - $ for implementation
- Advocacy for reimbursement for services (provincial health authorities / extended health / WCB)
- Secure funding for exercise as a standard part of cancer recovery pathway (from policy makers/administrators)

**Transitions**

- Transition, uptake, and maintain in the community
- Bridge the gap between hospital and community programs

**Referral Mechanisms**

- Provide pathway to pts-allow them to navigate or self-refer themselves
- Clinical to community - system referrals - HCP roles - QEP roles
- Indicators or algorithms for referral to various "level" of service - who needs what?
- Referral strategies
- Standardized direct referral mechanisms
- Physical evaluation based on participation/collaboration/implementation team i.e., physician who don't refer have decreased evaluations
- Physiotherapy care prescriptions
- Make it easier for physicians to refer to PT
- When - should ex be implemented? Who - should refer to ex? How - should referral happen?
- Simplified tool for oncologists
- Tool for oncologists (contextualised with local program)
- How do we incentivize clinicians to Rx exercise to patients?
- Resources for clinician referrals
- Referrals/EMR

**Technology Use**

- Use of telehealth or other technologies
- Develop a Wii / video game that promotes / leads patients through exercise
- Increase use of tech "exergaming"
- Exergaming
- Data linkages
- Online access to exercise and support to do at home (business streaming or using videos for lunchtime fitness)
- Role of technology and digital platforms in supporting cancer patients to exercise safely in the community (self-management and independence)

**Interprofessional Trust & Communication**

- "Oncologist portal": a way that researchers could get 2 cents from oncologists to make sure there is clinical utility / tailor projects (i.e. prompt project click 4/6 issue)
- Exercise in EMR
- Problem: Trust that patients will get sent back if problems result
- Problem: Oncologist uptake for rehab interest (timing, relevance, and trust)
- Including ALL members of health care team (oncologist, RN, Patients)
- How to foster communication and partnerships among organizations that promote exercise for cancer survivors, so that timely and meaningful interventions are offered?

**Sustain Behaviour Change**

- Lifelong exercise behaviour change component (habit); affordable ongoing programming
- What are the long-term effects of exercise / how does exercise alter the aging trajectory beyond 6 months?
- Patient motivation when someone sitting in front of me (pills, therapies, cost of therapy, location)
- Increase research in behaviour change model of exercise adaption
- Incentives for patients to get off the couch (reduced cost, friend gains access)

**Outcomes**

- What data do policy makers want to be able to make decisions on funding for cancer exercise professionals?
- Determine perceived barriers per physicians
- How to motivate the unmotivated
- Can we change the culture of cancer centres to include wellness as a component of improved outcomes?
- How do you help and support physicians in actively monitor and advocate and measure treatment compliance?
- Determinants of adherence
- Characteristics of those that adhere versus non-adhere
- Stakeholders economic priorities
- Costing projects in Canada; what does physical activity during treatment reduce cost (chemo, radiation, hormonal); cost effectiveness; cost benefits
- Long term outcomes (what is needed?) > 6 months
- Standardized reporting of outcomes (building community capacity)
- What additional outcomes are needed to drive change?
- Establishing cost-effectiveness for insurance company or public / government funding
- What is the relationship between physician / nurse physical activity levels for guidelines adherent care?

**Community Programs**

- Review compromises made in cardiac rehabilitation community
- Gym in cancer centre
- Problem: lack of community resources or knowledge of these resources
- Peer support volunteer led program (trained navigator)
- More than just a place to access personal activity (support groups; information about the disease)
- Ways for community organizations to participate in research (networking opportunities)
- Knowledge gaps: capacity in community (how to deliver and where)
- Sports leagues (low level)
- As part of chemotherapy treatment have facilities at hospitals or treatment centres for patients to go as pre-treatment
- Support groups, well spring, iLymphoma, etc. Gilda's club; have exercise as a part of these programs
- YMCA or Goodlife incentives (this existed at one time)
- How to integrate the cardiac rehab model into the cancer community (support and reimbursement)
- How to embed PA programs into current clinical format (like cardiac rehab) --> treatment to known entity
- PA programming within hospital; disseminate the program let the clinician know
- Special gym spaces and programs
- Peer led programs (volunteers); outreach to local spaces small group can meet close to home
- Cardiac rehab model; duplication in exercise oncology
- Patient preferred activities similar interest activities
- Link what people like and know and what they need (bootcamp, etc.)
- Peer mentoring
- Implement buddy system for motivation, support, accountability

**Access**

- Gap: how do we pay for services?
- Populations in need; triaging, prioritizing
- How do we reach rural populations?
- Applying current knowledge to rare and understudied
- Delivering exercise programmes for hard to reach (ethnic minorities / low SES) cancer groups
- How can we facilitate having exercise programs for cancer survivors to be free of change / covered by insurances or provincial funds?
- Increase coverage / access for 'return to work' services for cancer survivors (how do we get cancer rehab funded?)
- Reach
- Increase accessibility to services for individuals with cancer during active treatments within the institution
- How to expand the reach of existing programs / services (geographically, SES / health inequities / health status)
- Increase access / engagement to cancer rehab services for hard to reach populations
- How to deliver exercise to individuals in rural / remote locations

**Other**

- Determine thresholds within the spectrum of 'lesser' models of physical activity
- Projects needed to focus on how HCPs can deliver PA info and programs to cancer survivors (focus on barriers / facilitators, having tangible programs that oncologists will use based on what would help them)
- Can the implementation of an exercise treatment (or other tool) increase guideline compliant behaviour for oncology professionals and patients?
- Cancer Culture: how to stop 'cancerism' among oncology professionals

**Survey Round 1 – Open-ended Stakeholder Feedback**

| **Round 1 Theme A** | **Details** |
| --- | --- |
| **Title** | messaging seems to be a subset of increasing AWARENESS. I might prefer that term. |
|  | Not entirely certain I understand the term messaging. Does this mean communication? |
|  | The title, the aim appears to be to recognize and better address survivors’ priorities are around engaging in exercise to better support them by addressing communication but I'm not sure the title reflects this |
| **Goals** | Add to goals: establish pragmatic pathways to help survivors identify appropriate exercise stepped care services (tie into value-based care metrics) |
|  | messaging priorities - gives the impression that communication could only be in the form of text - but lots of other modes of communication and social media platforms exist? consider "communication platforms" instead |
|  | Identify multiple time points along the cancer trajectory that messages (tailored) should be delivered. Not just one point. |
|  | Consider co-development process - i.e., start with survivor education then work with them to identify the priorities. Also consider identifying optimal communication method/approach (e.g., in person, print, web). |
|  | Under goals, clarify what "messaging strategies are". |
|  | For behaviour change, we will need an overarching framework. So, we need to know what framework(s) works best in particular contexts. The framework then helps us to identify "targets" for these messages, e.g., self-efficacy, autonomous motivation, cost-benefits, etc. |
|  | I'm unsure about what "survivor identified messaging" means... |
|  | I would add that messaging should be with respect to increasing engagement with exercise-related behaviour (dose) that has evidence for efficacy for outcomes of interest |
|  | To be sure that messaging is inclusive to all levels of education, language and socioeconomic status. Messaging delivered through multiple methods. Inclusivity to all. |
|  | Does this include messaging strategies for family members and friends of survivors and support if engagement with exercise affects these relationships? |
|  | Do we know if these techniques work for behaviour change? And who delivering these messages are best to do so that can elicit the best outcome |
| **Stakeholders** | Researchers could benefit greatly from collaborating with media/communication experts to develop resources that are engaging, of high production quality and are put out to the appropriate outlets to maximise exposure. |
|  | Further, I would add NPOs to the stakeholders, like COSA, ASCO, ACSM |
|  | Identify specific groups of physicians as being stakeholders (oncologists, general practitioners, etc. |
|  | consider including health behavior experts, technology experts, and marketing experts |
|  | Just consistency of terminology used for naming stakeholders - Qualified Exercise and rehabilitation professionals - to keep consistent with Q11 |
|  | Would include professional organizations and university educators as stakeholders -- our QEP need to have access to up-to-date evidence-based practices and education to provide clear, consistent messaging |
|  | What type of "researchers" do you mean? |
| **Impacts** | In impacts I would change the wording from survivor preferences to survivor needs |
|  | Messaging strategies and preferences are very different from effective counselling by HCP; to me these are two very different lines of inquiry |
| **General** | Better differentiate physical activity and exercise here. Maybe it should be "participation in physical activity and exercise". |
|  | define what a survivor means here and in the whole project - is this clearly people with no metastatic disease. if so, great. if not, then this type of information will need to be very different for each type of person |
|  | look at motivators for the survivors e.g. enhanced QoL, survival and target messages to that |
|  | Rename/reword to indicate more clearly that messaging is to patients/survivors (rather than physicians, caregivers, general public) if that is what was intended |
|  | The way messaging is delivered to individuals with cancer is insufficient. The content and method of delivery could be improved to be more engaging. |
|  | It is important to clarify how you want to develop this strategy (web-based, mobile app, text message...) |
|  | It seems like the strategies would have to be country/region specific. It might help to clarify that. |
|  | Improve survivor compliance, that's the real issue |
|  | I think we also need to improve the survivors understanding of the importance of exercise as well. |
|  | as above, that unclear definition of survivor can make messaging difficult, and potentially inaccurate. For example, on the moving through medicine website the handouts state that exercise can improve overall survival - this has not been definitely proven. if "survivor" is defined by the IOM definition, then even somebody with metastatic disease is included and the messaging is not clear, nor inclusive. and, the safety and information about exercise will need to be different. |
|  | in which languages would the information be provided? Please use a literacy level 4 when writing messages, use visual cues |
|  | I'm not sure what this means. Is this figurative or literal? How you get in contact with the patients (phone, text, email), or is it how you get across messages/transfer knowledge? |
|  | What type of messaging medium will be used? Will these be in-person, by telephone, via text messaging? Will this include access to a discussion forum where patients can motivate themselves? Who will provide these messages to patients? |
|  | Interested to know what the platforms for messaging could be (i.e., in-person delivery, online) |
|  | Motivation being on a continuum, no matter the prompts, if an individual does not want to change, will constant messaging provide a significant impact? As much as contact with someone who cares for their wellbeing? |
|  | By messaging are you meaning contacting, communicating? |
|  | Be more clear with the phrasing? |
|  | I am sure this will come out in the messaging, but what isn't clear in the above is that this would be appropriate for all cancer survivors (and that survivor is any point after diagnosis). In its current format, it may be confused with addressing only post-treatment survivors. |
|  | It is important to clarify how you want to develop this strategy (web-based, mobile app, text message...) |
|  | The goals seem very broad. What kind of 'messaging'? How is a 'cancer survivor' going to be defined? Will participants have options for exercise paradigm - ensuring the right program for each individual? |
|  | My concerns reside with dealing the effects of chemotherapy. Seeing the acute and chronic effects of chemotherapy, I have a different understanding from most researchers regarding the feasibility of exercise for survivors. I think there needs to be more specifics regarding types of exercise. |
|  | No, this is the crux to much of the problem, if people aren't approached in a timely and appropriate manner, they are unlikely to be open to engagement. Timely and clear communication is vital |
|  | Would the best message reinforce the benefits of exercising with cancer or express the potential concerns of not exercising with cancer? |
|  | There may be a different in practitioner roles and related messages...particularly as it relates to patients prior to treatment, on treatment or post treatment. |
|  | What type of messaging medium will be used? Will these be in-person, by telephone, via text messaging? Will this include access to a discussion forum where patients can motivate themselves? Who will provide these messages to patients? |
|  | I'm not 100% clear of what we mean by "messaging priorities" The term is not clear to me. I'm guessing it relates to ways of communication. |
| **Not Actionable** | always room to improve |
|  | Not done enough |
|  | Not clear to me how this messaging relates to individuals performing exercise training |
|  | understanding of the physiology of return to exercise and also understanding the role of planned rest so as to not boom and bust |
|  | Exercise works! |
|  | See above |
|  | as previous question |
|  | I'm not sure what the question here was |
|  | an overview list in advance of the 14 priorities would have been good - working a bit blind here. but that's ok. |
|  | It's super broad but another good start to chip away at practitioner resistance and/or willful ignorance or, hopefully, work against how busy practitioners are, just scrambling to get pts seen and push them through the standardized process |
| **Different Theme** | How to engage care providers that do not exercise regularly to support patients who should be exercising |
|  | Ensure doctors are more aware of the importance of exercise in cancer survivorship |
|  | the medical team and the exercise rehab staff need to be singing of the same hymn sheet. i.e. if the patient hears the same message from both parties, they are more likely to understand the importance. perhaps a training day including both teams would improve such a working relationship. Also regular updates back to medical team is required to keep the patient engaged |
|  | Add a goal: provide help to find qualified training facilities. |
|  | I think helping survivors to understand the differences between PA and exercise and connecting them to local resources outside of a cancer center is critical to support engagement. Another big part of this is patient-centered prospective referrals. |
| **Round 1 Theme B** | **Details** |
| **Title** | Title may be too broad and needs to include clinicians outside of oncologist and PCP |
| **Goals** | In goal 1 add in attitudes and beliefs of oncologists to make more specific |
|  | maybe adding onto goal 1 - '... and best approach to referral i.e. exercise professionals' |
|  | I would add method to research goals. What would be the most effective method to increase awareness among oncologists? Would it be training, leaflet, new service provision model, different consultation model? I would maybe add changing medical culture as well. A cultural change is needed at some places. |
|  | The timing of education for Oncologists might be a consideration. I think it's worth exploring how we can both pitch to current practitioners and engage in University education to integrate modules into medical school training. I think ultimately, having a multi-pronged approach and priming them as often as we can in as different ways as possible |
|  | I see Goal #2 as MUCH higher importance and need than Goal #1 in this priority, not sure they should be treated equally. I believe that most physicians are aware of benefits, they just don't have the knowledge to communicate that well enough. |
|  | Another important research goal is how these educational strategies can be implemented in a way that is feasible given provider workload and burnout. |
|  | Having the universities include QEP to educate future oncology providers and PCPs about exercise for cancer survivors |
|  | Increase awareness on the need for advancements in inter-professional education and practice in cancer care |
|  | The title includes "tools and resources", but right now I think that the resources component is somewhat lacking from the goals. To me, a useful third goal would be "To increase the resources and tools available to Oncologists to educate patients about exercise and refer to exercise services". We definitely need goals #1 and #2, but alone they are not enough as one of the underlying issues is that Oncologists don't have time, therefore they need easy resources to use (this would be the low hanging fruit in my opinion). |
|  | I'm not aware of any literature that states that oncologists have low motivation, low attitudes, or lack of belief that exercise is important when it comes to exercise counseling/referral or that they don't "endorse it." The literature suggests that it's potentially an "intention-behaviour" gap - i.e. the intention is there but the tools to put it into action are not. The evidence suggests that clinicians lack awareness/knowledge of guidelines, knowledge of program availability, and lack of knowledge on HOW to counsel, rather than a belief that this shouldn't be a part of care. I would suggest re-wording most of this to reflect (a) the specific action that should be targeted (? something like "increasing provider discussion & referral of exercise" rather than changing attitudes or beliefs) and focusing on the barriers reflected in the literature related to this action. |
|  | Increase learning in exercise during university and medical school |
|  | Not sure whether you'll be able to change their 'capacity' to effectively educate patients (given their constant claim regarding time constraints), but you could change their 'competence and confidence' |
|  | Include exercise professionals to show practitioners what they do. |
|  | Not focus on Oncologists, but rather whole team, e.g., case managers as part of the oncological team etc. |
|  | Increased oncologists’ motivation & proficiency in exercise counselling; this may not be the right goal. They need to recommend and refer but I do not think it is an effective use of their time to counsel on exercise when there are other members of the healthcare time who have this expertise and skill already. Refer. |
|  | Consider adding goal of co-developing the exercise communication and referral tools with HCPs - optimize usability. |
|  | Remove the expectation for oncologists to counsel on exercise and instead equip them with easy referral to QEPs |
|  | Goals: exercise related knowledge rather than competence. 2. capacity to direct patients to the appropriate resources. |
|  | There are barriers that prevent HCP i.e. physicians to make these referrals. If it's not in their workflow it doesn't happen. Also - targeting nurses may be a better navigator for referrals and education for patients. |
|  | Consider changing or separating goals 1 and 2 |
|  | As per comment above; upskilling oncologists to counsel on exercise does not seem like the ideal goal |
|  | I think I’m the UK the oncologists will report they have very little time to do this and therefore it doesn’t just need to be the oncologist but the whole team that is involved |
|  | This is crucially important and involves more than just making providers aware of the data re: exercise. Providing tools & time for HCPs to engage in education and promotion of physical activity are the most important things we can do. |
|  | When will this education be taking place? |
|  | I might consider focusing this education more on my primary care team, family doctor rather than my oncology team as once my treatment finished. I've had very little oversight from them except for my 3x a year and now yearly check-ups. I'd prefer the health care professionals I see on a more regular basis to understand and encourage me in this area. |
|  | Improve oncologists’ ability to articulate the exercise benefits for cancer patients and relate that to individual's QoL |
|  | more education to encourage earlier engagement to therapy |
|  | As noted above, based on our experiences here, our focus with the oncologists is education/referral to appropriate supportive resources. |
|  | training needs to have some reward in order to not just get those interested already and be able to fit in their very busy work lives |
| **Stakeholders** | Given the title, under goals and impacts you need to add PCP (not just oncologists). |
|  | I would consider adding policy personnel to the list of stakeholders. Changing workflow like this may need "buy in" from those even higher than administration level. |
|  | The mention of parents/care givers. This is really important with younger patients. E.g. Increase awareness & improve attitudes & beliefs towards the benefits of exercise for cancer survivors AND PARENTS/CARERS |
|  | should this include kinesiologists in addition to oncologists? It is unsure whether the oncologist should be the one to provide information on exercise to the patient |
|  | Also include specialist nurses in oncologist training |
|  | This is not only oncologists but should also include surgeons and oncology mid-level providers. |
|  | It’s not just the oncologist and primary care team--some patients see a different provider for their "primary" care like a urologist and have no other clinician |
|  | Both physicians and oncologists are identified as stake holders. Which is it or more importantly which of the two labels should be used |
|  | include advanced practice nurses, RNs, etc. if not included under "HCP"; also need implementation science experts who know how to change provider behavior with electronic health record interventions |
|  | More emphasis on the supportive care teams e.g. nurses. The priorities of an oncologist do not necessarily include an exercise referral. It should be highlighted or the questioned should be asked during a consultation with a nurse. |
|  | I would think that exercise professionals would also be stakeholders in this to ensure the GPs are receiving accurate information? |
| **Impacts** | interested in learn more about the impact on institutional culture of health/wellness |
|  | Under IMPACT: disagree with #2 physicians should be counselling patients to participate but not prescribe exercises in addition to all their other responsibilities. #2 increased oncologists’ motivation to refer and have knowledge of exercise risks and benefits |
| **General** | For the cancer treatment team, need to include evidence that exercise has significant benefits for patients in a domain of interest to oncologists (e.g., overall survival, tolerance to treatment, reduced risk of recurrence, etc.) |
|  | The focus on oncologists as endorsers of exercise is appropriate. I do not believe, based on work looking at oncologists’ role in tobacco screening and cessation counselling that the oncologists will counsel directly. |
|  | going as far back as medical school training to emphasize the importance of lifestyle behaviors for health |
|  | Focus on more than just the oncologists (e.g. GP, NP and surgeons) especially if we are talking about behavior change of survivors. I know it mentioned primary care providers, but the focus seems to be on oncologists. |
|  | My oncologists encouraged exercise but some nurses, OTs, etc. sort of told me to take it easy or were uncomfortable answering my Q’s - example - how soon can I try jogging after lumpectomy? I had to figure it out on my own. |
|  | perhaps just that oncologists I know are swamped, of course, and most often they only want to encourage the activity yet have someone competent to refer to...since they don't practice exercise science, they often don't know how to choose a practitioner other than the loudest advertiser and/or pt. description of interactions. Unfortunately, with pts this choice is often based on personality meshing rather than a knowledge of practitioner competency |
|  | I doubt provider education efforts will be as fruitful as systems for referral. only a select # of providers will actually be able to or want to know enough to prescribe. this needs to be outsourced, but integrated into the EHR |
|  | I think we need to be careful with oncologists delivering exercise education - specifically to patients with contraindications to exercise. |
|  | How the Drs and teams will be reach? as a group? in person? online? is there a patient advisor involved to show the benefit of early referral for exercise and impact in recovery and QOL |
|  | I think exercise related competency and capacity needs to be enhanced, but the focus on the oncologist is concerning. There are others on the team who may be better equipped to advise patients. Oncologists can endorse and encourage, but I don't think will assume the "expert" role on this issue. |
|  | Should exercise counselling be a priority for oncologists? Visits are typically short, and they have a number of issues to address during their visit. Recognizing that telling patients to "exercise" or "walk more" and to instead refer them to another clinician for motivational interviewing/counselling would be helpful. However, I do believe that oncologists should know the value of exercise in cancer patients and have exposure to current guidelines. |
|  | multidisciplinary team approach should be emphasized not have everyone become an expert. Appropriate knowledge of risks and referral |
|  | I've worked at several institutions where this is already established, and it does not increase likelihood of exercise being offered as standard of care. |
|  | We are assuming that exercise will be effective for all patients at all times and that oncologists do not recommend exercise only because they lack education about the benefits of exercise. I don't agree. We can't assume the evidence is good enough to convince all oncologists. If we truly practice EBM, we would be less belief-based and more nuanced in our recommendations. I think we need to be honest about the evidence (e.g., no phase III trials) and the levels of (un)certainty in our recommendations. |
|  | Not sure that educating care providers on the IMPORTANCE of exercise is sufficient to change behaviour. Lots of KT/D&I literature in other areas to show that this is not the case so while I agree that the goals identified are necessary there is a big gap between the goals of this priority and the anticipated impacts. |
|  | As above, I'm not aware of literature supporting lack of interest/motivation on the part of the health care team and using this wording within future research could limit accessibility to clinicians). Also, perhaps using the word "oncology clinician" rather than "oncologists" would incorporate nurses, pharmacists, etc. to be included. |
|  | Need to emphasize the multidimensional barriers that influence the competence and capacity for oncologists and PCP's to promote exercise--i.e., unlikely that this research priority will address these factors |
|  | Education, very very reliably, does NOT alter behavior. |
|  | Support with initial engagement as some oncology consultant teams are large therefore there is a lot of staff to coordinate into training that they may not feel they need to prioritise when managing large caseloads |
|  | Would there be designated group of professionals in charge of educating oncologists/primary care givers? Definition of cancer survivor? |
|  | To my experience, the main reason why physicians do not talk to their patients about exercise is not missing knowledge but missing time. Therefore, knowledge might be of limited value. |
|  | If the evidence is not in the area of clinical outcome interest of oncologists, uptake may be hindered. |
|  | Should exercise counselling be a priority for oncologists? Visits are typically short, and they have a number of issues to address during their visit. Recognizing that telling patients to "exercise" or "walk more" and to instead refer them to another clinician for motivational interviewing/counselling would be helpful. However, I do believe that oncologists should know the value of exercise in cancer patients and have exposure to current guidelines. |
|  | Need to clarify expertise of the "researchers" |
|  | Maybe combine Research Priority 1 and 2 |
|  | Education should focus upon exercise related behaviours with evidence of efficacy. |
|  | Evidence-based information to these targeted professionals will be important for allowing them to make informed decisions. This evidence could include the effects of exercise (or lack thereof) on tumour growth in different types of cancer, which is not a complete body of literature. |
|  | maybe instead of 'oncologist', it is more generic to the broader healthcare team that manage the disease (i.e., inclusive of nursing, family physicians, etc.). |
|  | Oncologists’ attitudes and beliefs towards exercise is not the issue - in fact recent data by Ligibel et al. supports this. In addition, oncologists should not be the individuals providing exercise counseling to cancer patients; they should recommend but not counsel |
|  | I'm not sure I would change anything, but I think that it's really important to recognize the huge number of things that PCPs are supposed to be doing. They simply can't do it all. |
|  | Oncologists need to recommend exercise as part of their standard of care for people with a cancer diagnosis. |
|  | stated above about the inclusion of other specialty providers who may be the source of referrals to exercise |
|  | I think it is important to clarify to physicians/primary care providers that they do not need to make the exercise prescription. They do need to encourage and endorse. |
|  | Not a concern but just a stress on how important the promotion of exercise and activity should come from the oncologist. |
|  | challenge to reorganise health systems, and much relates to finances |
|  | training needs to have some reward in order to not just get those interested already and be able to fit in their very busy work lives |
| **Not Actionable** | It is also important that the oncologists themselves as well as their nurses and PAs are also exercising. |
|  | This should be mandatory and in the absence of adherence should be punishable. |
|  | see above |
|  | This is a "6" |
|  | How would this happen? |
|  | this is very good priority - especially the oncologists who have the authority positions to reinforce the value of exercise to patients but don't do so. |
| **Different Theme** | A guide for local resources |
|  | Engaging oncologists on the benefits of exercise is important - however, an exercise professional should educate the survivors on the benefits of exercise in a separate visit |
|  | More of a comment not a concern. In terms of impacts - I personally think that the general culture needs to change, including that of the oncologists. I am just not convinced they should be the ones providing exercise counselling. The referral and endorsement is where I'd focus. |
|  | Speaking on behalf of physiatrists, we can be positioned to assist oncologists with exercise counseling, and with helping to manage impairments, so that exercise become more feasible for patients that have barriers. I am just indicating that here, because I am not sure of the best place in the survey to make this point. Oncologists can help with exercise counseling in general, but they might not be able to (or have time to) really see it though in challenging clinical situations. |
|  | Oncologist adherence - current mind frames are ‘no time’. Paired rehab professionals to patients would generate better outcomes. |
|  | Yes - identify and simplify referral pathways for clinicians |
|  | The expectation of knowing where to refer if you are a tertiary centre with people travelling from all over the country |
|  | Determine their roles within exercise referral |
|  | Ready access for oncologists to refer direct to exercise medics, physiotherapists, physiologists -avoid overburdening the busy oncology clinician but provide brief education and referral especially for complex patients |
|  | Though education for oncologists and primary health teams is very important, time is often cited as a barrier. Emphasizing methods to refer to exercise specialists may be more manageable for primary health professionals |
|  | Clarity to providers on when to refer to PT vs kinesiology/exercise physiology. Is patient function at a level to safely participate in a regular exercise program, or do then need PT first? |
|  | I do not think it is the Oncologist role to do this, I think they should provide a referral to the HEQ for exercise counseling |
|  | I support clinician education and motivation to initiate exercise conversations, however I do not believe that the oncologist will take on the role of exercise counsellor. Suggest focusing on referral mechanisms to appropriately trained support staff. |
|  | would be beneficial to include not only oncologist but also general physician who can make a referral to the exercise program |
|  | availability of exercise programs for patients - just because the doctors have the tools doesn't mean the patients will use them. |
|  | Commitment of practitioners to exercise. Only believable if they exercise themselves. Should also align with nutritional counselling, of which I received none. |
| **Round 1 Theme C** | **Details** |
| **Title** | -- |
| **Goals** | "scope of practice" feel there is room for many layers of health care professionals, including professional with certification who can help the oncology population, not sure we need to define a scope of practice |
|  | In some hospital settings in the UK, there are shared competencies between OTs and physiotherapists. Clearly delineate scope of practice could make the impression that there is no space for shared competencies. However, shared competencies can be useful in situations where there is a lack of certain professionals. |
|  | delineating the scope of practice between different professionals might be difficult, impossible, or politically ugly--I might try to be more inclusive and not split the exercise community |
|  | I would be tempted to differentiate personal trainers and QEPs since both can perform valuable yet different roles |
|  | have some form of way to be recognised as a specialist |
|  | I think new teachings encompass exercise as a potential therapeutic for patients. Especially in the rehabilitation, physiology, and kinesiology backgrounds. I think there should be more emphasis on professional side. |
|  | Under goals remove line of increase number QEPS in urban and rural communities and under impacts remove line of increased accessibility to QEPs |
|  | Examine what currently exists to build best practice. |
|  | clarify which undergraduate/graduate programs and/or type of professionals would be included in the scope of this research priority |
|  | Standardization is great as long as there is emphasis on specific patient-based needs |
|  | The focus should be on the postgraduate level. Undergraduates (in Australia) are exposed to chronic and complex conditions inc. cancer in their final year of study however, further knowledge generation should be emphasised in their postgraduate degree. |
|  | Clarify education programs for which clinicians (e.g. fitness professionals, PTs, OTs, MDs, etc.) |
|  | include continued education. changes in practice and updates to guidelines need to be disseminated for those that already work with cancer survivors. |
|  | really consider non-baccalaureate trainers. this is BY FAR the majority of trainers in the U.S. |
|  | I would be tempted to differentiate personal trainers and QEPs since both can perform valuable yet different roles |
|  | Clearly outline what disciplines are being targeted here. |
|  | Please specify the term "diverse exercise professionals" |
|  | My experience in working with APTA physical therapists is that the belief is that a PT knows as much as an EP about exercise which generally is not true. I believe it may be an uphill battle with the APTA. I don't think it has to be if communications are skillfully managed - then it will become an opportunity. |
|  | I would suppose there is lack of time and resource to include this in existing undergraduate etc. programs (?). At least in my country where pain treatment knowledge is lacking, and that is even more basic than exercising..... |
|  | training should be integrated into existing education but there needs to be a clear pathway to utilising this training in practice (i.e. a specific graduate’s job/role) |
|  | Changing scope of practice results in fundamental changes in how a profession practices. An overly ambitious undertaking alone much less in the context of what this document is seeking to achieve. |
|  | As above--delineating the scope of practice between different professionals might be difficult, impossible, or politically ugly |
|  | How would consistency/quality be evaluated? |
| **Stakeholders** | Add patients as knowledge mobilizers |
|  | Consider the inclusion of Physiotherapists in this category too. Given our health system in Canada, Physio's are the easy referral pathway for Physicians, yet right now graduate with minimal knowledge on exercise & oncology. |
|  | ? add exercise and rehabilitation professionals? |
|  | Would like greater clarity about which QEPs this includes? Perhaps list example professions |
| **Impacts** | Add: "Improve oncology-specific assessment, exercise prescription, and physical activity promotion skills" to Impacts. I think it’s important that this education will have a practical skill-based component if delivered in universities or clinical settings. |
|  | Concern that implementation in urban environments may have to be very different than in rural ... |
|  | Entities such as CAPTE need to change program standards for PT programs to ensure the inclusion of oncology content within curricula. |
| **General** | This is only because Australia already has a standardized exercise oncology curriculum, accessible community-based continuing education training opportunities and fairly decent scope of practice documents |
|  | There are many cancer exercise trainers/professionals working in the field that hold various certifications. It's important to increase accessibility of trainers while not limiting those already in field because they don't have the "gold standard" certification. |
|  | I think we can benefit from exploring online education modules to reach HCP and professionals that may not have access to higher ed. There are some great examples from practitioners in nutrition and S & C that have set up online "Universities" that have a great deal of rigor. Ultimately, I think we need to find ways to developing levels of pitch with content/duration of study etc. aimed at people from all backgrounds. I think it's also important to find some sort of consensus on industry standards on minimum KSA's needed to work in this area. At the same time, developing modules aimed at professionals in different settings (i.e. content required for a local PT vs. individual preparing to work in a cancer clinic). |
|  | Using popular social networking system to maximize the accessibility to the knowledge is also useful |
|  | Potentially involve technology and social media solutions |
|  | The THRIVE QEP resources by Dr. Culos-Reed et al., are the best cancer-specific exercise training/education resources I have used |
|  | not really a question/concern, but I don't think that this is a high priority 'research question' - it is a high priority area of the broader field. a research priority might be to evaluate the quality/effect of care by different people with different qualifications. not sure this is the same thing. |
|  | clarify "researchers" |
|  | Fine if we make claims based on the strength (level) of the evidence. We cannot give targeted exercise prescriptions by cancer and outcomes based on the current evidence. If we give recommendations based on epi studies, we must highlight the limitations of this evidence, and the same applies to RCTs with low internal (and external) validity. |
|  | What considerations are being made for the paediatric and teenage populations within this as their exercise and physical activity needs are different to an adult’s? |
|  | I am not sure if the training needs to be consistent between all providers, as each provider has different qualifications. However, it would be good to have some basic expectations of the training. |
|  | Need to consider specific backgrounds and pre-existing knowledge and skills and attitudes across different communities and geographic / economic backgrounds to ensure uniformity so as to give all patients and carers equal care/treatment and to reduce any indirect discrimination |
|  | It can't be one size fits all, a rehab professional, hopefully physiatrist, will have to customize a safe and effective exercise program |
|  | Most gym coaches/personal trainers lack sufficient knowledge in (exercise) physiology. Anyone who completes a 10-week course can become a personal trainer making it more challenging to incorporate additional training that would give them sufficient knowledge to take care of/handle exercise needs and considerations for cancer survivors. |
|  | Is there going to be a standard education level for exercise professionals? |
|  | How are the rural needs going to be met? |
| **Not Actionable** | Pickleball is an amazing easy to learn sport - a game changer for many people especially those dealing with a medical challenge. It's fun, easy to learn and very social. Getting back to playing the game was instrumental to my recovery from a cancer diagnosis. I know this study is about traditional exercise programs. However, I do believe this one sport should be included in your research. |
| **Different Theme** | Education around the different types of rehab and exercise services, e.g. when to refer to PT or more general exercise providers. |
|  | Being a 6hr round trip drive from my treatment centre meant I wasn't able to take advantage of many of the wellness programs on offer to people who live in the city. Perhaps focusing on some online resources would mean urban and rural stakeholders could benefit equally. |
|  | Need a goal for increasing patient/clinician ability to FIND these individuals |
| **Round 1 Theme D** | **Details** |
| **Title** | -- |
| **Goals** | Hard to reach and understudied populations are not the same thing. These should be separate goals. |
|  | Add a note about dissemination and implementation. |
|  | Decrease number of goals for this research priority |
|  | Mention how exercise fits into an interdisciplinary allied health/rehabilitation care team. |
|  | Identifying the barriers to access in addition to the awareness is equally important. |
|  | Just a note that this would also apply on a country level. So, more research in lower-income countries, because the priorities and barriers to exercise will be potentially be very different and therefore, limit generalisability of existing literature. |
|  | Not a change, but a focus on rural and underprivileged areas would be ideal |
|  | Understand the most common ways people exercise in rural settings and how this may influence participation or realistic recommendations |
|  | other priorities could be relevant for hard to reach populations so not sure this is something that should be standalone |
|  | I'd like a little more detail about what is meant here. It's a bit vague and difficult to identify what the goal is. |
|  | It is important to consider a wide range of hard to reach populations, beyond cancer type, and ensure that a health equity lens is applied. |
|  | Agree that much of the research is in breast, colon, prostate, etc. I'm not certain that showing that exercise also improves fatigue in another population (for example kidney cancer - where there is only surgery and so far no adjuvant therapies OR thyroid cancer - less people but very similar drugs to other cancers) is worth the money. Quite likely, the evidence from the other cancers where drugs have similar side effects (fatigue, weight gain, hyperglycaemia, anxiety/depression) then knowledge about benefits of exercise on these issues are transferrable to other types of cancers. In head & neck cancer, there are quite unique survivorship needs, but this has already been studied. Therefore, I think it's important (if there is a gap in a SPECIFIC cancer for a specific reason) then this goal should be re-worded to clearly list which cancer and what the need is. |
|  | Want to include increase funding for these support services (financial is a common barrier) |
|  | This will obviously look strikingly different depending on the community, so it might be important to consider the impossibility of standardization and how that will play out in a standardized/coordinated effort. |
|  | many exercise oncology resources available at the moment are offered by non-profit organizations that provides for a particular type of cancer diagnosis. These organizations will often rule out other types of cancer patients. Making sure that patients suffering from perhaps a rarer form of cancer benefit also from exercise documentation and resources is mandatory. |
| **Stakeholders** | NPOs should be stakeholders |
| **Impacts** | Under impacts, point #2, I don't this this needs "the most effective". Determining effective strategies for hard to reach cancer populations would be a huge step forward and provide multiple options to address their complex needs. Given so little is known, building this knowledge base would be my first step. Multiple solutions are likely needed. |
| **General** | I think there is a big difference between understudied cancers and rural/remote cancer survivors. Perhaps consider separating these |
|  | Include other types of cancers |
|  | not clear what is meant by "hard to reach", do you mean geographically, o those with other barriers perhaps educationally, or those with competing stresses and life demands, etc. |
|  | With less than 30% of cancer survivors meeting current exercise guidelines, limiting this priority to "hard to reach" excludes huge numbers of survivors, thus seemingly misses the mark which should be to reach cancer survivors period. Why limit this goal to those with understudied diagnoses? How is understudied defined? |
|  | Unsure what ‘understudied’ and ‘hard to reach’ refers to and why specifically targeting these groups. |
|  | People are not “hard to reach”, it is science and research that is “hard to engage”... this language needs changing! Learn from the field of HIV. Hard to reach is alienating and othering. |
|  | It may be useful to define hard to reach and understudied |
|  | "Hard to reach" and understudied are two separate groups, with potentially different features and potential impacts. |
|  | More clearly define ‘hard to reach’ |
|  | Hard to reach is not the same thing as understudied. I think you are trying to get at patient disparities. It's really two different things--make it easier to reach some populations and do more research for understudied populations. |
|  | I'm unclear what is meant by "needs of hard to reach & understudied cancer populations" - this is quite vague. If better defined, this could help better understand this aim. |
|  | Define what you mean by "understudied cancer populations" |
|  | Define hard to reach and under studied populations |
|  | This is important but very limited evidence in the first place that exercise is safe, tolerable, and efficacious in these populations (it’s also not clear what these populations actually are) |
|  | I'd say in order of priority this may be a second level one, so that the systems are in place and then the outreach is broadened; plus, the barriers for these groups is likely very different than for well-resourced cancer patients |
|  | Focus on patients with co morbidities; long term outcomes |
|  | In the US, much of this work would have to do with facilitating patient access to and payment for exercise oncology programs --unsure whether that is the same problem in Canada |
|  | In addition to how to research such patient groups it would be good to pool worldwide resources (multi-national studies) to develop the evidence base for the effect of exercise intervention in these groups. |
|  | So, how to do this hard-to-reach work?? Grant opportunities? other innovative ways? |
|  | I think the promotion of exercising with/beyond cancer needs massive work in the UK before the specific promotion within novel groups, for successful implementation. |
|  | How will these understudied populations be recruited? |
|  | My concern is that we are having difficulties reaching well-funded centers, how are we supposed to impact low income/difficult to reach centers if we have not achieved this in easy access locations. |
|  | It would be good but is it realistic as there are over 200 different types of cancers? Perhaps focusing on the major symptoms and try and find grouping for similar conditions/adverse effects. |
|  | how do you plan to find hard to reach cancer patients/survivors? |
|  | I understand the need for specific tactics for hard to reach & understudied cancer populations, but my concern would be that it could get complicated by having too many variations depending on the cancer type. I think the motto "Keep it simple" would work the best if your are trying to implement a standard program across a great population. |
|  | How this will done, strategies for implementation? |
|  | This piece is so huge, so while I agree this is important the steps within (moving from identifying engagement strategies to identify hard to reach groups to implementation strategies) is not very specific to determine a course of action moving forward. |
|  | Less of a concern and more of a reflection. The unique needs of the FN population should be considered at least in Canada where reconciliation is a priority. Opportunities to meet with, learn from and be guided by FNIM as appropriate are key to ensuring culturally appropriate and safe approaches. Building trust takes time and research protocols don't always reflect this. |
|  | there are a lot of under-represented populations who don't have access to same basic level of healthcare - pleased to see this as a priority |
|  | understudied populations with small sample size --> difficult classical RCTs --> novel methodology for build evidence in these populations |
|  | what is classified as hard to reach? |
|  | clarify expertise of "researchers" |
|  | How are you defining 'hard to reach' and how are you planning to find them to engage them? |
|  | Define " hard to reach"? geographic, language, socioeconomic status? |
|  | Don’t use “hard to reach” |
|  | Are you referring to hard to reach groups or specifically those with cancers which do not have the same level of research evidence for exercise? |
|  | Need to ensure all 'hard to reach and understudies cancer populations' are targeted so as not to unknowingly discriminate any groups or certain groups having more of an advantage |
|  | As written, this research priority strikes me as being arbitrarily narrow, poorly defined, and failing to miss the mark for providing exercise programs to cancer survivors. |
|  | Not sure if "hard to reach" and understudied as groups terms should be used together/interchangeably, depends on definition of who is in those groups. |
|  | Definition of understudied cancer populations |
|  | "hard to reach" is not by geographic location. E.g. some that live in GTA where there are survivorship programs may still have difficulty accessing these. |
|  | Define “understudied and hard to reach” or provide examples |
|  | Be clear on who understudied and hard-to reach populations include |
|  | Who is going to say no to this? But for it to have teeth (really make a difference) there need to be more specifics. (Perhaps that comes later?) |
|  | How will underserved and hard-to-reach cancer survivors be engaged? I would also urge this group to consider cultural differences when engaging underserved populations. Developing resources that are culturally relevant is very important. |
|  | How will these hard to reach populations be identified? |
|  | Costs and other barriers to implementation are difficult to overcome in rural states in the US |
|  | In the US, much of this work would have to do with facilitating patient access to and payment for exercise oncology programs --unsure whether that is the same problem in Canada |
|  | Would access to funding and support services have a major influence on the execution of these goals? |
|  | Implementation is critical to address straight away. Please see "How to Implement Evidence-Based Research" Trisha Greenhalgh, Wiley 2018 if you've not already seen. I expect that implementation is addressed further in this survey. |
|  | Just try to ensure that the people that you want to reach want to be reached by you/us. I've seen many NGO come and stay or dissolve yet never actually reach certain targeted populations within a specified broad group, mostly due to organization higher-ups' ignorance regarding target population. Learn from examples of missionaries everywhere who idealistically try to bring one manner of thinking into a group hoping to supersede the historical m.o. |
| **Not Actionable** | Per previous question |
|  | as above |
|  | Language for all so far. Didn't realize the if so what |
|  | I feel that this is important but would rate it lower down as a priority allowing efforts to be first directed where the impact might be the greatest be the f |
|  | It is important but should maybe not be started with this smaller group if resources are limited. Therefore my "not so important' answer. |
|  | Excellent approach -- the hard to reach populations probably need this most. |
| **Different Theme** | -- |
| **Round 1 Theme E** | **Details** |
| **Title** | Not sure why the word 'diverse cancer survivors' are used; not very clear what you mean by this. Suggest instead clarifying rural/remote and low SES cancer survivors. I would move the wording from Research Priority 4 around 'hard to reach cancer survivors' here as it better aligns with Research Priority 5. |
|  | Definition of technology - what scope would this be? |
| **Goals** | I would just add to evaluation of these methods, to research how these methods work and for whom? What is the underlying mechanism making technology-based interventions work? Do you base these interventions on certain behaviour change theories? |
|  | As with other priorities, this should focus on technology to support exercise behaviours with evidence of efficacy already. |
|  | Specify when testing interventions that they address not only initiation, but also maintenance of exercise. |
|  | Make it more available |
|  | More clarity on what is meant by technology-based, by providing examples |
|  | Identify economic needs of the population being targeted and make sure they can and are willing to purchase the necessary technology for this type of initiative. |
|  | Add in external motivators, group work, and competitions to improve buy-in |
|  | Maybe another goal should be: Determine how to integrate effective technology-based support strategies into existing oncology clinical workflow or survivorship resources. |
|  | Not just the access to technology but the access of access to virtual care and exercise clearance from HCP that may not be available in area. |
|  | Be aware of the limits of some groups (older, socioeconomic) for app and tech-based solutions |
|  | I think this is a great idea, but I am concerned about the safety. I think this would be good for populations that are not at great risk. Supervised exercise is safe and more effective. |
|  | I believe this is vital but more important is the fact that this can be sustained. How do we make sure that this is a sustainable model? |
|  | technology based meaning apps that tell you how to exercise - this could pose a potential problem in regard to self-motivations in survivors |
|  | It is still little known and not specific to the cancer population. It is a strategy that needs to be measured in research settings. |
|  | Keeping up with rapidly changing technology will prove difficult as it has historically. Potentially thinking about platform-agnostic methods will help. |
|  | Having resources available for someone that isn't technology minded. i.e. online training, chat available to help get people started |
|  | technology - apps etc. need to contain the adequate information to tailor to specific side effects and safety + updates of the information |
|  | I like the idea of researching which type of technology (and perhaps the optimal amount of technology vs in-person) required for an exercise intervention. I agree this is a current research gap and could help inform development of more rural programming. |
|  | This seems to overlap somewhat with the priority regarding novel approaches to increasing access for hard to reach populations |
|  | Ensuring psychosocial support if friends and family are not supportive of exercise if survivors are in an isolated location |
|  | Goal 2 the phrase " suggests that technology will be used to do the evaluation when I suspect that authors wanted to say ". "Develop & evaluate evidence-based exercise support strategies that use technology to meet the needs of diverse cancer survivors. |
|  | While the focus is on cancer survivors, such a platform could be used to address the exercise support needs of many chronic disease survivors...is there benefit in a collaborative approach that maximizes resources for CD survivors including cancer survivors. |
|  | same comments as for priority 1 related to how this information will be disseminated and by whom. Also, more research is still needed to determine the best technological mediums to reach a large number of cancer patients and survivors |
| **Stakeholders** | consider adding data analysts or bioinformatics specialists - to stakeholders to ensure data compliance and also assessment of technology use this also allows GDPR compliance in EU, UK |
|  | Are there web designers included to make the technology easy to use? |
|  | Add NPOs as stakeholders |
|  | Adding a Certified Therapeutic Recreation Specialist to the stakeholders |
|  | Involve social workers and occupational therapists. |
|  | need to increase integration within the electronic health record so also include health system IT representatives |
|  | Utilize the expertise of health coaches |
|  | clarify expertise of "researchers" |
| **Impacts** | Add to impacts: increase stakeholders' awareness of technology-based exercise support |
| **General** | Build an online community for support - team approach - to help people get motivated and continue |
|  | Rather than create new, perhaps research what existing tools might be useful for cancer survivors? There are already so many fitness apps, devices, trackers. |
|  | Technologies are becoming an integral part of people’s daily lives and their application in exercise oncology can provide an easy and cost-effective way to encourage physical activity |
|  | I don't value this as much in terms of a priority, many people just need structure and support. Fitbits are great. What I can suggest is the use of e/tele-health to keep in touch with patients, reducing their need to visit specialised clinics for appointments to be done remotely. |
|  | Developing the user-friendly technology-based exercise program is key point to maximize participation |
|  | I think this is important, but maybe not as good/promising as the referral pathway with real people. I think technology can wear out too |
|  | Perhaps merge this with previous question - a strategy to get to hard to reach groups? |
|  | Give examples of technology-based exercise support strategies |
|  | Remove the word diverse. It carries with it the risk of being too limiting. |
|  | Definition of technology/what is the scope |
|  | Research priority #5 overlaps with Research priority #4. Maybe you can combine both |
|  | Seems like this should be combined with the previous 'hard to reach' population goal |
|  | There is an assumption that increased technology would result in benefit. I think we need to establish whether wearable tech actually offers any advantage over no technology at all. And also whether the potential effects of wearable tech is sustainable over the long-term. |
|  | This is something I have little experience of but can see how it's role could be vital in certain communities and its scope be far reaching |
|  | Considering the unique needs of patients/survivors, there would be significantly better outcomes with one to one support. |
|  | YES YES YES. As a cancer survivor who lives rurally the online community and resources have been SO important from getting me access to education, I wouldn't have otherwise had to connecting me to others and making me feel less alone. |
|  | Accessibility of technology may be less in the hard to reach populations. Some of which are also struggling with poverty, marginalization (waiting beside prisoners at PMH had a strong impact on me), aging |
|  | technology has wide reach across population segments, and I can see already some cross-cutting priorities where technology-enhanced strategies are relevant |
|  | I find patients have very very little engagement with any online resources I encourage, even if they are well developed. |
|  | in what languages would the information be provided to remote communities, i.e., indigenous, newcomers, etc. |
|  | As above, I think exercise referrals need to be "stepped care" where some can be delivered via technology, but others might need to be in person |
|  | While important, technology is always changing so challenge is keeping it up to date and relevant; so, discussion of how to best put efforts here that will have sustainable/realistic impact |
|  | as above. I think exercise is absolutely critical to state of mind and to physical well-being when in treatment BUT in treatment one often looks and feels dreadful (walking in my back alley in Toronto I was twice mistaken by neighbours for a homeless wanderer. the response was one of caring, but it was also embarrassing. I was in the alley and not on the road because I knew I looked awful. A person less committed to exercise might have found this discouraging). when one isn’t eating properly because of treatment, exercise can also be more difficult. |
|  | Focus on evaluating existing technology-based interventions rather than new interventions |
|  | What are some perceived barriers regarding the use of technology? Retention and engagement with the technology? |
|  | Might be especially important in countries with long distances to training centers. Many aspects are better when seeing the patient. |
|  | Is this one area of inquiry under the umbrella of the previous strategy (ways to engage hard to reach populations) |
|  | Is this primarily focused on remote monitored exercise programs? |
|  | To clarify, will these patients be screened for safety to exercise prior to enrolling in such a program? This would be amazing to have in Canada where patients are so spread out, but we would need to confirm that safety to exercise was addressed, much like other populations. |
| **Not Actionable** | quote from cancer survivor - we have enough apps and also older adults / hard to reach less likely to use or benefit? Face to face is best |
|  | I think this is a great initiative. Someone who has to go the doctors once/twice or three times a month for treatment/check-ups probably wants as little as possible to do as far as in person meetings. |
|  | Leveraging technology is going to really help us level the playing field for all cancer patients, regardless of location/SES/etc. |
|  | I believe that technology-based strategies is a direction that both cancer and other chronic condition based rehabilitation programming needs to focus on. This will improve engagement on a larger scale and meet the needs to patients unable to travel to certain areas |
|  | This addresses my concerns with the last priority. |
|  | Again, while this is/will be important I believe that should be ranked lower as a priority, aiming efforts at the primary care givers and the patients with KT |
| **Different Themes** | I think exercise referrals need to be "stepped care" where some can be delivered via technology, but others might need to be in person. I recommend incorporating this risk/need stratified approach here |
|  | Some of the elderly population are not on board with technology. Make sure messages come in varied formats. This is a great avenue for education and even exercise training. |
| **Round 1 Theme F** | **Details** |
| **Title** | -- |
| **Goals** | Another goal that could help would be to establish a form of communication/scholarship between cancer specific QEP in each country. For example, it would be very helpful to be able to communicate with other ACSM-CET's about their practice and unique barriers-- also to collaborate for research or grant writing. |
|  | I think work in this space could be directed at educating QEP's how to get a "seat at the table". Education for QEPs could be how to connect with, engage and communicate with stakeholders and HP's to develop partnerships. We need more education on the language of stakeholders and how to pitch our value to them. |
|  | Clarify role of different types of exercise professionals. Usually, health care teams already have professionals (PT) for the most impaired patients. |
|  | Add a standard for determining qualifications for QEP internationally |
|  | Define the role AND scope of practice of a QEP in the cancer hospital setting (this is different than current scope of practice) |
|  | a goal to have the integrated into the MDT and decision-making process |
|  | consider adding list on included professions for clarity |
|  | This is similar to my previous response where QEPs and personal trainers should have separate and defined roles. This section is very important to the profession and to implementing exercise into healthcare |
|  | Consider adding goal #3: Provide a model of how QEPs may be added to primary care settings |
|  | Consider economic burden of the approve approach |
|  | I agree with this priority. Further, a step towards facilitating this could be the creation of colleges for Kin or QEPs, as this is not a thing outside of Ontario. This would have to come from the universities and professionals and is not just specific to cancer care. By having a college, the professionals may be more recognized as allied health, thus facilitating trust and integration |
|  | Set standard education and certification of QEPs. |
|  | Embedded into secondary care i.e. from diagnosis onwards |
|  | I think many systems barriers are known. It's more about training QEP and seeing how they are integrated in other chronic disease management (cardiac, nephrology, diabetes) and replicating/building the case in oncology. |
|  | extend this beyond the oncology team to address the transition period out of active treatment to long-term survivorship. this would involve primary care as well as oncology. |
|  | The "goals" (i.e., roles and barriers) for this research priority will likely be very different between countries. |
|  | Government funding organisations need to be listed or considered. Allocation of public health funding specifically for exercise physiologists does not exist. This is one of the primary barriers to increasing referrals and increasing the number of exercise physiologists in public health. |
|  | The only hurdle I see here is making this billable and/or obtaining buy-in from hospitals to pay for these team members. |
|  | Greater need for role clarity and boundaries between "qualified exercise professionals" in teams, as many provinces only provide services to the most impaired patients via PTs. |
|  | Collaboration between PCP and QEP professional organizations would help w/ development of referral tools and consistent massaging across all providers |
|  | Also important for integration in tertiary care |
|  | If the goal is to have exercise embedded as part of standard practice in cancer care, then QEP's must be integrated into the care team. |
|  | What is the baseline qualifications to make these decisions? |
|  | Ensuring standardised competency framework |
|  | Does this mean a team of centralized QEPs (referred by Oncologists), or one to two QEP's on a primary care team? |
|  | In a setting such as the NHS, given the financial constraints, a system like this however vital could be challenging to implement. It would probably require upskilling and educating an already established work force within primary care. How would its success be measured? |
| **Stakeholders** | Ideally this would include whatever clinician the patient was seeing for their "primary care" e.g. the case of the prostate cancer survivor who only sees a urologist and doesn’t have a PCP |
|  | include third party payer representatives (insurance, etc.) |
|  | Government funding organisations |
|  | CSEP & CKA |
|  | Does community partners include charities and independent exercise organisations? Is it assumed that the health providers maybe private &/or public sector where those services exist? |
|  | I'd add stakeholder organizations - ASCO, APTA, AAPMR, ACRM, etc. There are challenging knowledge, political, and turf issues. Also, how will this be accomplished? |
| **Impacts** | -- |
| **General** | In a setting such as the NHS, given the financial constraints, a system like this however vital could be challenging to implement. It would probably require upskilling and educating an already established work force within primary care. How would its success be measured? |
|  | This sounds great in an ideal world where funding decisions and policies are driven by research findings but it seems unlikely to be achieved at this point so research should focus on more 'low hanging fruit' - in many settings outside of PMH there is zero support for exercise. |
|  | Why aren't they working as an integrative health team relieving ALL of the burden? |
|  | This needs to go hand in hand with developing approaches that can reach and support rural populations. |
|  | Need clarifications regarding what “primary care team” means. This in HCP lingo is very much liked with general practice/family physician. I think this research priority goes beyond that and terminology might be best to suit the targeted HCP or HCPs |
|  | Agreed that exercise physiologists need to be trained but not sure what the model is to integrate such professionals |
|  | QEP to CTRS/Recreation Therapists |
|  | As always, we need to find a way for reimbursement for this to be put into the medical model. Or increase the importance of how it may cut costs in the long run. |
|  | this may turn out to be a reimbursement challenge - QEP would need to be recognized as a skilled professional |
|  | Costs are always posed as an issue due to billing challenges outside of the realm of skilled rehab |
|  | Not sure this is feasible in US where cost is always an issue |
|  | Skeptical that getting QEPs to be regular parts of health care teams is achievable in the near future |
|  | While there is overlap in many of the providers for cancer rehabilitation/exercises, there are differences. It may be politically challenging to discuss, but for patient safety and transparency it is important that the providers are comfortable discussing similarities and differences. |
|  | It feels like this has to be part of a broader conversation about integration of all forms of rehabilitation. I worry that if we work to only solve the exercise part of the equation, we'll miss the boat on being able to improve all aspects of functioning. |
|  | need to explore the many challenges here. important but it feel it's hard to address |
|  | We're stepping out of the scope of practice |
|  | The only way this will happen is if the medical caregivers appreciate the importance of exercise. As such again I would aim my efforts and resources at educating these individuals |
|  | Working with CSEP & CKA in Canada is going to be essential to get the appropriate advocacy for this. And learning for models that currently exist, such as in Australia and in Cardiac Rehab, to understand how this works outside of research. |
|  | resources would have to focus on the patients’ home city. I live in Brampton but all my oncologists are in Toronto - ensuring the QEP know resources Ontario wide would be important |
|  | the qualifications of the proposed people to "relieve the burden" of primary care providers |
|  | What do you consider the ideal training for the QEPs to be? What should the learn and achieve in order to work with cancer survivors? |
|  | Proper educating and training those QEPs are critical to obtain reliable and optimized outcome for the research and exercise program |
|  | clarify expertise of "researchers" |
|  | language used above refers to primary care team. We often think of the primary care setting as the general family physician and indeed a "team" model is being established in some settings. I'm not clear here if the reference to primary care team reflects primary health care setting or the "primary" team responsible for cancer care following a diagnosis. Two very different settings. In reality a QEP in Primary Health Care could have a significant focus on exercise for CD prevention and at suspected diagnosis, guidance on benefit of exercise etc. during/following treatment. In the cancer care setting, the QEP is required as the expert to provide education, triage, establish appropriate programming...this is key to ensuring oncologists support this work and trust the person working with their patient. |
|  | Assuming benefit, then yes, qualified exercise professionals will be required. |
|  | Define QEP. Does this term include Physiotherapists of the cancer care team that may have experience/training in exercise and oncology? If so, this can undermine the role or the actual purpose of integrating the QEP into primary care cancer teams. |
| **Not Actionable** | if there is education on the undergraduate and graduate level of health care professionals, this would not be as important a research priority |
|  | Great idea! |
|  | I am currently performing these job responsibilities and would be very interested in connecting with others that are also as our workflows may benefit from connecting with each other |
|  | I like the idea of evaluation the integration- hopefully this role could be funded one day. |
|  | I believe this is imperative! In addition to a multi-disciplinary approach of these professionals within the healthcare system |
| **Different Theme** | It depends on the site and resources etc. I think they should have a QEP, but if there are limitations then have VERY good referral & communication pathways to community clinics |
| **Round 1 Theme G** | **Details** |
| **Title** | I am not sure that the title accurately reflects the goals as you are looking at awareness of programs first and then referrals (these can be two separate things-not even knowing what is out there or knowing but choosing not to refer) |
| **Goals** | A prospective and standardized tool for assessment and triage is needed |
|  | I would just add to identifying available cancer exercise services that there might need to find the best way to collate all these services. Maybe database, webpage, registry... etc. And a way to keep this information up to date and easily accessible for professionals and people affected by cancer |
|  | have a way of recognising those specialising in this area |
|  | Research to consider the role of an automatic referral process to be triaged by the QEPs. |
|  | Integrating technology into this idea would be very beneficial, for example, a website easily accessible to both patients, primary care, and exercise physiologists with a list of available exercise programs along with descriptions - so that everything is in one, easy to access, central space. |
|  | Not only should exercise services be mapped, but there should be consideration of exactly what those exercise services are delivering and then consideration of the extent to which they deliver efficacious services. This might then help with evaluation if translation of efficacy to effectiveness. |
|  | incorporate the idea of understanding what level of "stepped care" of exercise that patient needs, e.g. some will need home or community-based referral whereas others need supervised intervention |
|  | Clarify referrals to different types of disciplines. Some patients will need PT before they are able to participate in a general exercise program. These referrals are not consistently made either. |
|  | Define primary care teams. Oncology care team? Primary care teams (i.e., general practitioners). |
|  | Physician referrals are so critically important. But this must work more effectively than PT in other medical communities works now, as feedback from QEP must be communicated back to physicians, not just one-way referrals. |
|  | This priority is a better fit for urban/suburban patients than for some rural patients. When options are limited (few/no programs available) and people have scheduling or childcare barriers, the referral-based strategy may be completely unhelpful. Important to make sure that other approaches are in place to support this subgroup of patients (and I do think you've got that covered through the other priorities.) |
|  | different healthcare systems use different pathways... different software... competing with referrals to other initiatives / charities/ support |
|  | Seems to assume that all cancer exercise programs should require a referral. Does this leave space for the possibility that some patients are able to self-manage their exercise training and/or seek out their own resources? |
|  | Not all centers have access to exercise professionals. For example, in the US, insurance doesn't reimburse QEPs (e.g., fitness trainers, exercise physiologist’s). PTs, OTs, physiatrists, nurses often implement exercise interventions. I would suggest expanding language to include other clinicians for these reasons. Tools must be brief and simple. Even the excellent tool provided in third summary paper of the recently published guidelines for cancer prevention and exercise may be perceived as too demanding in some oncology clinics. |
|  | With so many variants within each location it may be challenging to standardise the referral system. This would not allow flexibility to be able to suit the patients’ needs in that area |
|  | Cost is one of the biggest barriers here. Many regions will not have free or low-cost options, so looking into how to provide information on all of the options is pretty critical. |
|  | Not having an adequate referral system will reduce the reach of these programs to survivors despite all the efforts in forging them forward |
|  | Is there any way to have a medical related financial analysis of this? |
|  | This seems very broad, specifically when stating "within a region." Difficult to identify how this would be accomplished when so vague |
|  | language around primary care team - clarify. Adding new responsibilities to the cancer care system can result in significant push back if the processes are not streamlined and efficient. Understanding how this can work best in various contexts is important. |
|  | Would you consider self-referrals? |
|  | When is the best timeline to implement these referrals? Is it a case-case basis or something that should be standardized in the diagnoses process? |
|  | Again, would want to clarify that there's a mechanism to assess safety for exercise prior to referral. |
| **Stakeholders** | Integrate community and primary care in terms of official referral pathways |
|  | stakeholders: ICT - with the development of digital platforms it is imperative that ICT is included as health services move more digital. From experience you can map a patient pathway but adding digital process can be difficult. |
|  | Would be nice to see Universities playing more of a role as community partners. |
| **Impacts** | -- |
| **General** | Integrate community and primary care in terms of official referral pathways, have good communication lines so the clinic gets assessment/prescription results. Finally improve referral process between sites e.g. in Australia the pt. needs to visit GP to get Medicare care plan to see QEP, adding an extra visit. If oncologist can activate this care plan it would be better for the pt. |
|  | Be aware that some health systems (UK maybe Can, Europe (Denmark, Ger), have access to exercise rehab within healthcare system -utilise not reinvent |
|  | I would suggest the use of an electronic referral tool, if possible, to automate the referral system so that clinicians are likely to engage and receive follow up reports via the same pathway |
|  | Not focus on creation of standardized communication tools |
|  | It has to merge with priority where you standardize the training. |
|  | Culture-sensitive programs should be performed. |
|  | Identifying local gyms nationally to collaborate is key importance. Although there is a role for home programmes, integration into a local community gym with support from the staff is required |
|  | although important, this is very location specific and pt. participation (completion of the referral) is dependent on individual preferences and resources; hence, I am not sure this will move the needle on integration with cancer care |
|  | This should go with Research Priority 1 and 2. |
|  | It's unclear what the research question is - this appears to be a list of important tasks to increase referrals. This is likely quite important to do but I don't understand how this is connected to the primary aim of "identifying KM research priorities to adapt ex as a standard of care"? |
|  | I like the idea of survivors and care providers contributing in this area. But, please don’t call us survivors. We are usually people living with cancer. It’s a more positive, helpful phrase. Survivor feels as it one's life is now stalled. |
|  | Again CTRS |
|  | There is a lot of variability in QEP training between countries and organizations-- are all QEP "equal" in this regard? Are some QEP more qualified to work with high risk? |
|  | Have programs for health care providers to refer to. Encourage FHT's to offer an exercise oncology program similar to cardiac or diabetes programming. |
|  | Once capacity is available, referrals will likely increase organically. |
|  | Consistent theme is cancer survivors don't know how to get started, where to go, what is safe. MD's can always recommend PT; however, they are not aware of other safe places to exercise. |
|  | Financial barriers are often a limiting factor for referral when there is not support through the public health system for oncology referrals |
|  | Limited numbers of available programs in rural communities |
|  | What if there are no current local exercise programs specific to cancer survivors? In terms of clinical trials, what if patients aren't eligible but still can be physically active? EDIT - based on the next question, this appears to be addressed! |
|  | Need to be mindful that this is exclusive to geographic areas where there are community-based resources |
|  | What is the quality of the community program that the client is being referred to? |
|  | incorporate the idea of understanding what level of "stepped care" of exercise that patient needs, e.g. some will need home or community-based referral whereas others need supervised intervention |
|  | clarify expertise of "researchers" |
|  | where do people living with cancer fit in to this? |
|  | Again, careful wording will be required with term "survivor" - how is it defined here and for this research? |
|  | Is this something that aligns with Exercise is Medicine referrals? |
| **Not Actionable** | some patients are better to return to their own, normal exercise regimen (gradually) rather than a cancer survivor specific group |
|  | Add pickleball to the list of activities. Exercise needs to be fun. Playing pickleball is fun and includes many other benefits. |
|  | Assuming effectiveness in specific cancer populations, then this will be important. |
|  | this may happen organically through some other priorities |
|  | I just wonder if this is at all feasible |
|  | Again, I have a system in place for this and it would be great to connect with others that are also currently doing this |
|  | This is one of the top priorities!!! |
|  | as above |
|  | I think sometimes reaching out to survivors might not even be to increase exercise "time" but just increase activities of daily living or get the individual to independency again. Maybe more short-term attainable goals to achieve independence, especially for surgical patients or patients that had negative effects of chemotherapy. |
| **Different Theme** | I have been referred to 2 cancer exercise programs and they both have a long waitlist. Increase capacity ahead of increasing referrals. Demand is already exceeding supply. |
|  | Make access free or low fee |
|  | Cancer patients need access to low cost / supported community programs and cancer center rehab programs |
|  | Lack of sufficient knowledge in the community to provide exercise services for patients with cancer. |
|  | How is funding for these programs going to be secured? Is this a goal of this priority? |
| **Round 1 Theme H** | **Details** |
| **Title** | This seems very similar to the previous priority. |
| **Goals** | But how would you increase availability? What evidence-based methods will help to increase availability and accessibility? Just because a service is available, it does not mean people will use it. |
|  | there are a lot of pieces above - financial support is its own issue! Availability and accessibility are linked back to having the QEP. Look at user-pay models, not unsustainable grant funding |
|  | The current wording is not clear on how research would be used to increase availability and accessibility - it seems like the main barrier is cost so perhaps this priority should be oriented around cost-effectiveness research and models for integration into clinical care (e.g., cardiac rehab) |
|  | I would prioritize availability of exercise programs over research priorities that relate to increasing participation and referrals. |
|  | I would make it more accessible for those who may not have money for transportation |
|  | Increased funding for the whole spectrum of rehabilitation services/disciplines, not just general exercise. |
|  | and cancer rehab which includes exercise |
|  | greater emphasis on web-based to reach a broader range of survivors. |
|  | Identify specific oncology needs - especially for neutropenic patients |
|  | Consider something about integration into current care pathway. |
|  | I believe that environmental surveys have done in several regions, cities, centers. Use this information/knowledge to move forward |
|  | Who is paying for these programs? Until insurance covers the cost, why put all the effort forth or does the effort need to be put forth so that insurance will reimburse.? |
|  | Somewhat unclear how to actually establish financial support (i.e., from who? through what funds?) |
|  | Difficult to implement in big countries like Brazil |
|  | Balancing capacity against “access for all survivors may be incredibly challenging. Might need to evaluate program capacity (capacities) and prioritize l/triage referrals to programs |
|  | Ties into increased need to educate QEPs in order to expand cancer exercise programs |
|  | What qualifies a program as an exercise program for cancer survivors? |
|  | At least in Germany a goal is to cover exercise costs by health insurances. |
|  | I just wonder if there would be an incentive for local gyms to offer discounts or free opportunities for survivors to use their facilities. The cost of exercise can be high. It doesn't have to be, but people tend to be more motivated to be active when they are at a location that supports activity. |
|  | would this be implemented at gyms nationwide or solely in hospital |
|  | possible collaborations with existing exercise facilities, from the public and private sectors |
| **Stakeholders** | NPOs as stakeholders |
|  | There is a need to identify how this will be possible. working closely with policy makers and governments will be needed to implement this. |
|  | clarify expertise of "researchers" |
|  | CTRS |
| **Impacts** | Important to consider the time limits on programs as survivors often have difficulty transitioning out of a cancer specific program into the community. Could consider eliminating or extending time limits (e.g., any time after point of diagnosis) to increase accessibility. |
| **General** | People often state that cancer patients can simply access non cancer services for exercise and for a proportion of survivors this is the case but there are a significant proportion of people either because of their disease, the nature of their disease or the individual themselves needs more specialist services that understands cancer and reduces the risk of patients being either undertreated or put at risk because of their disease. This is so often underestimated especially given the drive for "self-management" |
|  | You would need to consider the strength of evidence. EBM is founded on clinician expertise, patient values and preferences, and good supporting evidence. |
|  | This could work well with a HQ centre and satellite centres in remote areas. the HQ team would provide training to all satellite centres and provide a Mon to Fri phone service (including a medical professional) for any patient related queries |
|  | Need to define more clearly what is meant by an exercise program. Length of time, group vs individual, how frequently they meet (does that need to be standardized)? Otherwise standards of what "counts" are vulnerable to be so compromised as to be meaningless. |
|  | Hospitals may want to provide the QEP for the infrastructure. (maybe that's coming up in the survey) Consider the needs of many survivors needing help so the need for several QEPs. |
|  | I'm unclear of what the research question is here. Did you intend for this to be a process evaluation or an implementation evaluation? Or a "RE-AIM" evaluation? Is it a barriers-study of the available resources to inform KT interventions? |
|  | Be more specific |
|  | Will these exercise services be characterized by cancer type, population, patient fitness level, etc.? |
|  | Are there barriers in using technology (i.e. web based) in this population? |
|  | Ensuring individualisation of the exercise programme, including education relating to exercise in everyday activities (e.g. housework) and the importance of finding exercise you enjoy so survivors remain engaged rather than pushing themselves to complete prescribed exercise they don't enjoy. |
|  | This seems to be an overall priority under which some previously identified priorities may be subcategories (e.g. hard to reach populations, use of technology) |
|  | This feels like it repeats, or may be covered, in the previous two priorities around digital interventions and access to community exercise programmes |
|  | Would increased availability and accessibility of exercise programs result in decreased utilization of trained HCPs and increased utilization of exercise programs without benefit of advice from HCPs? |
| **Not Actionable** | Think about including pickleball in your research on evidence-based exercise support services for cancer survivors |
|  | It’s utopic, but go for it. |
|  | Thank you for doing this!!!!! |
|  | Agree this is important but is not a research question |
|  | Addresses my concerns with the previous strategy |
|  | I don't have concerns, I just wanted to indicate that this would be one of my top priorities |
|  | the more local the programme the better engagement and adherence |
|  | I would say this is probably one of the most important and key to making the whole aim of this successful |
| **Different Theme** | There are a lot of centres already created that can support exercise programming. Focus should be more on establishing this as standard of care and creating the referral systems. |
|  | Understanding the differences between level of care provided in different programs will be important and will likely vary by country |
|  | Financial. I, personally, think exercise and a public health approach are of vital importance, but how will you sell this to policy makers? |
| **Round 1 Theme I** | **Details** |
| **Title** | In the title perhaps addressing resource creation and synthesis alongside sharing |
| **Goals** | Mention openness and transparency in the above. Resources should be shared as a default not on "reasonable request". |
|  | I would like to see a statement about this being international |
|  | I'm not sure I completely understand this one- can you provide examples? |
|  | I'm unclear as to what the exact research question is here and how this relates to KM research priorities to adopt exercise as the standard of care. As written, this seems like these are ideas for resources that could be used in knowledge-translation. Is the research here proposed a knowledge-translation evaluation? A process evaluation? |
|  | Make goals and impact clearer |
|  | Improvement on exercise guidelines and manuals and make this coherent and evidence-based across all organisations and healthcare professionals. This will make advice for patients more effective and clearer. |
|  | In the context of implemented programs or programs being implemented, a sharing of resources would certainly be beneficial, but there also a need for better publication and disclosure of the implementation aspects of community programs. Currently, there are very successful programs that have been implemented, but all reporting in relation to the program is outcomes-based. For programs or groups hoping to start a program, implementation-based reporting could facilitate the creation of new programming. |
|  | Develop a community of Practice - like ‘move more’ Scotland |
|  | Comment: Sharing of de-branded resources and materials for adaptation/use in different countries/provinces etc. can be extremely helpful and efficient. There is no need to be recreating the wheel every time a resource is needed. But the ability of communities to brand for credibility with their own health association for example can also be important which is my case for shared ‘debranded’ resources (credit must/can be given") but allowing adaptation and local logos to be used is also important. |
|  | Would like to see more collaboration between institutions. |
|  | I think that this point will need to ensure 2-way knowledge mobilization between academic and community partners. There is so much that both can learn from each other and this should be setup from the start to be a multi-directional exchange. |
|  | The link between academic and clinic is important, but I think clinical integration should be the focus. |
|  | this may be difficult especially as many QEPs work in the private, for profit system |
|  | No concerns, I guess I just need more clarification of the relevance of this. Is it resource sharing in terms of programs, assessments, tools like questionnaires etc.? |
|  | I'm not completely sure I understand this one- can you provide examples? |
|  | The goals and objectives for this question are not as clear to me. So, I'm not sure what importance to place on it. |
| **Stakeholders** | I would include administrators to the stakeholders list. |
|  | Would include administrators of cancer center institutions and university facility as stakeholders to share best practices |
|  | Add national stakeholder organizations, examples listed above. |
|  | CTRS |
| **Impacts** | Make goals and impact clearer |
| **General** | a lot of this happens. what can be done to enhance it systematically should be q of interest! |
|  | Research Priority 8 could go with 9. |
|  | a bit tricky to interpret this - would the research question be about the impact of sharing information; is the research priority itself ensuring that data/evidence is widely accessible/shared. I’m not sure looking at how data are shared is of high importance, but as a cultural aspect of the field and among the people whom we work with, this is important. |
|  | Vetting quality |
|  | Eventually this will happen. Lower down on the priority list |
|  | sharing of intellectual property/bias of original entity for billing capabilities with lack of incentive to share material |
| **Not Actionable** | I think this probably already happens |
|  | As above |
|  | No concerns - will not let me select "no". |
|  | Very important to share resources and collaborate; however, other research goals take priority over this. |
| **Different Theme** | Awareness of impact in all populations not just those for whom research outcomes is the aim or is perhaps more straightforward (i.e. raise the threshold for exclusion) |
|  | clarify expertise of "researchers" |
| **Round 1 Theme J** | **Details** |
| **Title** | -- |
| **Goals** | Not sure if this is addressed in a later objective - but there is a need for maintenance programs as well...once a survivor finishes the standard program in the health care OR community setting...they are often interested in continuing with the program or ongoing exercise. Finding ways to sustain their interest and engagement is important. |
|  | Determine what types of supports are most effective in engaging ongoing exercise in community setting (e.g. fitness buddy) |
|  | Add: Development of partnership between hospital, community and sports clubs in establishing cancer exercise programs in sports clubs and a referral program from hospital and/or community-based exercise program |
|  | Improving communication among different hospitals and cancer centres in order to provide the adequate care for patients that receive treatment at different institutions. Long-term follow ups of patients. |
|  | I think there is a move towards community-based programmes initially. For this to work, we need to get all medical teams educated on local community services. The community services can be accessed for as long as patient requires however exercise professionals should be encouraging patients to exercise independently (in local gyms) or at home for a continued healthy lifestyle thereafter. Re-assessments at different intervals in the cancer community programme would be a good motivation for patients to continue independently. from my experience, this is very important for transition from cancer exercise programme to independent health behaviours exercise programme |
|  | I agree, however there may be a select cohort of pre-exercisers who have high exercise ability and confidence who can go straight to the community program (perhaps with very limited guidance needed from the hospital QEP) |
|  | Where and when appropriate this is a key step and requires a skilled workforce to refer people at the correct time in an individual’s journey to the right services |
|  | Create specific survivorship group classes, preferably including AYA programming |
|  | Consider transferring patients out of medically supported to mutually supported (group of patients). People will have higher rates of success when working in groups. |
|  | I remain concerned about the term "best practice" [goal 2]. Change it to read - Create a decision-making framework that best informs the transition of cancer survivors across different exercise settings, programs, etc.. |
|  | Add implementation to goals. |
|  | why do all patients need to receive 'clinical-based’ exercise training? |
|  | Similarly, it's unclear to me what the research question is here or whether this is an idea for a decision-making tool? If so, is the research question to evaluate the tool? If so, from an efficacy or a usability perspective? I.e. patient and/or provider evaluation? |
|  | This is likely built into the decision-making framework, but there's clearly a need for risk stratification/triage of survivors |
|  | I think this would necessitate behaviour change (maintenance) and therefore, a behavioural framework. |
|  | This will be key to ensuring there is capacity within the system to see those most in need and graduate out to the community |
|  | Access to quality community programs for patients with ongoing disease or persistent functional impairments |
|  | It may be hard to retain patients from clinical setting to community setting. |
|  | How would you decide who gets triaged to community? exploration here may be important. |
|  | screening required to triage to appropriate setting and need simple and effective referral and communication links between clinical and community-based programmes with pathway going both directions |
|  | I was never told there were hospital exercise programs for cancer patients/survivors therefore would the goal to not promote these programs |
|  | What community settings are you thinking of? Can this be as varied as possible to cater for various interests e.g. exercise classes, gym, swimming, walking groups, Tai Chi/relaxation, |
|  | Is community also referring to private institutions such as long-term care homes? |
|  | As above, strategies and tactics for implementation should be developed in parallel. See Greenhalgh. |
|  | Suggest there also be consideration to support the transition to mainstream exercise programs asap so as to help people feel normal post treatment. |
| **Stakeholders** | NPOs as stakeholders |
|  | CTRS |
|  | Again, cost. Who is paying for the community programs? In my experience, finances are huge barriers for survivors to continue with the exercise programs. Shouldn't insurance companies be involved? |
|  | what is a LTW/LTD? are you including dietitians, health educators, etc.? please clarify expertise of "researchers" |
| **Impacts** | -- |
| **General** | In most cases there is no clinical exercise program available, so cancer survivors are starting in the community |
|  | Not all hospitals have clinical exercise settings to fit this need so clinical to community exercise settings and having the infrastructure and financing for this is critical. Maybe this could go with Research Priority 8. |
|  | currently almost no resources in BC for supervised/ hospital-based physio in cancer care (difficult then to focus on transition to community)- as nothing currently exists |
|  | This is part of the referral pathway question earlier! |
|  | Ensure those systems exist in reverse -i.e. more important for a cancer patient to have quick access to MSK diagnostics, exercise medicine if facing a non-cancer complication that could disable them from continuing exercise |
|  | Considerations need to be given to unsupervised or home-based exercise from the start. We tend to think very linearly about the movement of a patient (e.g. from clinical to community to home program). I challenge this idea as I don't believe the research supports this as the only way. So many people are able to do unsupervised exercise (provided it is guided in some way) from the start. In BC, the PCSC Program has used exercise counselling by a CEP to give individualized programs to men with prostate cancer (all disease stages) to perform unsupervised and is a great example of how this can be done. When the bone mets SR is published in 2020, this research will also support unsupervised programs. |
|  | In my opinion, not very important given that some of the previous research priorities have somewhat addressed this "issue". |
|  | could be covered through previous priorities - e.g. referral pathways |
|  | costs of programs in the community |
|  | It seems like this would largely institution/region-specific and it is not clear how useful general research in this area would help (there are more important priorities) |
|  | Limited scope to locations where there is clinically supervised programs |
|  | safety of community and home-based settings |
|  | Although this is a great initiative, it would require extensive interaction with community programs with non-trained QEP. Past experience indicates participants want the same quality of programing. |
|  | Ties into need for increased university and professional associations to train QEP in the community for clinical/hospital-based exercise programs to refer to |
|  | Yes, this is an important cog to maintain the continuous flow of removing barriers that would allow patients to continue to exercise. Without this aspect, there is a high probability that patients will reduce their physical activity. |
|  | May have “phases” of care w/in clinic and in community so that transitions can be tailored to patient - some may need more or less time/ engagement in clinical care, etc. |
|  | Smooth and ongoing transition from medically supervised setting to home-based exercise setting after discharge will maintain patient’s motivation and participation of continuous program |
|  | This will happen at the speciality develops and evolves; as medical health care professionals accept and incorporate this modality/therapy into SOC. Again, a lower priority |
| **Not Actionable** | please see above |
|  | Creating decision making framework is great! |
| **Different Theme** | Also need more services for hospital based more specialized care, as BC Cancer does not currently have a formal outpatient PT program. Need to treat highly impaired population before treating general exercise population. |
| **Round 1 Theme K** | **Details** |
| **Title** | Add 'for cancer survivors' into title and goals - as per impacts |
| **Goals** | Needs high level of understanding of communities as implementation will be diverse city v rural, individuals’ own resources |
|  | Would add different types of programs for different subsets of cancers. |
|  | funding for community settings (will be different in countries like US without universal coverage) |
|  | Add awareness of ability for community programs to afford costs associated with standard exercise support services. This might exclude some sites. |
|  | To strengthen the importance of accountability of those providing exercise program without proper training and knowledge to do that |
|  | outcome assessment is most important for long term implementation and integration into standard of care |
|  | A one-size fits all standardized approach is not appropriate and likely won't meet the needs of a lot of cancer patients and survivors the wording of the priority should reflect this |
|  | Patients are not standard. Standard approach may not work here. Flexible and responsive to individual needs might be better aspiration. |
|  | Standardization is great to begin with, so long as there are graduated levels and intensities |
|  | As I wrote before about standardisation. What an individual needs in relation to exercise advice etc. relates very much to them and even their cancer itself and their stage of treatment. Standardising factors such as "target heart rate" around whether a patient is undergoing chemo etc. is relevant but not the whole programme |
|  | It should still be individualized exercise! This is not clear here. |
|  | Are there aspects of this initiative that might require customization/personalization rather than standardization? |
|  | Might be difficult to standardize given that every community organization has a different mandate. Some flexibility around programming might be helpful within a standard template. |
|  | still needs to be individualised to an extent depending on people and cancer types, not one size fits all? |
|  | I feel we have the evidence now to individually prescribe exercise to pt.’s rather than a standardised approach |
|  | this is also part of the earlier parts - QEP training, referral pathway, and the programs necessary. Some of these seem redundant and should be put together as research priorities! |
|  | I'm not sure if I'm interpreting this correctly. Do you mean that intervention delivery and testing will be the same for every cancer survivor that chooses to exercise at a community centre? |
|  | Add 'for cancer survivors' into title and goals - as per impacts |
|  | Might be important for patients to be educated to identifying QEP that can provide this support. |
|  | regulation or definition of exercise scheme would need to be broad and reflect the diverse programmes available. |
|  | At some point, you may want to stop being labeled as a survivor. Meaning I’d rather have tips and tricks to being a survivor in the "regular" exercise world |
|  | Standardized exercises limit the individuality needed among these patients, due to the wide variety of adverse effects between patients with similar diagnoses. |
|  | Need to balance standardized delivery w/ flexibility to meet patient needs and abilities |
|  | My question would be how do you standardize when you are dealing with so many variations and physical side effects? I would want to understand how specialization could take place in a standardized environment |
|  | Is there literature to suggest that standardization is important? Perhaps from the cardiac rehab research? I'm not aware enough of this literature to comment. While this seems like it would be lovely to have, I'm not aware of any research that states that exact standardization would increase the likelihood that exercise becomes a standard of care or that would improve outcomes for people with cancer. |
|  | Would still need to be patient based, individualized programming if possible |
|  | It would be difficult to standardise for all areas. There will be differences in resources and support available due to weather/ culture/ support networks etc. |
|  | Standardisation is important but also need to account for individualised treatment specific to patients and balance the two between different groups. |
|  | Standardization for higher quality is good, but standardization so that all patients receive the same training irrespective of their individual needs is not good. Please differentiate. |
|  | Use of the word standardized. Surely, we want individualised exercise, so perhaps only the approach to exercise programme design can be standardized? Also, what is meant by "robust assessments"? Do you mean rigorous? I'm assuming it’s the use of reliable and valid measures and a rigorous testing process? |
|  | some standards are important, but standards should not limit community specific modifications to allow for success of local programs |
|  | participant needs and preferences are too diverse for a single option; |
|  | Flexibility is likely necessary in order to properly implement exercise into cancer care in diverse settings. So, a standardized model across the board is likely not as important, as after all, a square peg cannot fit in non-square shaped holes. |
|  | Just thinking about the cardiac rehabilitation model, there are some guidelines, but programs largely vary in many of the listed goals. Standardizing cancer rehab too early may reduce opportunities to learn about best practices. I don't think we are a point where we can say we know the best exercise prescription. |
|  | This is important but it is also important to still treat these patients as individual and remain flexible to their needs, barriers and goals. |
|  | Would want to be sensitive to creating "standards" that could in turn limit new programming and accessibility. |
|  | My concern is whether this research priority can even be studied. Differences across communities are perhaps too great to make this goal achievable. |
|  | Close collaboration with community-based fitness facilities and community centers will be needed to coordinate this |
|  | Need to really persuade / emphasise to community instructors why data collections in important |
|  | inclusion of a government agency overseeing programming standards |
| **Stakeholders** | CTRS |
|  | NPOs as stakeholders |
|  | clarify expertise of "researchers" |
| **Impacts** | Ambivalent about instituting "another governing body"...no time to pontificate on my small soapbox right now :-) |
| **General** | Our local hospital has no clinical exercise setting for cancer survivors. The study I created and ran for my master's thesis is something we have continued at Adams State University, in collaboration with the SLV Health (hospital) and Alamosa Family Recreational Facility. We have pre-post testing and 10 weeks of intervention. Aerobic/Resistance exercise, flexibility, balancing exercises and a group sport activity. We meet three times a week. And, run stats on the outcomes of pre-post testing. Undergraduate and Graduate Kinesiology students volunteer to help and learn in this setting with survivors. A second MS student is conducting her thesis now using this population of survivors. We include all cancer types. We use Fitness Age Testing/QOL and Fatigue survey. The social interaction of participants and students among one another helps to make this a successful program. The CCC has honored us with a two-year grant and funds from The Larry Holder Family have supported these studies. If you have questions about this program or the original thesis outcomes, please contact me at 719-480-XXXX. |
|  | Could be merge with another priority |
|  | not sure I understand the difference between 'clinical' and 'community' based programming as defined in this survey |
|  | (1) what is defined as community (2) are you going to do outreach to suburbs or just major cities with cancer centres - if the later you might find a decrease in participation as not everyone wants to travel to exercise. |
|  | Appropriate triage pathways are needed to ensure the right people end up in these programs, and that the people who need more individualized support get the advanced assistance they need. ACE would be a good model to consider for this. |
|  | This is down the road although super important. |
|  | Great idea! And can be very useful in the future to evaluate effectiveness |
|  | This is also key as oncology care providers are reluctant to refer to programs that are an unknown quantity in terms of quality. |
|  | I love this idea. I currently look at the make my exercise recommendations based on the most recent research, but it is often difficult to decipher the details of the intervention from the research protocol. It would be great to have detailed evidence-based programs to recommend. |
|  | the feasibility of accomplishing this task is my question given the different provincial support networks and governing bodies |
|  | I think this will be very difficult to achieve in the paediatric and Teenage populations due to the smaller numbers and building community practitioners confidence |
|  | Operationalizing this will be quite difficult with independent entities not working within the same healthcare system |
|  | How would it be measured? |
|  | My only comment/question would be whether there it may be more sustainable to have a body to oversee exercise standards for chronic disease survivors/management with cancer being one...maybe a first focus. |
| **Not Actionable** | Same comment as previous |
| **Different Theme** | It would be nice to be assessing which of these types of referrals work best for different types of patients |
| **Round 1 Theme L** | **Details** |
| **Title** | -- |
| **Goals** | Sometimes some perceived benefits may not be measurable or detectable by common testing methods. Expand to include definitions. Current state is vague. |
|  | Some ‘safety checks’ in cancer care reduce availability or delay starting. Where possible, eliminate physician permission for patients that can self-assess. Patients who don’t experience with fitness can maybe have more help determining where they’re starting and what is reasonable. |
|  | I would recommend the development of a standardized auditing function that would be run by an NPO |
|  | Focus on efficacy as lots of research already identifying safety of exercise |
|  | I think there is enough evidence to support the benefits of exercise for cancer survivors. Focus should look towards establishing the programs. Once this is established larger scale studies can be conducted on the benefits of these programs in a real-world setting. This follows the same model as the cardiac rehab approach. |
|  | it is unclear what the outcomes of interest are here and how they will be assessed to provide information on safety |
|  | Change efficacy to effectiveness. I'm assuming these community interventions will reflect the real-world and not some well-controlled environment? If the assessment will be done in the real world then I think effectiveness is the more accurate word. That said, usually you would assess feasibility alongside safety, before moving onto investigate effectiveness. |
|  | Cost-effectiveness as well (if not included in subsequent priority areas) |
|  | add cost; add health economists |
|  | This one is complex and needs to likely be more comprehensively thought though. Considerations to program evaluation should be explored and evaluation models might be presented to ensure that real community-based programs understand how to 1) partner with researchers, 2) set up their program for evaluation in the future, 3) perform comprehensive data collection and 4) perform a rigorous evaluation. These settings are highly heterogeneous, which makes the research tricky (but still needed). Most community programs have no idea how to set up assessments in a way that could be successfully evaluated in the future and this is where I think the biggest gap is currently. Research partnerships will be key. |
|  | Pragmatic outcome evaluations and process evaluations should be embedded into research practice |
|  | Safety of the programmes will be denoted by some of the other priorities i.e. training needs for things like fracture risk and collapsed vertebra. Risks may differ more in some patient groups than others? So, I perceive you would have to try and test the training and include some of the risk assessments in the trials designs for these studies |
|  | Safety must be a given. In addition to evaluating outcomes I think it would be key to evaluating cost/benefit... particularly if the cost is being assumed by the health care system/insurance coverage. |
|  | How would you assess safety of the intervention? |
|  | Consider clinical effectiveness (in place of efficacy) for outcomes. Due to the evidence supporting evidence and barriers to dissemination pragmatic trials and practice-based evidence may be most pertinent. |
|  | Shift focus from efficacy to effectiveness and implementation outcomes. If we want to move more into practice, the HOW to implement and how effective in real world contexts is essential. |
|  | Safety less of concern than how to ensure patients with complex needs have high support levels |
|  | good triage is needed for whom should be referred to which program, and at what timepoint |
|  | if feel transition to community programs would require more than post treatment exercise advices as many would have other chronic conditions that need QEP to address. |
|  | It has to be run by professional. |
| **Stakeholders** | CTRS |
|  | clarify expertise of "researchers" |
|  | NPO |
| **Impacts** | -- |
| **General** | All that is built should be evidence-based. Yes, obviously safety monitoring should be built into programs. But don't think this is its own issue. it's part of building safe and beneficial programs! |
|  | This will follow once there is an understanding from the medical community about who to refer, when and where. |
|  | Not sure this is essential. Studies and existing community-based program have demonstrated safety and efficacy |
|  | is this goal even achievable? |
|  | if this does not include cost-effectiveness then I would rank this as not very important; |
|  | Has this not already been done in other settings? |
|  | Already have lots of evidence of efficacy and safety |
|  | Again, in the ideal world this would be the standard. I'm having a difficult time envisioning how this is put into play regionally or nationally but welcome the concept development. |
|  | Can look to other programs - e.g. Cardiac Rehabilitation - for partnerships/possible structure. No need to reinvent the wheel |
|  | Feels a little vague. I think there needs to be another point on which groups of patients this is safe and suitable for- risk/triage. |
|  | If you push too hard and move to fast there could be negative sequalae |
|  | Question of whether the efficacy has been sufficiently studied yet. There is already so much evidence, is this the priority? Are biological explanations what is required now to secure medical community buy-in? |
|  | this research will need to address the fact that the sickest/ most deconditioned patients do not volunteer for studies… So, our results on safety are biased by healthy samples |
|  | The difficulty in doing this for such a diverse group of patients with such a diverse range of exercises. I feel the aim should be for people to go back to activities they were doing before and establishing safety for all of these could be difficult. Also, if you are saying something is safe and it goes wrong for one person how liable are you? |
|  | Just wondering when the age of a patient becomes limiting to the exercise output. Regardless of outcomes, is there a point when exercise can potentially do more harm than good for an older patient? Like, joints, back, muscle etc. |
| **Not Actionable** | Our continued studies are supervised and community-based programs. |
|  | stated above, not a good use of research resources as the data is already available |
| **Different Theme** | -- |
| **Round 1 Theme M** | **Details** |
| **Title** | -- |
| **Goals** | I think it might be beneficial to include resources. For example, maybe they don't have to see a specialized individual but have access to see if they can exercise outside of the normal gym. Maybe play league sports, but are too scared etc. Maybe guidance to feasibility of sport would also be beneficial to the survivor, especially if they were highly active prior to diagnosis. |
|  | Exercise consultation for 30-60 min to ensure that each person's programme is tailored for adherence and potential independence |
|  | By 'dynamic intervention support opportunities' I presume advanced training methods such as HIIT or TRX? Correct? |
|  | This is a challenging one. It's difficult to diversify approaches when attempting to standardize methods/routines. These patients are best served with an individualized assessment for exercise recommendations. |
|  | To me the goals are not clearly linked to the impact |
|  | Goal 1 seems a little repetitive, maybe this priority is more about goal 2 (implementation strategies)? |
|  | Implementation strategies are not things like mode of exercise. These are components of the interventions/programs. |
|  | again, think this is captured in earlier points/priorities. Diversity should be part of, not separate, to what is built. Consider rural/remote as primary issue for outreach. |
|  | goal 2 - could add frequency of contact with exercise professionals and maybe look at differences between modes of contact e.g. face to face, phone call, email, text. Does more contact improve exercise program compliance and therefore assist in improving functional outcomes? maybe not something to necessarily change but could be interesting to look at |
|  | Seems like it could be combined with the first priority on messaging - the goal is the same |
|  | Add importance of improving exercise self-efficacy of patients as this appears to be the key to exercise maintenance from the exercise oncology research I've been reading/involved with |
|  | Make exercising fun, accessible, and inexpensive. Provide trained experts and benchmarks. Provide a supportive community. |
|  | Consider including language that describes non-cancer specific programs. For the majority of breast and prostate cancer survivors, once treatment is competed, over time they will return to more normal physical functioning and have less cancer-specific needs. These people need to be accommodated in this priority. |
|  | Incorporate behaviour change intervention as part of this |
|  | Feels like this would be a prime opportunity to build behavioral science into the mix |
|  | Are you looking to develop a program or existing programs? I am not sure how the goals and impact relate |
|  | I find this confusing. The first aim looks at exercise engagement strategies while the second deals with components of an exercise trial that you would adjust when trying to establish the appropriate dose (in efficacy trials). I would like this be clearer, I think what it is getting at is looking at innovative ways to implement exercise referral programmes and encourage patient engagement. |
|  | Not really sure what this objective is about |
| **Stakeholders** | clarify expertise of "researchers" |
|  | Do not know what LTW/LTD; are you including dietitians, health educators, etc.? |
|  | CTRS |
| **Impacts** | To me the goals are not clearly linked to the impact |
|  | Impact: 3. Establishment of a comprehensive base of exercise support services that meet the patients' geography, demographics & medical status needs |
| **General** | I think the environment is somewhat supportive already. The greatest challenge is creating behavioral change that leads to using resources and meeting the recommended guidelines. |
|  | How do you plan to facilitate exercise engagement in kids (e.g., through play), given that exercise itself may not be something a 5-year-old may be interested in? How would you make exercise attractive for the younger populations? |
|  | What considerations for patients? i.e. cost, time |
|  | Could be combined with research priority #1 perhaps? both centered around engagement |
|  | This seems to be a broader (umbrella) research priority that could encompass some of the earlier priorities around messaging and communication strategies |
|  | This one might go along with Research Priority 13. Our study/program involves a maintenance group that comes back for continued supervised exercise support. We try to teach the importance of moderate level intensity exercise and target heart rate rages (per individual). We use walking/cardio machines and circuit training strength training. |
| **Not Actionable** | The other research priorities must come first. |
|  | The only reason I rank this as important vs very important is that I see a lack of exercise services to begin with for this population so just establishing a baseline is needed before diversity in my opinion |
|  | It feels like we have the most research in this area already |
|  | make this goal number 1 |
| **Different Theme** | -- |
| **Round 1 Theme N** | **Details** |
| **Title** | the title and the goals of this research proposal are not clear |
|  | title and goals are not clear |
| **Goals** | Two very different levels - PROs vs economics. I think eco is a huge issue for the system. But patient-centred care is at the heart of the system. So, balancing these two is essential. I think moving PROs to real-world assessment (not our research validated scales) should be a priority (i.e., single item measures of impact!) |
|  | Supervised programs and consistent funding and infrastructure are essential. |
|  | the economic return on investment needs to be at the patient level too-- not just looking at payers. patients give up time, money and have other "opportunity costs" |
|  | Ensure that research is both quantitative and qualitative. Patients are interested in quality of life as much as progression free survival |
|  | the title and the goals of this research proposal are not clear |
|  | Yes, we still need to establish what outcomes are most important for patients of different cancers at different stages of treatment and beyond. |
|  | Ultimately this will be the goal, but I believe one must "prove" the benefit before health care institutions will fully support these programs |
|  | This is a lofty goal that will remain aspirational unless it is associated with high value within the system or at least a break-even billing mechanism |
|  | title and goals are not clear |
|  | Examples here may be helpful - of particular interest, I think a well-designed rigorous and well-powered study that showed earlier return to work after adjuvant therapy for breast or colon cancer (Stage 2-3) could lead to the desired outcomes. |
|  | This seems a little confused in its focus, economic evaluation is really important but economic priorities of organisations multi-nationally will be difficult to gain consensus over. Evaluation of economic impact and benefit would be a more important priority. Patient outcomes are equally very important, but I feel this priority doesn’t capture it and there doesn’t seem to be any that seem to do this? |
| **Stakeholders** | NPOs as stakeholders |
|  | CTRS |
|  | clarify expertise of "researchers" |
| **Impacts** | A bursary to help fund the research and programming |
|  | Need to be third-party covered to be sustainable in my opinion |
|  | This is critical for sustainability and to bid for more funds |
| **General** | I think this is a first priority, determining who will cover the cost. |
|  | I think this needs to come under a bigger cancer rehab umbrella. In addition, many cancer survivors do well and do not need anything, so we need to target those who need the services the most. It is not realistic to think we could offer tailored exercise programs to all cancer patients. |
|  | This will vary by country and institution. I think it's worth developing a consortium of professionals and researchers in this space from a variety of countries to discuss challenges and success in their respective countries so we can learn from each other. Importantly, I think these efforts need to be open and inclusive to all, lead by a core group but needs to allow for ECR's and others input. These efforts, internationally, will be monumental and we need as many hands sharing the work as possible. Giving different people key roles to lead different arms, initiatives and development of resources will be critical. This can bottleneck pretty quickly if it's the same 20 people leading all projects and initiatives. If we care about this as much as we say we do, let's build it together |
|  | I think the highest impact this would have is in justifying the addition of QEPs to the healthcare system if we can unequivocally demonstrate a cost-benefit of hiring QEPs in the health system |
|  | There seem to be a number of very distinct steps here - what is the research priority and what aspects are expected products/outputs from a new line of research vs. existing literature |
|  | This is one of the largest barriers that we see in clinical practice, having support for this type of infrastructure would make a large impact |
|  | Given budgetary restrictions, the issue of buy-in to program delivery within the health care system is often resource limitations. The business case/economic case in addition to patient/survivor benefit must be made to get buy in and commitment to invest. |
|  | In my opinion this is the most important priority by far. Many of the previous priorities are moot unless we can achieve this. |
|  | Achieving this goal would certainly make achieving most of the previous goals more easily justified. |
|  | This is by far the most important component for standardizing exercise in cancer and more broadly exercise physiology. This also should include patient triage processes because they currently do not exist. |
|  | Compared to the cost of traditional medical care, an exercise program is relatively inexpensive yet highly effective. Thank you for your efforts to try to tip the scales in terms of promoting exercise as a healthcare strategy. |
|  | This is the most challenging question - cancer care is expensive and the economic argument for supportive care may be key to moving this agenda forward |
|  | I think this is vital for future sustainability of QEP services, if we can display there is economic benefit (e.g. reduced hospitalisations, co-morbidity, faster return to work etc.) it is of great benefit to individual and community |
|  | paying for is necessary (& critical) but not sufficient; e.g., cardiac rehab copays in the US can be quite high so we could expect the same thing if the same paradigm is used for cancer rehab |
|  | In my opinion, this is the most important goal that is needed to be attained in order to implement the other goals on a larger scale. Is there any evidence from federal governments of any country to be willing to support this type of initiative? If so, how is this done? |
|  | May I be included as part of study. I am a breast cancer survivor diagnosed two weeks before my 33rd birthday. I have worked at, my whole professional career, at Oregon State Hospital in Salem, OR, USA. I started as a therapist. Now I am the CTRS Director. Please research my profession at www.nctrc.org |
| **Not Actionable** | I think this is very important |
|  | Will vary by country |
|  | Would love to see this approach applied consistently across exercise rehabilitation/chronic disease management programs of all types in all provinces... so challenging |
|  | I would rank this as very very important! |
|  | I feel this is a huge priority! |
|  | I think this is a great issue. If funding was available, I think more people would be interested to delve into the field. |
|  | This would be amazing! |
|  | Important to engage the survivor and caregiver in this process |
| **Different Theme** | -- |

Theme Changes Across Survey Rounds 1-3

**Theme identifiers**

***Please note, the overall in Online Supplement 3 are based on the survey round 3 alphabetical labels.***

| **Theme A** |
| --- |

| **Round #** | **Theme ‘A’ Title** |
| --- | --- |
| Round 1 | Messaging strategies to support cancer survivors’ exercise engagement |
| Round 2 | Communication strategies to support cancer survivors’ exercise engagement throughout the survivorship trajectory |
| Round 3 | Enhancing communication strategies to increase cancer survivors’ exercise engagement throughout the survivorship trajectory |

| **Round #** | **Theme ‘A’ Goals** |
| --- | --- |
| Round 1 | 1. Establish the survivor-identified messaging priorities to motivate & support exercise-related behaviour change 2. Develop recommendations for effective exercise-related communication with survivors |
| Round 2 | 1. **Research / Dissemination:** Establish & promote demographic & region-specific survivor-identified communication preferences & content-based messaging priorities to:    1. increased survivors’ & supporters’ awareness of exercise-related risks, benefits, & exercise support services    2. motivate & support cancer survivors to change their exercise-related behaviour throughout the survivorship trajectory 2. **Research / Implementation:** Develop & implement recommendations for effective, evidence-based exercise-related communication approaches with survivors & supporters |
| Round 3 | 1. **Research / Dissemination:** Establish & promote demographic-, cultural-, language- & region-specific survivor-identified communication content & approaches to:    1. increase survivors’ & supporters’ awareness of exercise benefits, risks, and support services    2. motivate & support cancer survivors to change their exercise behaviour throughout the survivorship trajectory 2. **Research / Implementation:** Improve the quality of exercise communication for specific cancer survivor & supporter groups by developing & implementing recommendations for effective, evidence-based exercise communication content & approaches |

| **Round #** | **Theme ‘A’ Stakeholders** |
| --- | --- |
| Round 1 | Community partners; physicians & HCPs; QEPs; researchers; survivors & care providers |
| Round 2 | Community partners; educators; HCPs; industry; QEPs; researchers; survivors & supporters |
| Round 3 | Community partners; educators; HCPs; industry; QEPs; researchers; survivors & supporters |

| **Round #** | **Theme ‘A’ Impacts** |
| --- | --- |
| Round 1 | 1. Improved practitioner understanding of survivor preferences related to exercise counselling 2. Improved effectiveness of exercise-related counselling leading to increased exercise engagement & benefit for cancer survivors |
| Round 2 | 1. Improved survivor- & supporter-awareness of the risks, benefits, & support services 2. Improved practitioner understanding of survivor & supporter needs & preferences related to exercise counselling to optimize exercise engagement & benefits for cancer survivors across the survivorship trajectory |
| Round 3 | 1. Improved survivor- & supporter-awareness of exercise benefits, risks & support services 2. Improved HCP understanding of survivor & supporter-preferred exercise communication content & approaches to optimize exercise engagement & benefits for cancer survivors across the survivorship trajectory |

| **Theme B** |
| --- |

| **Round #** | **Theme ‘B’ Title** |
| --- | --- |
| Round 1 | Exercise oncology education models for oncologists & primary care teams |
| Round 2 | Evidence-based exercise oncology education models for diverse HCPs working with cancer survivors |
| Round 3 | Developing & promoting evidence-based exercise oncology education models for HCPs working with cancer survivors |

| **Round #** | **Theme ‘B’ Goals** |
| --- | --- |
| Round 1 | 1. Increase awareness & improve attitudes and beliefs towards the benefits of exercise for cancer survivors 2. Increase oncologists’ exercise-related competence & capacity to effectively educate patients |
| Round 2 | 1. **Research / Dissemination:** Increase awareness & knowledge to improve attitudes & beliefs of HCPs towards the benefits of exercise for cancer survivors through various approaches 2. **Research / Dissemination / Implementation:** Develop, promote, & embed resources & tools within medical & community settings to facilitate exercise-related discussions between HCPs & survivors |
| Round 3 | 1. **Research / Dissemination:** Increase awareness & knowledge of HCP’s on the benefits of exercise for cancer survivors through varying educational approaches 2. **Research / Dissemination / Implementation:** Increase exercise-related communication between HCPs & survivors by developing, promoting, & embedding exercise communication resources & tools within medical & community care settings |

| **Round #** | **Theme ‘B’ Stakeholders** |
| --- | --- |
| Round 1 | Administrators (institution); community partners; physicians & HCPs; professional associations & societies; QEPs; researchers; survivors & care providers; unions |
| Round 2 | Administrators (healthcare institutions); community partners & practitioners; educators; HCPs; policy makers; professional associations & societies; QEPs; researchers; survivors & supporters; unions |
| Round 3 | Administrators (healthcare institutions); community partners & practitioners; educators; HCPs; policy makers; professional associations & societies; QEPs; researchers; survivors & supporters; unions |

| **Round #** | **Theme ‘B’ Impacts** |
| --- | --- |
| Round 1 | 1. Increased rates of oncologist exercise endorsement (patient-level) & exercise program referrals (institution-/community-level) 2. Increased oncologists’ motivation & proficiency in exercise counselling 3. Enhanced institutional culture of health and wellness |
| Round 2 | 1. Increased HCPs’ motivation & proficiency in discussing the risks, benefits & strength of recommendations for exercise with survivors 2. Increased rates of appropriate exercise endorsement (patient-level) & exercise program referrals (medically supervised- & community-based levels) by HCPs |
| Round 3 | 1. Increased HCPs’ motivation & proficiency to discuss the benefits, risk, & guidelines for exercise with survivors 2. Increased rates of appropriate exercise endorsement (patient-level) & exercise program referrals (medically supervised- & community-based levels) by HCPs |

| **Theme C** |
| --- |

| **Round #** | **Theme ‘C’ Title** |
| --- | --- |
| Round 1 | Standardized exercise oncology training for diverse exercise professionals across various training environments |
| Round 2 | Standards for exercise oncology training for diverse qualified exercise professionals (QEPs) across various training environments |
| Round 3 | Establishing exercise oncology training standards for qualified exercise professionals (QEPs) across training environments |

| **Round #** | **Theme ‘C’ Goals** |
| --- | --- |
| Round 1 | 1. Increase the number of QEPs 2. Promote & support a standardized exercise oncology curriculum across diverse undergraduate & graduate-level training programs 3. Promote & support the development & standardization of accessible community-based continuing education training opportunities in exercise oncology for diverse exercise professionals 4. Clearly delineate scope of practice across professions working in exercise oncology |
| Round 2 | 1. **Research:** Develop education standards for foundational & continuing professional & community-based training opportunities in exercise oncology that are accessible to diverse (e.g. geographically, demographically) QEPs 2. **Dissemination:** Clearly delineate & increase awareness of the boundaries & overlap of competencies / scope of practice for QEPs (i.e. allied health professionals ⟶ community practitioners) working in exercise oncology 3. **Dissemination / Uptake / Implementation:** Promote & support standards for evidence-based exercise oncology curriculum across various community, college, undergraduate & graduate-level training programs |
| Round 3 | 1. **Research:** Develop education standards for (1) foundational & (2) continuing professional & community-based training opportunities in exercise oncology that are accessible to diverse QEPs (e.g. geographically, demographically, academically) 2. **Dissemination:** Define & increase awareness of the boundaries & overlap of competencies / scope of practice for QEPs (i.e. allied health professionals ⟶ community practitioners) working in exercise oncology 3. **Dissemination / Uptake / Implementation:** Promote & support the adoption of standards for evidence-based exercise oncology curriculums across training settings (e.g. community, professional association, college, undergraduate & graduate programs) |

| **Round #** | **Theme ‘C’ Stakeholders** |
| --- | --- |
| Round 1 | Administrators (university faculty); community partners; educators (university or community-based); physicians & HCPs; professional organizations; QEPs; researchers |
| Round 2 | Administrators (healthcare institutions); community partners & practitioners; educators; HCPs; professional associations & societies; QEPs; researchers; survivors & supporters |
| Round 3 | Administrators (healthcare institutions); community partners & practitioners; educators; HCPs; professional associations & societies; QEPs; researchers; survivors & supporters |

| **Round #** | **Theme ‘C’ Impacts** |
| --- | --- |
| Round 1 | 1. Increased accessibility to QEPs within urban & rural communities 2. Increased knowledge & awareness of college & university graduates on the unique exercise needs & considerations for cancer survivors 3. Increased opportunities for quality training to acquire specialized knowledge & skills related to exercise for cancer survivors 4. Improved interprofessional communication & collaboration between QEPs to optimize care |
| Round 2 | 1. Increased number of diverse QEPs to provide evidence-based exercise & rehabilitation support to survivors across medical, clinical & community settings 2. Increased opportunities for diverse QEPs to acquire appropriate knowledge & skills to support collaborative oncology-specific assessment, exercise prescription, & physical activity promotion for cancer survivors 3. Improved interprofessional communication & collaboration between QEPs to optimize care |
| Round 3 | 1. Increased number of QEPs providing evidence-based exercise & rehabilitation support to survivors across medical, clinical & community settings 2. Increased opportunities for QEPs to acquire appropriate knowledge & skills to support coordinated multidisciplinary exercise services (e.g., exercise counselling, screening, testing, & prescription) for cancer survivors 3. Improved interprofessional communication & collaboration between QEPs to optimize care |

| **Theme D** |
| --- |

| **Round #** | **Theme ‘D’ Title** |
| --- | --- |
| Round 1  (Theme D) | Novel approaches for supporting hard to reach and understudied cancer populations with exercise |
| Round 1  (Theme E) | Technology-based exercise support strategies for diverse demographic and geographic communities of cancer survivors |
| Round 2  Combined | Technology-based exercise support strategies across demographic & geographic communities of cancer survivors |
| Round 3 | Enhancing technology-based strategies to improve the delivery of exercise support to demographically-, culturally-, & geographically-diverse communities of cancer survivors |

| **Round #** | **Theme ‘D’ Goals** |
| --- | --- |
| Round 1  (Theme D) | 1. Identify most effective engagement strategies for hard to reach and understudied cancer populations 2. Establish the exercise support needs of hard to reach & understudied cancer populations 3. Develop & evaluate the effects of novel intervention strategies to meet the unique needs of these populations 4. Support the adoption of novel exercise implementation strategies for these populations at the community level |
| Round 1  (Theme E) | 1. Identify most effective technology-based exercise support strategies for diverse cancer populations 2. Develop & evaluate evidence-based exercise support strategies using technology to meet the needs of diverse cancer survivors 3. Provide increased opportunities & modalities for cancer survivors to engage in exercise using technology via self-directed or QEP-supported approaches |
| Round 2  Combined | 1. **Research:** Identify the technology-based needs of diverse cancer survivor populations to enable & improve all aspects of exercise support 2. **Research:** Develop & evaluate the feasibility, safety, efficacy, effectiveness, & sustainability of technology-based exercise support strategies to meet the needs of diverse cancer survivors across the survivorship trajectory 3. **Dissemination / Uptake / Implementation:** Promote & support increased opportunities for the integration of technologies within self-directed or supervised approaches for exercise support in diverse cancer survivor populations |
| Round 3 | 1. **Research:** Identify the technology-based needs of diverse cancer survivor populations to enable & improve all aspects of exercise support 2. **Research:** Develop & evaluate the feasibility, safety, efficacy, effectiveness, & sustainability of technology-based exercise support strategies to meet the needs of diverse cancer survivors across the survivorship trajectory 3. **Dissemination / Uptake / Implementation:** Promote & support increased opportunities for the integration of technologies within self-directed & supervised exercise support settings for diverse cancer survivor populations |

| **Round #** | **Theme ‘D’ Stakeholders** |
| --- | --- |
| Round 1  (Theme D) | Administrators (institution); community partners; extended health care providers (e.g., insurance, RTW/LTD); physicians & HCPs; policy makers; QEPs; researchers; survivors & care providers |
| Round 1  (Theme E) | Administrators (institution); community partners; extended health care providers (e.g., insurance, RTW/LTD); industry (e.g., technology companies); physicians & HCPs; policy makers; QEPs; researchers; survivors & care providers |
| Round 2  Combined | Administrators (healthcare institutions); community partners & practitioners; HCPs; industry (e.g. technology companies); policy makers; QEPs; researchers; survivors & supporters; third-party healthcare insurers |
| Round 3 | Administrators (healthcare institutions); community partners & practitioners; HCPs; industry (e.g. technology companies); policy makers; QEPs; researchers; survivors & supporters; third-party healthcare insurers |

| **Round #** | **Theme ‘D’ Impacts** |
| --- | --- |
| Round 1  (Theme D) | 1. Increased awareness & understanding of the unique exercise-related support needs of hard to reach & understudied cancer populations 2. Determine the most effective exercise support strategies to improve behavioural & clinical outcomes in hard to reach & understudied cancer populations 3. Improved accessibility to engagement within exercise support services for these populations leading to optimize health & survival |
| Round 1  (Theme E) | 1. Increased availability of technology-based exercise support services for cancer survivors 2. Decreased barriers to exercise engagement (e.g., time, cost, program proximity) for diverse cancer survivor groups 3. Increased reach of evidence-based, high-quality programs for all cancer survivors in order to increase exercise-related knowledge & behaviour 4. Increased capacity of QEPs & community exercise programs to meet the needs of diverse cancer populations |
| Round 2  Combined | 1. Increased accessibility & awareness of feasible, safe, efficacious, effective, & sustainable technology-based exercise support systems & services for cancer survivors 2. Decreased barriers to exercise engagement (e.g. time, cost, program proximity) for cancer survivors 3. Increased reach of evidence-based, high-quality interventions for all cancer survivors in order to promote equity in exercise support across the survivorship trajectory 4. Increased awareness & capacity of QEPs & community exercise programs to meet the needs of diverse cancer populations using technology |
| Round 3 | 1. Increased accessibility & awareness of feasible, safe, efficacious, effective, & sustainable technology-based exercise support systems & services for cancer survivors 2. Decreased barriers to exercise engagement (e.g. time, cost, program proximity) for cancer survivors 3. Increased reach of evidence-based, high-quality interventions for all cancer survivors in order to promote equity in exercise support across the survivorship trajectory 4. Increased awareness & capacity of QEPs & community exercise programs to meet the needs of diverse cancer populations using technology |

| **Theme E** |
| --- |

| **Round #** | **Theme ‘E’ Title** |
| --- | --- |
| Round 1 | Qualified Exercise Professional (QEP) integration into primary cancer care teams |
| Round 2 | Qualified Exercise Professional (QEP) integration into primary cancer care teams |
| Round 3 | Integrating Qualified Exercise Professionals (QEPs) into primary cancer care teams |

| **Round #** | **Theme ‘E’ Goals** |
| --- | --- |
| Round 1 | 1. Define the role of QEPs in cancer care 2. Evaluate the system-, team- & patient-level benefits of QEP inclusion in primary cancer care team |
| Round 2 | 1. **Research:** Establish the needs & define the role / scope of practice for QEPs within primary cancer care teams globally 2. **Research:** Evaluate the system-, team- & patient-level benefits, & cost-effectiveness of QEP inclusion within primary cancer care teams 3. **Dissemination / Uptake / Implementation:** Promote the implementation of effective integration strategies for QEPs within primary cancer care teams |
| Round 3 | 1. **Research:** Establish the needs & define the role / scope of practice for QEPs within primary cancer care teams across geographical regions and healthcare settings 2. **Research:** Evaluate the system-, team- & patient-level benefits, & cost-effectiveness of QEP inclusion within primary cancer care teams 3. **Dissemination / Uptake / Implementation:** Promote the implementation of effective integration strategies for QEPs within primary cancer care teams |

| **Round #** | **Theme ‘E’ Stakeholders** |
| --- | --- |
| Round 1 | Administrators (institution); community partners; physicians & HCPs; professional organizations & societies; QEPs; researchers; survivors & care providers |
| Round 2 | Administrators (healthcare institutions); community partners & practitioners; HCPs; policy makers; professional organizations & societies; QEPs; researchers; survivors & supporters; third-party healthcare insurers |
| Round 3 | Administrators (healthcare institutions); community partners & practitioners; HCPs; policy makers; professional organizations & societies; QEPs; researchers; survivors & supporters; third-party healthcare insurers |

| **Round #** | **Theme ‘E’ Impacts** |
| --- | --- |
| Round 1 | 1. Increased awareness & use of QEP expertise 2. Alleviated burden of exercise counselling from other primary care team members 3. Increased access & quality of exercise-related patient education throughout the cancer trajectory 4. Improved exercise screening & assessment leading to more appropriate patient triage & referrals & efficiency of use of hospital- and community-based resources & support 5. Improved short- & long-term patient outcomes leading to reduced health care costs & resource utilization |
| Round 2 | 1. Increased awareness & use of QEP expertise within primary cancer care teams 2. Alleviated burden of exercise counselling from other primary cancer care team members 3. Increased accessibility & quality of exercise-related patient education throughout the cancer trajectory 4. Improved exercise screening & assessment leading to more appropriate patient triage & referrals & efficiency of use of medically supervised & community-based resources & support 5. Improved short- & long-term patient outcomes leading to reduced Healthcare costs & resource utilization |
| Round 3 | 1. Increased awareness & use of QEP expertise within primary cancer care teams 2. Alleviated burden of exercise counselling from other primary cancer care team members 3. Increased accessibility & quality of exercise-related patient education throughout the cancer trajectory 4. Improved exercise screening & assessment leading to more appropriate patient triage & referrals & efficiency of use of medically supervised & community-based resources & support 5. Improved short- & long-term patient outcomes leading to reduced healthcare costs & resource utilization |

| **Theme F** |
| --- |

| **Round #** | **Theme ‘F’ Title** |
| --- | --- |
| Round 1 | Referral mechanisms to clinical- and community-based cancer exercise programs |
| Round 2 | Referral pathways & mechanisms for cancer survivors into medically-supervised & community-based cancer exercise programs |
| Round 3 | Establishing resources for referring cancer survivors between medical- & community-based cancer exercise services |

| **Round #** | **Theme ‘F’ Goals** |
| --- | --- |
| Round 1 | 1. Increase overall volume of survivor referrals to cancer exercise programs 2. Identify available cancer exercise services within a region 3. Create a standardized tool/template to communicate relevant information between primary care teams & QEPs |
| Round 2 | 1. **Research:** Evaluate needs- / risk-based assessment & communication tools with corresponding pathways to improve the appropriateness & efficiency of referrals to clinical & community cancer exercise programs 2. **Research / Dissemination:** Identify, describe, organize, & promote cancer exercise programs within a region for self- & HCP referral 3. **Dissemination / Uptake / Implementation:** Implement & support the use of appropriate tools & templates to communicate relevant information between HCPs & QEPs in clinical & community-based cancer exercise programs |
| Round 3 | 1. **Research:** Evaluate needs- & risk-based assessment & communication tools, with corresponding referral processes, to improve the appropriateness & efficiency of self- & practitioner-referrals between clinical & community cancer exercise services 2. **Research / Dissemination:** Identify, describe, organize, & promote cancer exercise services within a region for self- & HCP referral 3. **Dissemination / Uptake / Implementation:** Implement & support the use of appropriate resources (i.e. tools & systems) by HCPs & QEPs to improve communication between clinical & community-based cancer exercise services |

| **Round #** | **Theme ‘F’ Stakeholders** |
| --- | --- |
| Round 1 | Community partners; physicians & HCPs; QEPs; researchers; survivors & care providers |
| Round 2 | Community partners; industry; HCPs; QEPs; researchers; survivors & supporters |
| Round 3 | Community partners; industry; HCPs; QEPs; researchers; survivors & supporters |

| **Round #** | **Theme ‘F’ Impacts** |
| --- | --- |
| Round 1 | 1. Increased number of people exercising 2. Increased awareness of local cancer exercise resources for primary care teams 3. Increased effectiveness of communication between primary care team & QEPs leading to improved risk management |
| Round 2 | 1. Increased awareness & appropriateness of self- & HCP-referrals for cancer survivors to local cancer exercise resources 2. Established pathways for efficient & appropriate referrals to clinical- & community-based cancer exercise programs 3. Improved communication mechanisms between HCPs & QEPs in clinical- & community-based cancer exercise programs leading to more effective case management |
| Round 3 | 1. Increased awareness & appropriateness of self- & HCP-referrals of cancer survivors to cancer exercise services 2. Established processes for efficient & appropriate referrals between clinical- & community-based cancer exercise services 3. Improved communication between HCPs & QEPs in clinical- & community-based cancer exercise services leading to more effective case management |

| **Theme G** |
| --- |

| **Round #** | **Theme ‘G’ Title** |
| --- | --- |
| Round 1  (Theme H) | Cancer exercise program availability & accessibility |
| Round 1  (Theme K) | Standardized community cancer exercise programming |
| Round 2 | Accessibility of medically-supervised & community-based cancer exercise programs & support services to meet the needs of diverse groups of cancer survivors |
| Round 3 | Improving accessibility of medically-supervised & community-based cancer exercise support services for diverse groups of cancer survivors |

| **Round #** | **Theme ‘G’ Goals** |
| --- | --- |
| Round 1  (Theme H) | 1. Increase availability & accessibility of exercise programs for all cancer survivors 2. Establish infrastructure (physical & web-based) & financial support for new & existing programs |
| Round 1  (Theme K) | 1. Standardize community exercise programming parameters:    1. Survivor screening & testing    2. Intervention delivery    3. Outcome collection & reporting |
| Round 2 | 1. **Research:** Identify the exercise support needs, barriers, & preferred engagement strategies for (a) hard to reach & (b) understudied cancer populations globally 2. **Research / Dissemination:** Leverage existing & establish novel infrastructure (physical & web-based) to create, evaluate, & promote accessible (i.e. available, affordable, acceptable) & equitable opportunities for survivors to engage in evidence-based cancer exercise programs & services 3. **Uptake / Implementation:** Establish new & expand existing funding models to support survivors, QEPs, & community partners to increase accessibility of evidence-based cancer exercise programs & services |
| Round 3 | 1. **Research:** Identify the exercise support needs, barriers, & preferred engagement strategies for (a) hard to reach & (b) understudied cancer populations globally 2. **Research / Dissemination:** Leverage existing & establish novel infrastructure (physical & virtual) to create, evaluate, & promote accessible & equitable opportunities for survivors to engage with evidence-based cancer exercise support services 3. **Uptake / Implementation:** Establish new & expand existing funding models to support survivors, QEPs, & community partners to increase accessibility of & equitable access to evidence-based cancer exercise support services |

| **Round #** | **Theme ‘G’ Stakeholders** |
| --- | --- |
| Round 1  (Theme H) | Administrators (institution); community partners; extended health care providers (e.g., insurance, RTW/LTD); physicians & HCPs; QEPs; researchers; survivors & care providers |
| Round 1  (Theme K) | Administrators (institution); community partners; extended health care providers (e.g., insurance, RTW/LTD); physicians & HCPs; QEPs; researchers; survivors & care providers |
| Round 2 | Administrators (institution); community partners & practitioners; HCPs; policy makers; QEPs; researchers; survivors & supporters; third-party healthcare insurers |
| Round 3 | Administrators (institution); community partners & practitioners; HCPs; policy makers; QEPs; researchers; survivors & supporters; third-party healthcare insurers |

| **Round #** | **Theme ‘G’ Impacts** |
| --- | --- |
| Round 1  (Theme H) | 1. Increased availability of evidence-based exercise support services for cancer survivors 2. Equitable access to exercise services independent of geography, demographics & medical status |
| Round 1  (Theme K) | 1. Standardized exercise support services for cancer survivors 2. Increased effectiveness & safety of community-based programs 3. Robust assessments of short- & long-term outcomes across community-based programs 4. Justification for the development of a governing body/organization to oversee & uphold cancer exercise programming standards |
| Round 2 | 1. Increased awareness & understanding of the unique exercise-related support needs, barriers, & preferred engagement strategies of hard to reach & understudied cancer populations globally 2. Increased accessibility of appropriate evidence-based exercise support services for cancer survivors across the survivorship trajectory to improve behavioural & clinical outcomes 3. Equitable access to exercise programs & support services for cancer survivors independent of geography, demographics & medical status to optimize health & survival |
| Round 3 | 1. Increased awareness & understanding of the unique exercise-related support needs, barriers, & preferred engagement strategies of hard to reach & understudied cancer populations globally 2. Improved behavioural & clinical outcomes via increased accessibility of appropriate evidence-based exercise support services for cancer survivors across the survivorship trajectory independent of geography, demographics & medical status 3. Greater sustainability of accessible evidence-based exercise support services for all cancer survivors |

| **Theme H** |
| --- |

| **Round #** | **Theme ‘H’ Title** |
| --- | --- |
| Round 1 | Exercise oncology resource sharing across academic and community partners |
| Round 2 | Collaborative development & sharing of evidence-based resources across international groups of academic & community partners to support exercise for cancer survivors |
| Round 3 | Developing & sharing of evidence-based resources to support academic & community partners in providing exercise services for cancer survivors |

| **Round #** | **Theme ‘H’ Goals** |
| --- | --- |
| Round 1 | 1. Create mechanisms for developing & sustaining a diverse resource sharing platform (e.g., survivor-facing, program-level, intervention-related) 2. Promote awareness & use of resource sharing platform |
| Round 2 | 1. **Research:** Identification & collaborative production of varied evidence-based resources to support exercise engagement & program delivery for cancer survivors 2. **Research / Uptake / Implementation:** Develop & sustain a platform to share varied developer-acknowledged resources (e.g. survivor-facing, program-level, intervention-related) between academic & community partners 3. **Dissemination / Uptake:** Promote awareness & uptake of resources across settings & use of the resource sharing platform by academic & community partners |
| Round 3 | 1. **Research:** Identification of existing & development of new evidence-based resources to increase exercise engagement & exercise support for cancer survivors across geographic regions and support settings 2. **Research / Uptake / Implementation:** Develop & sustain systems to share & recognize the developers of exercise resources that can be used directly by, or adapted to meet the needs of, academic & community partners to increase exercise engagement & exercise support for cancer survivors 3. **Dissemination / Uptake:** Promote awareness & uptake of information sharing systems & use of appropriate resources by academic & community partners to increase exercise engagement & exercise support for cancer survivors |

| **Round #** | **Theme ‘H’ Stakeholders** |
| --- | --- |
| Round 1 | Community partners; physicians & HCPs; QEPs; researchers; survivors & care providers |
| Round 2 | Administrators (healthcare institutions); community partners & practitioners; HCPs; QEPs; researchers; survivors & supporters |
| Round 3 | Administrators (healthcare institutions); community partners & practitioners; HCPs; QEPs; researchers; survivors & supporters |

| **Round #** | **Theme ‘H’ Impacts** |
| --- | --- |
| Round 1 | 1. Improved content sharing of vetted & high-quality resources to support survivor education, program development, & intervention delivery 2. Increased research dissemination, impact & collaboration between academic & community partners 3. Reduced redundancy, time & costs related to resource development across research & community-based programs |
| Round 2 | 1. Improved content sharing of evidence-based resources across stakeholders to support survivor education, program development, & intervention delivery 2. Increased research dissemination, impact & collaboration between academic & community partners 3. Reduced redundancy, time & costs related to resource development across research, medically supervised & community-based cancer exercise programs |
| Round 3 | 1. Improved content sharing of evidence-based resources across stakeholders to support survivor education, program development, & intervention delivery 2. Increased research dissemination, impact & collaboration between academic & community partners 3. Reduced redundancy, time & costs related to resource development across research, medically supervised & community-based cancer exercise programs |

| **Theme I** |
| --- |

| **Round #** | **Theme ‘I’ Title** |
| --- | --- |
| Round 1 | Cancer survivor transitions from clinical to community exercise settings |
| Round 2 | Cancer survivor transitions across medically-supervised, community-based, & self-directed exercise programs |
| Round 3 | Improving cancer survivor transitions across medically-supervised, community-based, & self-directed exercise settings |

| **Round #** | **Theme ‘I’ Goals** |
| --- | --- |
| Round 1 | 1. Develop strategies to transition survivors from medically supervised or hospital-based exercise programs into community or home-based settings 2. Create a decision-making framework for best practices in survivor transition |
| Round 2 | 1. **Research:** Evaluate the feasibility & effectiveness of strategies to transition survivors from:    1. hospital-based cancer care into community (cancer or non-cancer) or home-based exercise settings;    2. medically supervised cancer exercise programs into community (cancer/non-cancer) or home-based exercise settings; &,    3. community-based cancer exercise programs into non-cancer community or home-based exercise settings 2. **Research / Uptake / Implementation:** Evaluate, establish & implement a dynamic framework for improving survivor risk & needs assessment, exercise education, behavioural support & prescription across support settings for survivors in transition |
| Round 3 | 1. **Research:** Evaluate the feasibility & effectiveness of existing & new strategies to transition cancer survivors between exercise services within different settings (e.g. hospital-, community-, home-based) and with different formats (e.g. supervision levels, populations) 2. **Research / Uptake / Implementation:** Evaluate, establish & implement a framework for improving risk & needs assessments, exercise education, behavioural support, & exercise testing & prescription across support settings for survivors in transition |

| **Round #** | **Theme ‘I’ Stakeholders** |
| --- | --- |
| Round 1 | Administrators (institution); community partners; extended health care providers (e.g., insurance, RTW/LTD); physicians & HCPs; QEPs; researchers; survivors & care providers |
| Round 2 | Administrators (institution); community partners & practitioners; HCPs; QEPs; researchers; survivors & supporters; third-party healthcare insurers |
| Round 3 | Administrators (institution); community partners & practitioners; HCPs; QEPs; researchers; survivors & supporters; third-party healthcare insurers |

| **Round #** | **Theme ‘I’ Impacts** |
| --- | --- |
| Round 1 | 1. Increased survivor confidence & engagement in long-term exercise behaviour 2. Improved support for staff & organizations in managing survivor transitions to community & home-based exercise settings |
| Round 2 | 1. Improved survivor self-efficacy & engagement in sustained exercise behaviour across settings 2. Increased self-efficacy & support for HCPs, QEP, & community partners & practitioners in managing survivor transitions across medically supervised, community-based, & self-directed exercise settings |
| Round 3 | 1. Improved survivor self-efficacy & engagement in sustained exercise behaviour while transitioning across exercise support settings & survivorship phases 2. Increased self-efficacy & support for HCPs, QEPs, & community partners & practitioners in managing survivor transitions between various exercise support settings |

| **Theme J** |
| --- |

| **Round #** | **Theme ‘J’ Title** |
| --- | --- |
| Round 1 | Safety and efficacy of community-based cancer exercise support services |
| Round 2 | Feasibility, safety, efficacy, & effectiveness of community-based cancer exercise support services |
| Round 3 | Establishing the appropriateness & benefits of community-based cancer exercise support services |

| **Round #** | **Theme ‘J’ Goals** |
| --- | --- |
| Round 1 | 1. Confirm the safety of community-based, supervised exercise interventions for cancer survivors 2. Assess the efficacy of community-based, supervised exercise interventions on key short- & long-term outcomes in cancer survivors |
| Round 2 | 1. **Research:** Evaluate the feasibility, safety, efficacy & effectiveness (including cost) of community-based exercise screening, assessment, & intervention practices to optimize risk management & benefits for diverse groups of cancer survivors 2. **Research / Dissemination / Implementation:** Establish & promote minimum standards for short- / long-term data collection & outcome reporting to meet the broad needs of exercise oncology stakeholders (i.e. survivors ⟶ policy makers) 3. **Dissemination / Uptake / Implementation:** Promote the uptake & implementation of the developed best practices & guidelines with demonstrated feasibility, safety, efficacy, & effectiveness for community-based cancer exercise support services |
| Round 3 | 1. **Research:** Evaluate the appropriateness (feasibility, safety, tolerability) & benefits (efficacy & effectiveness (including cost)) of community-based exercise screening, testing, & intervention practices to optimize exercise-related risk management & benefits for diverse groups of cancer survivors 2. **Research / Dissemination / Implementation:** Establish & promote minimum standards for community-level data collection & outcome reporting to meet the broad needs of exercise oncology stakeholders (i.e. survivors ⟶ policy makers) 3. **Dissemination / Uptake / Implementation:** Promote the uptake & adoption of exercise support services that are appropriate & beneficial for diverse groups of cancer survivors with accompanying implementation strategies |

| **Round #** | **Theme ‘J’ Stakeholders** |
| --- | --- |
| Round 1 | Community partners; extended health care providers (e.g., insurance, RTW/LTD); physicians & HCPs; policy makers; QEPs; researchers; survivors & care providers |
| Round 2 | Administrators (healthcare institutions); community partners & practitioners; HCPs; policy makers; QEPs; researchers; survivors & supporters; third-party healthcare insurers |
| Round 3 | Administrators (healthcare institutions); community partners & practitioners; HCPs; policy makers; QEPs; researchers; survivors & supporters; third-party healthcare insurers |

| **Round #** | **Theme ‘J’ Impacts** |
| --- | --- |
| Round 1 | 1. Establishment of a robust evidence base supporting the safety & efficacy of community-based exercise interventions for cancer survivors to support the permanent adoption of these interventions |
| Round 2 | 1. Establishment of a robust evidence base supporting the feasibility, safety, efficacy, & effectiveness of community-based exercise interventions for cancer survivors to support the permanent adoption of exercise as a standard of cancer care 2. Improved rigor of short- & long-term outcome assessments across various community settings to justify exercise becoming a standard of cancer care 3. Improved stakeholder knowledge surrounding the elements of cancer exercise program practice, design & delivery, & outcomes shown to be unsuccessful (e.g. not feasible, unsafe, not efficacious, & not effective) & successful (e.g. feasible, safe, efficacious, & effective) across diverse cancer survivor groups & community settings |
| Round 3 | 1. Establishment of a robust evidence base supporting the appropriateness & benefits of community-based exercise interventions for cancer survivors to support the permanent adoption of exercise as a standard of cancer care 2. Improved rigor of short- & long-term outcome assessments across various community settings to support the permanent adoption of exercise as a standard of cancer care 3. Improved stakeholder knowledge surrounding the elements of cancer exercise program practice, design & delivery, & outcomes shown to be unsuccessful (e.g. not appropriate and/or beneficial) & successful (e.g. appropriate and/or beneficial) across diverse cancer survivor groups & community settings |

| **Theme K** |
| --- |

| **Round #** | **Theme ‘K’ Title** |
| --- | --- |
| Round 1 | Diverse approaches to facilitate exercise engagement in cancer survivors |
| Round 2 | Approaches & resources to facilitate sustained exercise engagement & participation to meet the needs of diverse cancer survivors |
| Round 3 | Optimizing approaches & resources to facilitate sustained exercise behaviour change in cancer survivors |

| **Round #** | **Theme ‘K’ Goals** |
| --- | --- |
| Round 1 | 1. Identify existing & develop novel approaches to support sustained exercise participation in cancer survivors 2. Explore & evaluate implementation strategies for exercise (e.g., mode, timing, supervision type) across various settings & populations |
| Round 2 | 1. **Research / Dissemination:** Identify & promote existing & novel approaches to support exercise engagement & sustained exercise participation to meet the unique needs of individual, & groups of, cancer survivors 2. **Research / Uptake / Implementation:** Explore & evaluate implementation strategies for existing & novel approaches to optimize exercise engagement & ongoing participation across various settings & diverse populations of cancer survivors |
| Round 3 | 1. **Research / Dissemination:** Identify & promote existing & novel approaches to support sustained exercise behaviour change to meet the unique needs of individual, & groups of, cancer survivors 2. **Research / Uptake / Implementation:** Explore & evaluate implementation strategies for existing & novel approaches to optimize sustained exercise behaviour change across different settings & populations of cancer survivors |

| **Round #** | **Theme ‘K’ Stakeholders** |
| --- | --- |
| Round 1 | Administrators (institution); community partners; extended health care providers (e.g., insurance, RTW/LTD); physicians & HCPs; policy makers; QEPs; researchers; survivors & care providers |
| Round 2 | Administrators (healthcare institutions); community partners & practitioners; HCPs; policy makers; QEPs; researchers; survivors & supporters; third-party healthcare insurers |
| Round 3 | Administrators (healthcare institutions); community partners & practitioners; HCPs; policy makers; QEPs; researchers; survivors & supporters; third-party healthcare insurers |

| **Round #** | **Theme ‘K’ Impacts** |
| --- | --- |
| Round 1 | 1. Increased & sustained exercise engagement across cancer survivor groups via greater accessibility to dynamic intervention support opportunities 2. Establishment of a comprehensive base of exercise support services to accommodate the variable needs of cancer survivors & promote their independence to self-manage their condition via exercise |
| Round 2 | 1. Increased self-efficacy, engagement & sustained participation in exercise across individuals & groups of cancer survivor groups via existing & novel exercise support approaches & resources 2. Establishment of varied & effective exercise support strategies to meet the needs of diverse cancer survivor groups (e.g. demographic, cultural, geographic, behavioural) & promote their independence to self-manage their condition via exercise |
| Round 3 | 1. Increased exercise self-efficacy & sustained exercise participation in cancer survivors via existing & novel exercise support approaches & resources 2. Establishment of numerous effective exercise support strategies to meet the needs of diverse cancer survivor groups (e.g. demographic, cultural, geographic, behavioural) & promote survivors’ independence to self-manage their condition with exercise |

| **Theme L** |
| --- |

| **Round #** | **Theme ‘L’ Title** |
| --- | --- |
| Round 1 | High-priority ‘patient-level’ and ‘economic’ outcomes for community (e.g., federal, provincial, institutional) health administrators |
| Round 2 | High-priority ‘patient-level’ & ‘economic’ outcomes for healthcare administrators & policy makers across sectors & levels of government |
| Round 3 | Understanding the high-priority ‘patient-level’ & ‘economic’ outcomes for healthcare funders & decision-makers |

| **Round #** | **Theme ‘L’ Goals** |
| --- | --- |
| Round 1 | 1. Increase &/or secure permanent funding & infrastructure support for exercise as a standard of care in oncology 2. Understand the unique patient outcome & economic priorities of key individual stakeholder groups 3. Evaluate efficacy of existing or emerging cancer exercise services in relation to patient outcome & economic priorities 4. Communicate efficacy findings to stakeholders to secure permanent support |
| Round 2 | 1. **Research / Dissemination:** Understand & promote which unique patient outcomes are priorities of administrators & policy makers from healthcare institutes, third-party insurers, & government agencies 2. **Research / Dissemination:** Understand & promote which unique economic / financial outcomes are priorities of administrators & policy makers from healthcare institutes, third-party insurers, & government agencies 3. **Research:** Evaluate efficacy of existing or emerging cancer exercise services in relation to patient outcome & economic priorities 4. **Uptake / Implementation:** Optimize communication between: (1) survivors & supporters, QEPs, HCPs & researchers & (2) administrators & policy makers from healthcare institutes, third-party insurers, & government agencies to secure permanent policy & financial support for exercise as a standard of care in oncology |
| Round 3 | 1. **Research / Dissemination:** Identify & promote the high-priority (1) patient outcomes & (2) health economic outcomes of healthcare funders & decision-makers (e.g. administrators & policy makers from healthcare institutes, third-party insurers, & government agencies) 2. **Research:** Evaluate whether existing & emerging cancer exercise services can improve the identified high-priority outcomes of healthcare funders & decision-makers 3. **Uptake / Implementation:** Optimize communication between healthcare stakeholders & (1) survivors & supporters, (2) QEPs, (3) HCPs, & (4) researchers to secure permanent policy & financial support for exercise as a standard of care in oncology |

| **Round #** | **Theme ‘L’ Stakeholders** |
| --- | --- |
| Round 1 | Administrators (institution); community partners; extended health care providers (e.g., insurance, RTW/LTD); physicians & HCPs; policy makers; QEPs; researchers; survivors & care providers |
| Round 2 | Administrators; community partners & practitioners; HCPs; policy makers; QEPs; researchers; survivors & supporters; third-party healthcare insurers |
| Round 3 | Administrators; community partners & practitioners; HCPs; policy makers; QEPs; researchers; survivors & supporters; third-party healthcare insurers |

| **Round #** | **Theme ‘L’ Impacts** |
| --- | --- |
| Round 1 | 1. Permanent funding to support exercise as a standard of oncology care 2. Establishment of patient outcome & economic priorities for exercise oncology research 3. Creation of research summaries (e.g., whitepapers, cost analyses) to advocate for the establishment of permanent funding & infrastructure support 4. Enhanced stakeholder commitment to support exercise as a standard of care in oncology |
| Round 2 | 1. Establishment of high priority patient outcomes to inform public health policy & financial resource allocation towards supporting exercise as a standard of care in oncology 2. Establishment of high priority economic / financial outcomes to inform public health policy & financial resource allocation towards supporting exercise as a standard of care in oncology 3. Evidence supporting the efficacy of medically supervised & community-based exercise oncology programs & support services to improve the identified high-priority patient & economic / financial outcomes 4. Creation of effective communication pathways to disseminate & discuss research findings with administrators & policy makers from healthcare institutes, third-party insurers, & government agencies to advocate for the establishment of permanent funding & infrastructure support for exercise as a standard of care in oncology |
| Round 3 | 1. Increased awareness of, & research targeting, the high-priority (1) patient outcomes & (2) health economic outcomes of healthcare funders & decision-makers to inform public health policy & financial resource allocation towards supporting exercise as a standard of care in oncology 2. High-quality evidence supporting the efficacy of medically supervised & community-based exercise oncology support services to improve the identified high-priority patient & health economic outcomes 3. Regular communication & collaboration between stakeholders to optimize the development & conduct of exercise oncology research that directly supports the establishment of permanent funding & infrastructure support for exercise as a standard of care in oncology |

**Survey Round 2 – Open-ended Responses**

| **Round 2 Themes** | **Open-ended responses** |
| --- | --- |
| Theme A | - Becoming a standard of care will be predicated on research specifically on successful phase III trials. This a high bar. Identifying preferred manners of communications can go a long way in getting the message out. - Communication is a hugely important aspect - Communication re the benefits / value of exercise is key & should include the medical & support community (stakeholders) - improve their understanding & they will aid in communicating with survivors - Consider language & cultural barriers as they relate to communication. These factors could impact how messages are received by survivors. - Half the battle is FINDING these resources in my opinion which is why I put it as such a high priority for exercise support. - I wouldn't start the list with "risks" - I’d start with benefits, then barriers/facilitators, then risks. Semantics, but how it's presented/communicated matters! - Important but if done without contribution of other supports/tools/implementation to providers, impact potential low - Is it possible to find another term than "survivor"? Decades of war on cancer rhetoric have turned me off that term. It’s not a war. I'm not a survivor. I live with a condition that once threatened my life & may do so again. Survivor is a short-term concept, an end point. How about people living with cancer? That emphasizes the process of our lives with the disease. - It all depends on the format & number of communication interventions (i.e. posters & pamphlets could be a good addition to a conversation with my doctor, but could not be the only form of communication received) - It's an empowering approach for cancer survivors - Medical advice regarding this research priority was totally lacking during my treatment & post treatment, I had to search it out for myself. - Particularly risks & benefits for specific understudied populations - Region specific programs are very important as each region varies in what they can offer. Not everyone has access to a Wellsprings centre. Motivation help will be key as many survivors might feel fitness is not a key priority in the health at that moment because of the effects of chemo. Motivation can help realize exercise can combat the effects of chemo. - This research priority frame can provide a whole picture of cancer survivors care. - Very useful. Best not to treat all people the same in what is a complex set of conditions & treatment types - Whilst exercise engagement is an important part of occupational performance roles, but it would be beneficial to broaden the priority to include the balancing of other occupational performance roles important to the individual to assist with balancing an increase in exercise engagement. |
| Theme B | - First diagnosis, a person came to me, specifically to talk about the exercise & the programs available at the wellness center close to the hospital. Second diagnosis (I don't know why) no one came to talk about exercise with me. So, for hospitals with no specific program, that information has to be provided by medical team - Giving tools for how to support patients is important to be able to do so - Having HCPs on board with this is incredibly important. Having the backing of oncologists, PCPs, nurses & others are crucial in influencing survivors to exercise. - I think engaging HCP's might be challenging. As there are already issues surrounding them providing the correct & available resources in general for a cancer diagnosis exercise might just be one that falls through the cracks. For example, a young adult who gets diagnosed with cancer may be referred to support groups that basically have everyone 55+ but no one their age. So, if there is another resource for HCP referrals is developed how can you ensure it gets used & ensure it gets used to the appropriate target audience. - Maybe discuss the social aspects of group exercise for survivors? - My oncologist is extremely knowledgeable, but never mentioned exercise & did not encourage it or tell me to adapt. HCP education is needed. - Oncologists a key group. All other HCP are important in supporting the message coming from the oncologist. - One of the big issues around physical activity & cancer is the lack of knowledge & understanding at a professional level (which ultimately fails to support the cancer population). The natural pathway seems to be one of caution & "rest" & finding the appropriate balance for patients takes knowledge & skill - The more evidence-based studies available to support the goal, the better - There is lots of good pre-existing education material so will be more about pulling it together rather than creation of new I think - Very useful. Not all professions are equally trained in exercise of oncology but have differing opportunities to input into the care pathway at differ by stages. Effective training that is suited to the particular profession is important - Whilst exercise engagement is an important part of occupational performance roles, but it would be beneficial to broaden the priority to include the balancing of other occupational performance roles important to the individual to assist with balancing an increase in exercise engagement. - Why 'Diverse' in front of HCPs? it's not addressed anywhere in the rest of the document. |
| Theme C | - As someone living in real Ontario with little access to large cancer centres & their resources, the idea of a local QEP being able to add this sort of education to their training would help reach more survivors like me. - Define & embed the standards also important! - Defining boundaries is important to recognise the different training & expertise of health professionals i.e. physios in the management of things like spinal metastasis & sarcoma - Diverse QEP & various environments are too vague concepts. Would be interesting to know how this would be articulated concretely (Partnerships with gyms, specific gym / center, specific QEP in generic gym?) - My opinion is that somebody is either a QEP or they aren't. A professional who provides exercise oncology support within the scope of their profession is probably more accurate. - I appreciate how standardised education for HCP would be key, there are many routes into this area that aren't open to some professionals or are costly. - If you solely focus on the training at the post-secondary level, you could be missing a wide range of fitness professional. What are the possibilities of designing a continuing education course for certification bodies CSEP, CPTN, ACE, CanFitPro. - It is also important to develop strategies to foster communication between the stakeholders - This is possibly one of the most important areas of this becoming a standard level of care. Having standards for things like, who is able to train survivors, who oversees trainers, etc. is important. - This would be a vast & challenging area to train on, but very important. I have found it most helpful when I have exercise professionals who understand that my body no longer operates the way a “normal” one does - Through my recovery I’ve been trying to find people to help with effective rehabilitation. No one is willing to say they aren’t qualified to help & I keep getting hurt. So, effectively qualified people are imperative. - Use of diverse here - again, just QEPs -then define them as a diverse group (personal trainers, group fitness, CEP, etc.) - We live in a society of variation & inequality when it comes to accessing rehabilitation & physical activity for cancer survivors. This needs addressing to improve support for patients & increase understanding. - While I believe this is important, I am not sure that it will really help it be a standard of care - Whilst exercise engagement is an important part of occupational performance roles but it would be beneficial to broaden the priority to include the balancing of other occupational performance roles important to the individual to assist with balancing an increase in exercise engagement. - Who develops standards? Who enforces them? Education & Educational materials are, I think much more important than more standards |
| Theme D | - 3hr drive from my treatment hospital in good weather. Resources I have been able to have access to online, is often, all I have access to. - Access is important & has been demonstrated during the Covid era. It shows we can reach out through new means when we need to & that people are receptive. - COVID has shown great uptake in online exercise so it is an opportunity to reach people who live more remotely or who are embarrassed to exercise in public or groups - COVID means we have had to change the way that we work & that has accelerated a reliance on technology. In a cancer population whilst social distancing remains particularly important using other means of technology is increasingly important to facilitate people to access services but relying entirely on technology may mean factors get missed or overlooked that help support people best - Especially important during COVID - Even more important now during the COVID-19 pandemic - Exercise is physical. Technology can help people/stakeholders to track the data, but it must be remembered, that, first & foremost, people need to move. Motivation of people living with cancer, understanding the impact movement can have on mental & physical health, understanding how to modify exercise for the patient etc. are all much more important than data. - I had trouble with this one as it will be very important to help some of the cancer survivors but not all - I think there is good research out there that supports technology as a means of support for other areas of care, so this sound be the same for exercise. - I think this is important for reaching certain subpopulations of survivors. This would be incredibly helpful for rural populations & even survivors who see going to a gym as a barrier. - Links with electronic patient records important for clinicians - Online systems such as Zoom provide a great way to bring exercise programs to people with a cancer diagnosis who prefer not to participate in onsite community-based exercise programs during a pandemic. - Technology should be embedded across the research opportunities. As evidence during the pandemic, it's critical. & yes, likely research 'on it' per se - but it should also be integrated across training QEP, communicating to CS & HCP, etc. - Thank the pandemic for bringing incredible attention to this approach to providing these services to survivors. - This would be helpful, especially for those outside of central geographic areas, but how would it be customized? - This would enable wider engagement - To help adherence & especially in the covid era - Unfortunately. I believe that having ‘self-management’ tools for exercise training plans will likely be more palatable from a funding perspective but I don’t think it’s the most effective tool for ALL survivors. Many cases would likely be better handled with direct support from a human, maybe who uses technology as an assistance tool. I don’t necessarily think technology should be THE help. - Wearable devices can provide a lot of support & motivation for the survivors, but there are issues of accessibility & privacy. - With the recent COVID-19 pandemic context, efforts are made to reduce the exposure of cancer survivors to the virus by moving in-person visits to telemedicine or otherwise redirect non-acute care. - Would definitely help in standardizing information given to survivors, as well as accessibility to information |
| Theme E | - Although I see this priority as extremely important my concern about integrating another professional into the care team might not be as effective as you expect. A cancer patient sees so many people in one appointment & this could become overwhelming. Not to mention the care teams are often already understaffed with nurses & care coordinators so the need for an exercise professional might be questioned. - Especially at the point of initiation, it would be helpful to have a QEP within the primary team to normalize it as a standard part of oncology care & also to provide survivors the best shot at maintaining their physical health at the onset of treatment. In my case, waiting two years after the fact has likely made my recovery much harder than it needed to be. - I had a medical team to treat my cancer but once they felt like they were done their job I was left on my own. If there had been a QEP along with me for the ride I would have had someone who knew me pre, during & post treatment which would have been helpful. - I have found that Drs & NPs are not that comfortable with giving exercise advice - I think exercise professionals will add to the MDT but are not the only profession to help with this aspect. I think cancer exercise education should much more explicit in the training of exercise professionals. - I think this research priority if very important, however I do think there needs to be a way to standardize this as I used to work at several hospitals & the "exercise care" was not up to par. - More medical practitioners should promote lifestyle changes such as "exercise is medicine" instead of always resorting to prescribing drugs. - Need cancer care systems to recognize role of QEP. - Research demonstrates that being globally fit improves responses to treatment & outcome afterwards, in herbal. More research is useful to demonstrate this in varied cancer types & treatment/stages. - Restate the priority & lead with the word Integration - Scope of practice -- what do you mean by state law? Are you referring to national law? or are you borrowing from American guidelines? - Second Question is complicated...If we do have QEPs in place we do have reached the goal of being part of standard care - Specify these QEP only see oncology patients or other non-oncology patients too? Might want to specify - These QEP should act like hospital social workers do, they have advanced knowledge in their field, but are also gate keepers to the community resources for their patients for exercise & also empowering & supporting survivors to access them for long term impact & attendance by survivors - Using a collaborative & interdisciplinary approach to healthcare & in this case survivorship is crucial. - Very important given the large cancer survivor population & the shortage of HCPs to work with cancer survivors. Problem-Limitations imposed by the practice acts of the individual health professions. - While extremely important & likely to help, it is extremely unlikely that this will be funded or supported anytime soon, so the focus would need to be heavily on the 'research' side to show efficacy & cost-effectiveness - I don't think #3 should be listed (yet) - With appropriate & adequate training QEPs could have an important & more cost-effective role in this |
| Theme F | - A referral pathway creates a power dynamic. If one or two people are responsible for referring, this then can lead to only certain programmes being referred too. I think choice should be given to the patient to choose what, when & where suits them. - Access to PA services at every stage of the journey is important along with the confidence that a joined-up pathway provides to survivors - As mentioned previously, finding the help is the hardest part. Integrating referral programs to assist survivors in finding the help would make life a lot easier. Especially those actively in treatment who may be suffering from fatigue & have a hard time dedicating energy to such a (tedious) task. - Highly important. Many cancer survivors do not know the resources available to exercise during their cancer journey - In my opinion the referral pathways are an administrative support mechanism & will vary greatly according to the program setting without necessarily impacting outcomes. - Medically supervised / community-based groups play a vital role. Those that require more support are people who will often struggle to access physical activity independently. Having settings that give more support even just for a short period of time can build confidence & understanding in a vulnerable population giving them the tools to do these things more independently longer term - Referrals as in the hospital sets an appointment for you at an exercise facility. That would be awesome, since when in treatment, you don't necessarily have the mental space to think about that or the motivation to call yourself. So, if appointments are made through the hospital that could help integrating exercise as a standard of care. Also, if taken care of by the hospital, it shows the patient that exercise is part of the care - Requiring participation in medically-supervised settings for cancer survivors may create a barrier to regular physical activity participation in unsupervised settings, especially since initial medical screening/approval is not needed prior to a cancer survivor wanting to take part in light-moderate intensity PA - There is a huge gap there right now. In my area they do not have any exercise programs for oncology - Think this is part of the last question around QEP in care pathway. if you have them there, referral is part of that pathway. See Santa Mina Curr Onc pathways article for a model (don't reinvent the wheel). Similar to earlier comments, this needs to be integrated across implementation science projects as part of the methods. Not sure it's an 'independent research priority' (& really, how many research priorities can you have? want to keep it focused, & build methodologies (in care pathway, using tech) across the specific research foci. - This is potentially the most important. However, these pathways need to be SIMPLE & involve a central cancer exercise professional to sort out the referral rather than a complex decision-making algorithm - This is their way in! - Triaging survivors to HCP that meet their needs is currently the biggest stumbling block in the entire process. - Where to refer to & who to where is often still unknown - Without clear pathways people won’t be referred |
| Theme G | - 2nd most important. Can’t have a referral pathway without places to refer to - Absolutely essential to target those who need it most! - Access to diverse groups & peer support is very important for patients to adhere to exercise. - I don't think a community exercise class would have encouraged me much, I find at times there's a odd competitive component with a bunch of survivors get together. Sometimes there's a bit of a battle to see who had it the worst or who had it better. I need to build myself back up at my own speed. - I put both in a neutral because the hard to serve & the underserved can be very different. Implementing an exercise program for the hard to serve might not be possible as they are already struggling to get basic primary & even cancer care at its core. However, the understudied population could be easy to reach just not viewed a priority in the research. - I think this is incredibly important & using a model such as cardiac rehab is useful to reference. - I think we know tons about preferences - can't keep reinventing the wheel. we need to focus on implementation with the underserved groups, as example. - It is no secret that low sociopath-economic people are higher risk of many things including poor health outcomes. Accessibility & support would give them a much better chance at best case outcomes. - The absence of such services in most communities is well recognized. More data will help buttress an already recognized problem. - There is a reasonable amount of data out there on goal #1 in terms of needs & barriers, likely ready to move onto next parts of the goals - Third party healthcare insurers should consider reimbursing people with a cancer diagnosis who choose to eat a healthy diet & exercise regularly to make long lasting lifestyle changes in addition to reimbursing survivors for drugs. - This relates to the previous question. Most survivors do not require a medically-supervised intervention, just an appropriate referral - Whilst exercise engagement is an important part of occupational performance roles but it would be beneficial to broaden the priority to include the balancing of other occupational performance roles important to the individual to assist with balancing an increase in exercise engagement. |
| Theme H | - Collaborative working would be vital to help streamline services & experiences - Communication & collaboration between professionals is always helpful, no matter where in the world you are. Why would you want to be doing the exact same research separately if you could be working together with more minds & funding? - I think some networks exist, & exercise oncology guidelines exist. so not sure that this is a research priority per se, but can we do it better? must we do the collaboration better? YES - I'm unclear on exactly what this goal is, seems quite broad - resources for patients? HCP? do we not already have this through "moving through cancer" website? - Is important but perceive this would happen anyway through conference & publication so graded lower for impact - Local "cultural" differences are extremely difficult to overcome. Regional & international differences would be almost insurmountable. & to what end? What works in Arkansas may no chance of effectives in sub-Saharan Africa. - Not sure I understand what this means - Sharing approaches, learning from each other & collaborating are key to the development of exercise in cancer care. I would though want this not to be of an academic nature. A robust study into exercise is not always the best exercise approach. Local community-based schemes don't have the funding for an academic approach nor is that a skill set in those trainers sometimes. - This is fine as long as there's peer review in both directions - This priority is addressed to QEP mostly, & important to them in my opinion. |
| Theme I | - Although I see this as important, I would see this as the same as other aspects of care. There is always transitions from your academic health sciences centres to your community hospitals to your primary care so that could be part of the transition program - Could this potentially link into previous priority about pathways rather than being separate as they are closely related - Education on the impacts of exercise is more important than transitional help. Transition goes on far more naturally when properly educated on the importance & impacts. - Giving people the tools to feel confident & knowledgeable around their cancer diagnosis is vital & exercise is a massive part where they can have ownership over this. Giving them a phased option can for many patients play an important role to exercise independence & confidence. There should be recognition that not all people will require this approach, some will simply need the info to get them started with little other support - Graded lower as think this is covered in some of the earlier priorities - Home-based exercise with regular coaching might benefit a lot of people living with cancer. Often it is hard to get to a class, or you don’t want to be around other people, or its expensive. Lots of people like to exercise from home, especially since so much can be done with minimal equipment &/or using things normally found in a home. - I think this is critical - & lots of this is starting with the CSTG that were awarded this past year. So, across the research priorities, think scans should be done to see WHAT IS ALREADY being done. This is a focus in some large team grants, so don't want to think that it's a priority because it's not being done yet (which isn't the case). - Important for long-term adherence - Once again, I believe this to be an administrative variable - Smooth transitions across different care settings is essential for continuity of care, beneficial outcomes & reduction of a non-medical stressor. - This is a good initiative, but we need to determine what encompasses medically-supervised vs. self-directed PA programs since these are not yet common practice in cancer survivors (unlike phase 1 & 2 cardiac & pulmonary rehab programs) - This priority is not clear - This relates to the previous two questions on access & referral pathway - This seems like such a pipe dream from my experience I'm not sure how to answer the question. I was given no advice from my medical team other than 'eat right & get some exercise' when I completed active treatment. - Timely transition important for long term sustainability financially - While important, I think if use of hospital + community based resources exist & there is an increase in number of attendees to numbers that are difficult to manage (a good thing) this part may more naturally occur & so while important, is less of a priority compared to the others listed here - also this is likely to be very individualized based on setting & needs, so hard to study for broad application |
| Theme J | - Accessibility is the key, and money is the key to accessibility...! - Again, important but elements are covered in some of the others. cost analysis always important for sustainability - Being done?????? I think much of this effectiveness work is being done, & reviews on it exist...so not sure it's a current priority??? - I currently take advantage of a community-based cancer exercise program. The instructors are trained to work with people with a cancer diagnosis which means they are aware of the need to modify exercises to meet different needs. It's a very supportive (& challenging) program which I highly recommend. - I know in my community there is not the population to make this sort of program feasible so I would put this as much lower importance than technology-based ways of supporting survivors that could reach a larger geographical area. - I think cost is very important for survivors & outcomes for policy makers - If we don't know cost, how will we ever make these standard care - Really important. Feasibility & safety matter to the patient. - Screening is good. But it must be emphasized that screening is not diagnostic how does the screening organization insure required follow-up? - There's a lot here with everything from feasibility all the way to effectiveness. my opinion is that feasibility of community programs has been demonstrated & no further research is needed here. It's unclear what kind of efficacy study is being undertaken in community programs if there are diverse survivors, so I think the focus here is safety & effectiveness as a priority for community programs. - Unless HCP are promoting & suggesting community programs it will likely not be standard of care - We need rigorously designed, sufficiently powered (accounting for the more modest changes seen in these types of interventions) trials that have clearly identifiable confirmatory endpoints (i.e. prespecified outcomes with preregistered statistical plans). |
| Theme K | - Do not understand this priority. It’s all words no meaning! - Facilitation & diversity are the key terms here - From my experience in the breast cancer world there are SO MANY treatment variables, cancer types & sub-types as well as all the differences in patients I'm not sure a prescription for all breast cancer patients would work. - I feel like these Research Priorities are all very similar. Having a difficult time deciding which ones are more valuable than the next - I think implementation is extremely important, but as worded this title & objective are unclear to me what exactly is suggested. It is extremely vague. - It is always about the money!!! Importantly, funding must be reliable. - It seems from preliminary research already conducted that sustained exercise programs are most beneficial over the course of anyone’s lifetime, so this is super important to give survivors the best shot at best case outcomes. - Long term changes in health behaviours is important but wonder whether the wording needs tweaking to really highlight this appears more related to the long-term patient sustained change - Rigorously designed studies that are grounded in behavioural change theory are needed. - This is embedded across other research questions??? |
| Theme L | - Again, money is the key... & having the stakeholders understanding how beneficial exercise is in the survival rates & quality of life is crucial. - Done within the pathways/establishing QEP in team/referral q's - these are one component of that research, to make sure KT (key players involved from admin) & establishing outcome measures (economics must be included). So, don't see it as a separate research priority. - Each of these groups will have their own perception of what constitutes economic outcomes. Generating consensus is only a dream. - Extremely important but less feasible than other options - Hopefully this is using a patient-centered approach. - In order to get any of these fabulous ideas off the ground you're going to have to convince the policy makers that they're economically viable or patients will never benefit. - It is good that this question investigates the effect on the person as well as broader health economy - Medical professionals & policy makers will be very interested in these outcomes & may be what is needed for change. - Outcome measures can be challenging in a cancer population, particularly if you are using them in a more palliative setting. Getting commissioners to understand that more quantitative measures are important - Outcomes are really important but the wording in the priority emphasises exploration of outcomes of value to investors rather than the patients - perceive this needs to rephrased to provide equal value to patients & stakeholders. then would have scored higher - Simplify to health economics this is too wordy & makes no sense - This step could revolutionize the way insurance packages are developed. If there’s greater incentive to get & stay healthy, insurance companies will be more likely to support preventative programs (& prevention of recurrence/secondary cancers). - Those who make financial decisions need to understand the value in exercise & it can shift the money put into exercise at a facility level. Having resources onsite will encourage HCP to recommend exercise. - We need decision makers to believe in exercise oncology otherwise none of it will work |

**Survey Round 2 – Research & KM Theme Suggestions**

| **#** | **Title** | **Goal(s)** |
| --- | --- | --- |
| 1 | Patient/caregiver accessibility to Technology-based exercise learning | Patient access to an iPad & internet access to training/support |
| 2 | The adoption of transparent & open research practices | Promotion of appropriate preregistration, improving transparency & detail of reporting both in preregistrations but also reporting of results, clear distinctions between confirmatory & exploratory outcome analysis, & the providing of open code & data as a default not "upon reasonable request". |
| 3 | Virtual exercise prescription | To provide virtual exercise prescription to patients undergoing treatment from their own homes |
| 4 | Is exercise prescription for weight loss during chemotherapy safe & effective for obese patients? | Establish if fat weight loss can improve outcomes for obese population. |
| 5 | Psychological & nutritional support for cancer survivors in the community setting | Training & informing cancer survivors to manage their psychological & nutritional strategies in detail |
| 6 | Comparative effectiveness of video versus face to face consultations & exercise prescription | Study the barriers to virtual exercise prescription, population limitations, & risk management strategies related to virtual consultations |
| 7 | Evidence-based practice specific to the demographic | Considering economics, medical resources, geographical landscaping & population to develop effective community-based exercise programs |
| 8 | Telehealth | Feasibility of it |
| 9 | Consider the spectrum of exercise options for cancer survivors | Identify & report on non-traditional exercise programmes for cancer survivors. |
| 10 | Barriers to virtual care? | Needs assessment, barriers? |
| 11 | Effectiveness of virtual Exercise programs | Determine the relative effectiveness of virtual exercise programs compared with traditional in person programs |
| 12 | Use of a Virtual Exercise Program by Patients | To engage cancer patients & survivors in their home doing simple movement to just get the moving during isolation |
| 13 | Exercise when keeping social distance | To identify the type of support cancer survivors need to exercise alone or at home when group exercise is not an option |
| 14 | Exercise buddies for virtual group workouts. | Establish partnerships &/or virtual coaching to promote accountability & motivation to exercise. |
| 15 | Who is benefitting from exercise? | Why do we have responders & non-responders...how to change a non-responder into a responder |
| 16 | Living with Cancer at home | Learn how to support people living with cancer in their home environment 2. develop strong communication links beyond the hospital or clinic care setting |
| 17 | Lymphedema Care | Public knowledge & health care recognition |
| 18 | Exercise during Covid | Maintain an exercise protocol at a safe distance during a pandemic |
| 19 | The increasing role of using technology during a pandemic | Maximize technology as a primary delivery system |
| 20 | Collaboration with Benefits Providers to Implement Cancer Prevention Programs | Encourage people to engage in physical exercise programs that will help prevent cancer in the first place. |
| 21 | Exploring remote & virtual technology to support adherence to exercise programmes. | Many programmes have had to move to virtual programme. What platforms work best for patients & is this in a group or individual setting. |
| 22 | Individualization of training concepts for inpatients | Remove calculated models from treatment (e.g. calculated heart frequencies or thresholds) |
| 23 | Health care provider (e.g., nurses, physicians), caregiver, & patient education on importance of exercise during the cancer continuum | Providing educational tools for HCP, caregivers, & patients so they can make informed decisions about exercise. |
| 24 | Home-based, insurance approved visits from a QEP | To ensure supervised at home learning/ activity to keep the high-risk survivors active |
| 25 | Improvement in statistical methods adopted in exercise oncology trials | Performing sufficient & realistic sample size calculations (most meta-analysis show small to moderate effects of exercise in cancer populations), use of blinded & independent statisticians, appropriate intention to treat analysis, prespecified statistical analysis plans, careful & appropriate interpretation of p-values, more control for multiple comparisons, & exploration of non-frequentists approaches if they best answer the question posed. |
| 26 | Are group exercise classes safe in the oncology population? | Establish benefits vs negatives in group exercise classes. |
| 27 | Cancer survivor, user friendly application for smart phone | Maximizing effectiveness of using smart phone apps to help cancer survivors follow their exercise program |
| 28 | Cost effectiveness of virtual screening for exercise needs | Study the scope of service delivery & comparative costs to patients & health care institutions |
| 29 | Integration of virtual practice in education of QEPs | Development of education regarding protocols & procedures for cancer-based exercise prescription |
| 30 | Virtual social support for exercise motivation & accountability | Determine effectiveness of fitness social support via technology (virtual mechanisms) |
| 31 | How to exercise effectively in a pandemic | Communication about safe & effective exercises while remaining at home |
| 32 | Technological support in home-based exercise | To identify what type of technology (e.g. interactive video gaming) can support cancer survivors to exercise alone or with family members |
| 33 | Financial incentive with wearable sensor exercise prescription. | Exercise oncologists can prescribe goals for patients & monitor with devices. Can motivate with tax rebate for completing the programming. |
| 34 | Supporting people living with cancer | Support patients as they transition from treatment to living with the disease -- its harder & more complex, the first goal of immediate survival has succeeded, but the fear of becoming a patient again is very alive. How to manage the long term? |
| 35 | Rare cancer- exercise prescription | Safety & effectiveness |
| 36 | Research on the interactions of exercise with cancer therapies, especially with respect to timing of exercise sessions relative to receipt of treatment. | Examine how lack if exercise, especially during these times, affects our mental health |
| 37 | Supporting behaviour change to promote adherence & also empower the patients to take control of their exercise regimens | Having the difficult conversations, using the teachable moment. Probably even more important in this virtual world. |
| 38 | Removing barriers in clinical settings for exercise therapies | Recognize & remove "stop signs" for more activation |
| 39 | Understand the effect of trained QEPs on exercise & health outcomes during cancer treatment | Improved patient outcomes, decreased direct & indirect care costs |
| 40 | A more mechanistic approach to exercise oncology | Larger collaborative groups may be necessary to explore the mechanisms for how exercise may improve important outcomes in cancer research, e.g., fatigue, HRQoL, depressive symptoms, treatment toxicities, etc. |
| 41 | Conversations with HCP's about the patient exercise experience | Find out if the HCP is asking about the ability for the patient to be exercising & moving within their home while isolated |
| 42 | Stem cell transplant & exercise prescription | Standard of practice for exercise during Stem cell transplant |
| 43 | Flexible delivery of programs | Availability of in person & virtual supports |
| 44 | How nutrition goes hand in hand with exercise in exercise oncology | Many patients talk about nutrition & how this is as important as exercise. |
| 45 | Research different exercise pathways or options based on a patient's cancer type & treatment | Personalize exercise options |

| **Incomplete Suggestions** | | | |
| --- | --- | --- | --- |
| **#** | | **Title** | **Goal(s)** |
| 1 | Combine some of the research priorities together. We should have 2-4 priorities, then "research needs" follow that (i.e., other issues, but not priorities). I think overall an update to what is currently happening should occur, ID gaps, then set just a couple priorities. there is overlap (substantial) between many of those suggested! | | Not reported |
| 2 | Virtual exercise programming for cancer survivors | | Not reported |
| 3 | Cross collaboration across centres to share resources & strategies/access to programming that would have previously been limited to one site | | Not reported |
| 4 | Which virtual platforms work best for providing adequate exercise to oncology patients | | Not reported |
| 5 | Sustaining long term programmes | | Not reported |
| 6 | Research on the best exercise prescription to target clinically relevant outcomes. For many outcomes we do not have sufficient evidence to prescribe the correct dose of exercise. No drug would be implemented as standard of care without evidence for optimal dosing. | | Not reported |
| 7 | Effectiveness of exercise programmes for Adolescent and Young Adult cancer survivors. | | Not reported |
| 8 | Does mental health challenges during a pandemic change patient's priorities? | | Not reported |
| 9 | Training for virtual exercise programming for cancer survivors | | Not reported |
| 10 | Mental health & exercise | | Not reported |
| 11 | Sustaining virtual groups | | Not reported |
| 12 | Does a virtual program have the same effects as an in person one | | Not reported |
